# Responses of Transgenic Melatonin-Enriched Goats on LPS Stimulation and the Proteogenomic Profiles of Their PBMCs

**DOI:** 10.3390/ijms19082406

**Published:** 2018-08-15

**Authors:** Minghui Yang, Jingli Tao, Hao Wu, Lu Zhang, Yujun Yao, Lixi Liu, Tianqi Zhu, Hao Fan, Xudai Cui, Haoran Dou, Guoshi Liu

**Affiliations:** 1 National Engineering Laboratory for Animal Breeding, Key Laboratory of Animal Genetics and Breeding of the Ministry of Agriculture, Beijing Key Laboratory for Animal Genetic Improvement, College of Animal Science and Technology, China Agricultural University, Beijing 100000, China; Yangmh16@cau.edu.cn (M.Y.); taojl16@cau.edu.cn (J.T.); wuhao7815@cau.edu.cn (H.W.); luzhang2018@cau.edu.cn (L.Z.); yujunyao1995@gmail.com (Y.Y.); lixiliu93@gmail.com (L.L.); zhutq2017@gmail.com (T.Z.); Fanhao@cau.edu.cn (H.F.); 2 Qingdao Sanuels Industrial & Commercial Co., Ltd., Qingdao 266000, China; suxingdemogu@gmail.com (X.C.); caylapangpang@gmail.com (H.D.)

**Keywords:** melatonin, proteogenomic analysis, LPS, PBMCs, inflammation, goat

## Abstract

The anti-inflammatory activity of melatonin (MT) has been well documented; however, little is known regarding endogenously occurring MT in this respect, especially for large animals. In the current study, we created a MT-enriched animal model (goats) overexpressing the MT synthetase gene *Aanat*. The responses of these animals to lipopolysaccharide (LPS) stimulation were systematically studied. It was found that LPS treatment exacerbated the inflammatory response in wild-type (WT) goats and increased their temperature to 40 °C. In addition, their granulocyte counts were also significantly elevated. In contrast, these symptoms were not observed in transgenic goats with LPS treatment. The rescue study with MT injection into WT goats who were treated with LPS confirmed that the protective effects in transgenic goats against LPS were attributed to a high level of endogenously produced MT. The proteomic analysis in the peripheral blood mononuclear cells (PBMCs) isolated from the transgenic animals uncovered several potential mechanisms. MT suppressed the lysosome formation as well as its function by downregulation of the lysosome-associated genes Lysosome-associated membrane protein 2 (LAMP2), Insulin-like growth factor 2 receptor (IGF2R), and Arylsulfatase B (ARSB). A high level of MT enhanced the antioxidant capacity of these cells to reduce the cell apoptosis induced by the LPS. In addition, the results also uncovered previously unknown information that showed that MT may have protective effects on some human diseases, including tuberculosis, bladder cancer, and rheumatoid arthritis, by downregulation of these disease-associated genes. All these observations warranted further investigations.

## 1. Introduction

Traumatic injury, infective-response toxin poisoning, and autoimmune diseases are often accompanied with inflammation [1,2]. An adequate inflammatory response promotes protection of the host against the noxious stimuli and initiates the defensive healing process. However, an excessive inflammatory response results in further tissue and organ damage [3]. For example, the pathological progressions in acute pneumonia and septic shock are closely associated with excessive inflammatory reactions [4,5,6]. When the invading pathogens are detected, various immune cells infiltrate into the infected regions to clean the invaders. Among these immune cells, peripheral blood mononuclear cells (PBMCs) are very important to mediate the inflammatory response [7]. PBMCs accumulate in infected tissues to remove infectious agents and clear dead and injured cells. They closely regulate host defenses, for example, to initiate the inflammatory response locally or systematically [8]. Lipopolysaccharides (LPS) and tumor necrosis factor alpha (TNF-α) are the major factors to promote PBMCs initiating a cascade reaction of inflammatory responses [9,10]. On the other hand, PBMCs also repair inflammatorily damaged tissues by secreting various inflammation-mediating cytokines, chemokines, and proteases [11,12,13].

Melatonin (MT) (*N*-acetyl-5-methoxytryptamine) is an important molecule secreted mainly by the pineal gland as well as immune cells and the digestive and reproductive systems [14,15,16,17,18]. This molecule exhibits pleiotropic physiological functions. These include regulating biological rhythm; improving sleep quality; adjusting the immune response; enhancing reproductive activity; and upregulating the antioxidative, anti-aging, and antitumor effects in mammals [19,20,21,22,23]. As an immunomodulatory agent, MT usually balances the immune function of organisms. It can function as a stimulator under basal or immunosuppressive conditions or as a suppressor in an excessively acute inflammatory reaction [24]. Generally, MT exhibits stimulating effects on the innate and also adaptive immune systems [24,25,26,27]. Its suppressive effects were only observed in excessively acute and chronic inflammations [25,28,29,30].

Septic shock is a systemic response of the body to endotoxins. For example, the endotoxin LPS promotes the release of numerous proinflammatory factors, TNF-α, Interleukin 1 (IL-1), Interleukin 6 (IL-6), Interleukin 12 (IL-12), Interferon (IFN), and Nitric oxide (NO), by interacting with receptors on the surface of a variety of host cells; these factors mediate extensive inflammatory reactions and cause unnecessary tissue damage [25,31]. Many studies have shown that MT can correct the inflammatory imbalance caused by LPS and promote survival rates of up to 80% in mice treated with a lethal dose of LPS [32,33,34]. The potential mechanisms may relate to MT downregulating the LPS-induced expressions of both inducible and mitochondrial NO synthases (iNOS and mtNOS), lowering the NO levels. Thus, it reduces the mortality of rats and mice treated with LPS [25,35,36]. At the cellular level, microarray analysis of gene expression also confirmed the anti-inflammatory effects of MT on Raw 264.7 cells stimulated by LPS [37]. The effect of MT on immune biology is mediated by its receptors or by receptor-independent pathways [38]. MT suppresses the LPS-induced increase in lipid peroxidation in both in vivo and in vitro animal models [39]. The beneficial effects of MT were observed in various inflammatory diseases by modulating immune cell responses [24,25,26]. Its level was negatively correlated with the seasonality of multiple sclerosis relapses, and MT treatment improved the pathological state of multiple sclerosis in an animal model [40]. MT regulates the differentiation and function of T cells and provides potential targets for the treatment of autoimmune diseases [40].

As mentioned above, PBMCs play an important role in the inflammation triggered by LPS. Up to now, little has been known about the molecular mechanisms of MT on PBMCs during the process of inflammation. In the current study, by creating an endogenous MT-enriched animal model, the potential molecular mechanisms of MT on PBMCs in LPS-induced inflammatory responses were systematically investigated in large animals (goats). The results provide valuable information on the use of MT in human autoimmune diseases.

## 2. Results

### 2.1. Effects of Melatonin on Inflammatory Response Induced by LPS in Intact Goats

**Figure 1:**
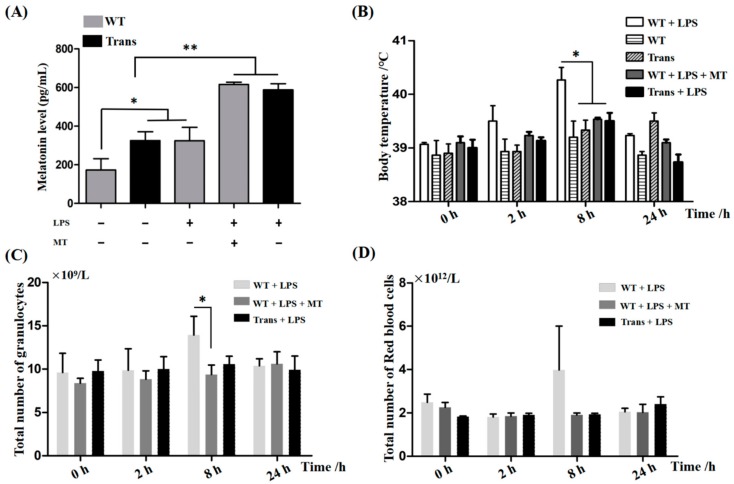
Effects of MT on LPS-induced inflammatory response in goats. (**A**) The serum MT levels; (**B**) body temperature; (**C**) total number of granulocysts in blood; (**D**) total number of red blood cells. MT: melatonin; LPS: lipopolysaccharide; Trans: transgenic goats; WT: wild-type goats. “*” represents significant differences, *p* < 0.05; “**” represents highly significant differences, *p* < 0.01.

The results confirmed that the goats had significantly higher levels of blood MT than those in wild-type (WT) goats. In addition, a MT dose was selected for injection into the WT goats to achieve similar MT levels as in the transgenic goats (Figure 1A). Some animals were treated with LPS to trigger an excessive inflammatory response. It was observed that the body temperature was significantly elevated to 40 °C in the WT goats 8 h after LPS treatment. In contrast, the body temperatures in transgenic and WT-plus-MT-treated goats did not increase compared to the controls (Figure 1B). Alterations to the total number of granulocytes in different groups showed the same trend as for the changes in body temperature (Figure 1C). The red blood cells among the groups exhibited no significant differences (Figure 1D).

### 2.2. Proteogenomic Analysis of PBMCs

The PBMCs were isolated from the blood of the goats after LPS treatment for proteomic analysis. A total of 5971 proteins were identified in PBMCs, and 5810 were quantifiable. The number of identified peptides was 44,232, and a 98,615 peptide frequency was detected. The average protein molecular weight identified in this experimental proteome was 44.26 kDa (sequence coverage of 30%; Figure S1).

**Figure 2:**
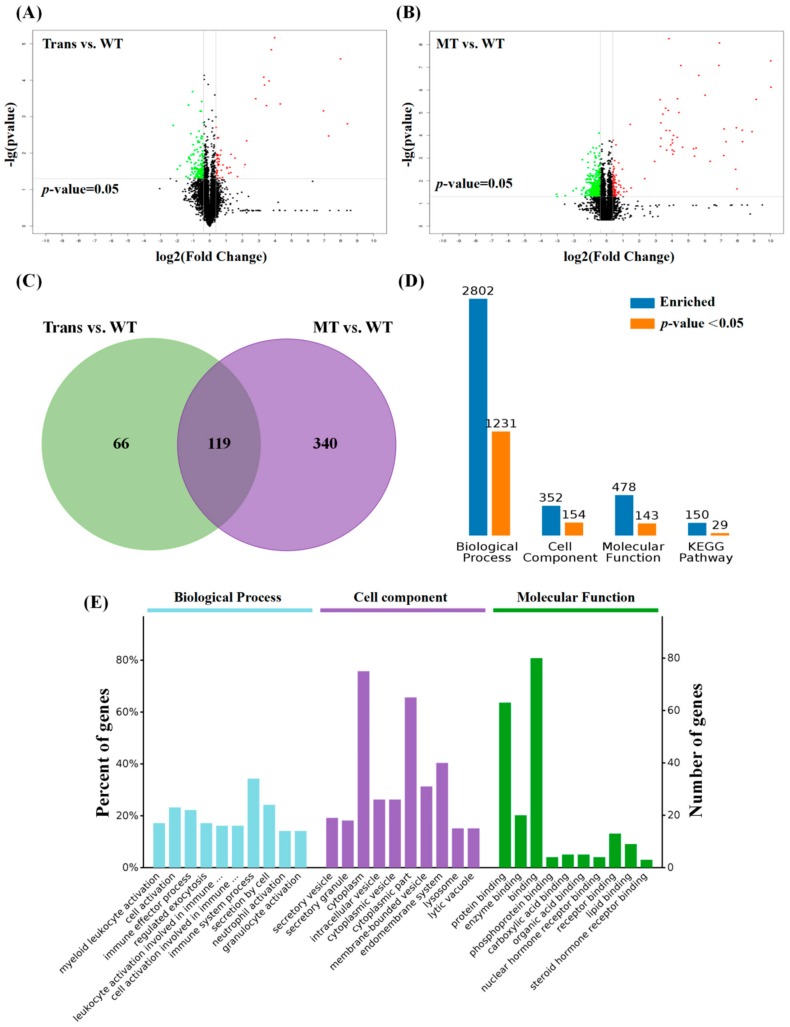
Proteogenomic analysis of the PBMCs from LPS-treated transgenic and WT goats. (**A**) Volcano plot of differential proteins (Trans vs WT): red plot: upregulation; green plot: downregulation; (**B**) volcano plot of differential proteins (WT with MT vs WT): red plot: upregulation; green plot: downregulation; (**C**) venn diagram analysis: overlapping differential expression proteins and unique differential expression proteins respectively identified by Group A (Trans vs WT) and Group B (WT with MT vs WT); (**D**) enriched GO terms and KEGG pathway from Venn diagram analysis of 119 overlapping differential proteins: blue: enriched; orange: significantly enriched; (**E**) protein expression in GO terms. PBMCs: peripheral blood mononuclear cells; LPS: lipopolysaccharide; Trans: transgenic goats; WT: wild-type goats; MT: melatonin.

Proteogenomic analysis showed that 51 proteins were upregulated and 134 proteins were downregulated in transgenic goats with LPS treatment compared to the WT goats with LPS treatment; 128 proteins were upregulated and 331 proteins were downregulated in WT goats with LPS-plus-MT treatment compared to the WT goats with only LPS treatment (*p*-value < 0.05, FC > 1.3; Figure 2A,B). Using Venn diagram analysis for the groups’ data, the following was found: Trans versus WT and WT with MT versus WT: there were 119 overlapping differential proteins between the two groups (Figure 2C, Table S1). GO (Gene Ontology) analysis of common differential proteins showed significant enrichments in 1231 biological processes (BPs) (Table S2), 154 cell components (CCs) (Table S3), and 143 molecular functions (MFs) (Table S4); there were also 150 KEGG (Kyoto Encyclopedia of Genes and Genomes) pathways to be enriched, and 29 were significantly enriched (*p* < 0.05; Figure 2D, Table S5).

### 2.3. Functional Categorization of Overlapping Differential Proteins

By analysis of GO terms, it was found that high levels of MT mainly affected BPs in myeloid leukocyte activation, the immune effector process, exocytotic regulation, and leukocyte activation in the immune response. The high levels of MT mainly affected CCs in the secretory vesicle, secretory granule, cytoplasm, intracellular vesicle, cytoplasmic vesicle, cytoplasmic part, and membrane-bounded vesicle of PBMCs; MT also affected the MFs in protein binding, enzyme binding, phosphoprotein binding, carboxylic acid binding, organic acid binding, and nuclear hormone receptor binding in these cells.

**Figure 3:**
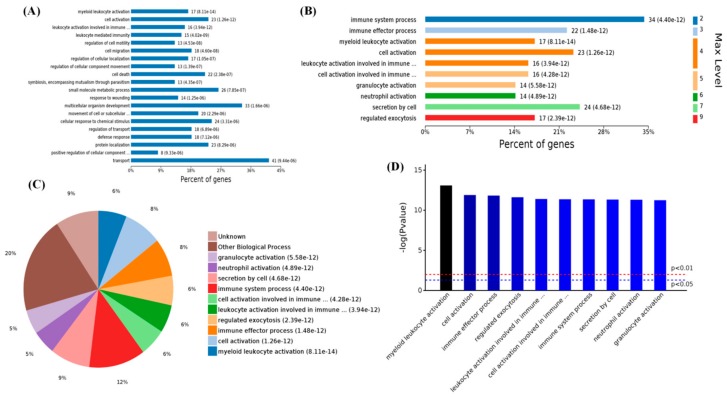
Enriched biological processes of 119 differential proteins in high-melatonin groups. (**A**) Levels of enriched biological processes; (**B**) significantly enriched biological processes (top 10); (**C**) expressed proteins of enriched biological processes; (**D**) significantly enriched biological processes with *p*-values.

Specifically, 1231 differential BPs were further analyzed. On the basis of the number of enriched genes (Figure 3A), the differential proteins in the high-level-of-MT groups were mainly enriched in transport (41 proteins), multicellular organism development (33 proteins), small-molecule metabolic processes (26 proteins), cellular responses to chemical stimuli (24 proteins), and cell activation (23 proteins). On the basis of the different levels of GO classification (Figure 3B), there were 34 proteins associated with the immune system process (level 2 GO), which could be further subdivided into the BPs of immune response (levels 3–9 GO), the pie chart of the BPs distribution were shown in (Figure 3C). On the basis of the *p*-values of these BPs (Figure 3D), these differential proteins were most likely involved in myeloid leukocyte activation and the immune effector process. This showed that MT mainly affects the LPS-mediated immune response in PBMCs and regulates the immune response of organisms.

**Figure 4:**
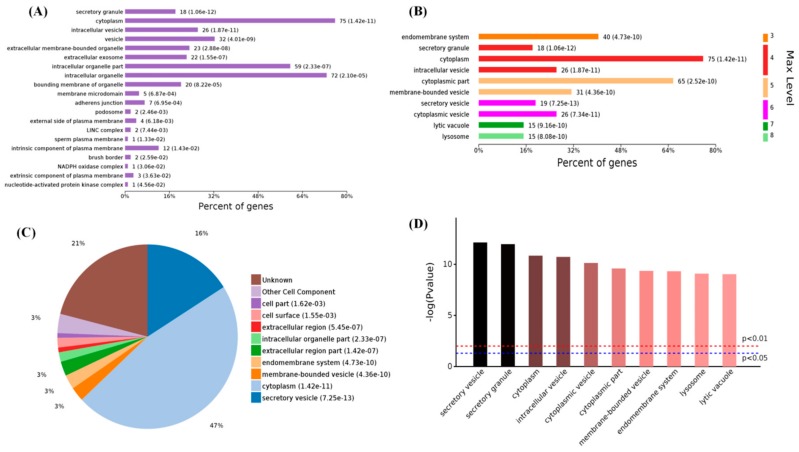
Enriched cell components of 119 differential proteins in high-melatonin groups. (**A**) Levels of enriched cell components; (**B**) significantly enriched cell components (top 10); (**C**) expressed proteins of enriched cell components; (**D**) significantly enriched cell components with *p*-values.

When the 154 differential CC terms were analyzed, the number of enriched proteins (Figure 4A) and differential proteins in the high-level-of-MT groups were mainly enriched in the cytoplasm (75 proteins), intracellular organelle (72 proteins), intracellular organelle part (59 proteins), vesicle (32 proteins), and intracellular vesicle (26 proteins). The different levels of GO classification (Figure 4B) showed that most of the differential proteins were associated with the immune system process (level 3 GO). For further subdivisions, the differential proteins were involved in the cytoplasm, intracellular vesicle (level 4), cytoplasmic part, membrane-bounded vesicle (level 5), secretory vesicle, cytoplasmic vesicle (level 6), lytic vacuole (level 7), and lysosome (level 8), the pie chart of the CC terms distribution were shown in (Figure 4C). When the *p*-values of the GO terms were evaluated (Figure 4D), these differential proteins were most likely involved in the secretory vesicle and secretory granule. The results indicated that MT mainly affects the intracellular membrane structure of PBMCs and influences the formation of lysosomes and their functions.

**Figure 5:**
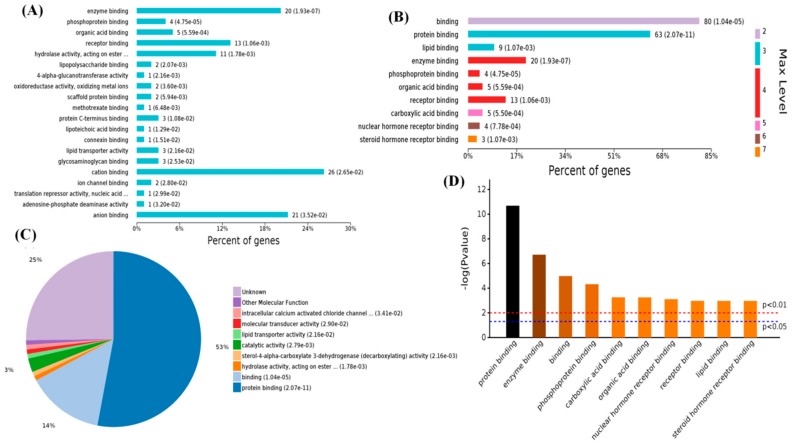
Enriched molecular functions of 119 differential proteins in high-melatonin groups. (**A**) Levels of enriched molecular functions. (**B**) Significantly enriched molecular functions (top 10). (**C**) Expressed proteins of enriched molecular functions. (**D**) Significantly enriched molecular functions with *p*-values.

When the 143 differential MF terms were analyzed, the number of enriched proteins (Figure 5A) and differential proteins were mainly enriched in cation binding (26 proteins), anion binding (21 proteins), and enzyme binding (20 proteins). The different levels of GO classification (Figure 5B) indicated that most of the differential genes were related to bindings (level 2 GO), including protein and lipid binding (level 3); enzyme binding, receptor binding, and so on (level 4); carboxylic acid binding (level 5 GO); nuclear hormone receptor binding (level 6 GO); and steroid hormone receptor binding (level 7 GO) and the pie chart of the MFs distribution were shown in (Figure 5C). The *p*-value of GO term analysis (Figure 5D) indicated that the differential proteins mainly participated in protein binding.

### 2.4. Melatonin Improved the Antioxidant Capacity in PBMCs

**Figure 6:**
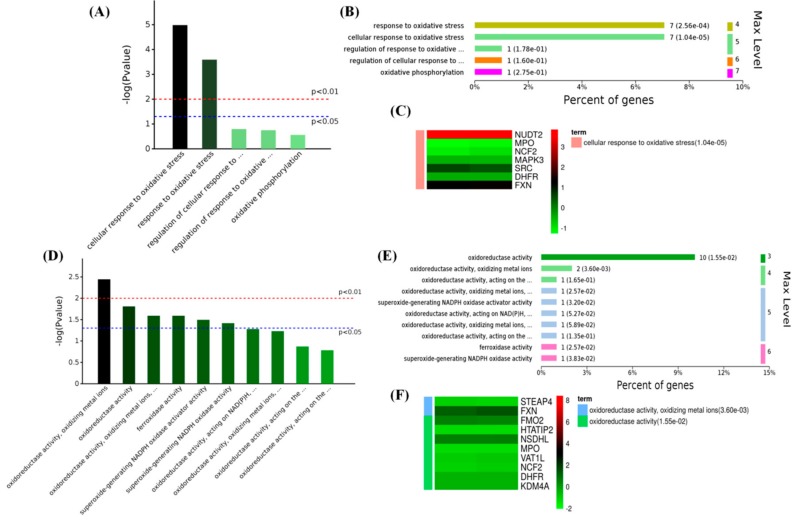
Enriched GO of 119 differential proteins in oxidative stress-related terms. (**A**) Oxidative stress-related biological process terms with *p*-values; (**B**) significantly enriched biological processes of oxidative stress (high-melatonin-level goats vs wild-type (WT) goats); (**C**) expression of oxidative stress-related proteins in biological process terms: red: upregulation; green: downregulation (high-melatonin-level goats vs WT goats); (**D**) oxidative stress-related molecular function terms; (**E**) significantly enriched molecular function of oxidative stress (high-melatonin-level goats vs WT goats); (**F**) expression of oxidative stress-related proteins in molecular function terms: red: upregulation; green: downregulation (high-melatonin-level goats vs WT goats).

By analysis of the oxidative stress-related proteins of GO terms, it was found that MT affected the BPs of cellular responses to oxidative stress (7 proteins) (Figure 6A,B) and oxidoreductase activity (10 proteins) (Figure 6D,E). The antioxidative-associated proteins, such as NUDT2 (Nudix hydrolase 2) and FXN (Frataxin), were significantly improved by MT, and the levels of pro-oxidation-associated proteins, such as NCF2 (Neutrophil cytosolic factor 2) and MPO (Myeloperoxidase), were significantly decreased (Figure 6C,F).

### 2.5. KEGG Pathway Analysis of Overlapping Differential Proteins

**Figure 7:**
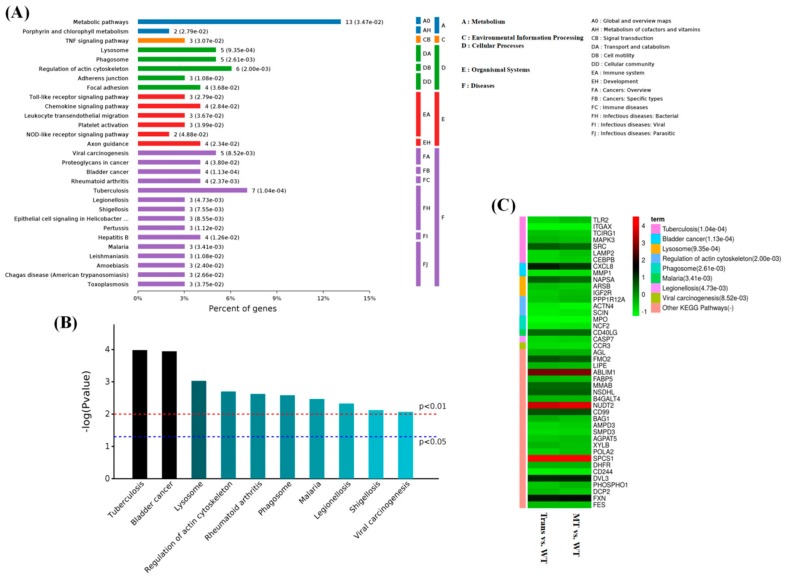
Enriched KEGG pathway of 119 differential proteins. (**A**) Classes of enriched KEGG pathway; (**B**) distributions of enriched KEGG pathway (top 10); (**C**) differential proteins in KEGG pathway: Red: Upregulation; green: Downregulation (high-melatonin-level goats vs wild-type (WT) goats).

**Figure 8:**
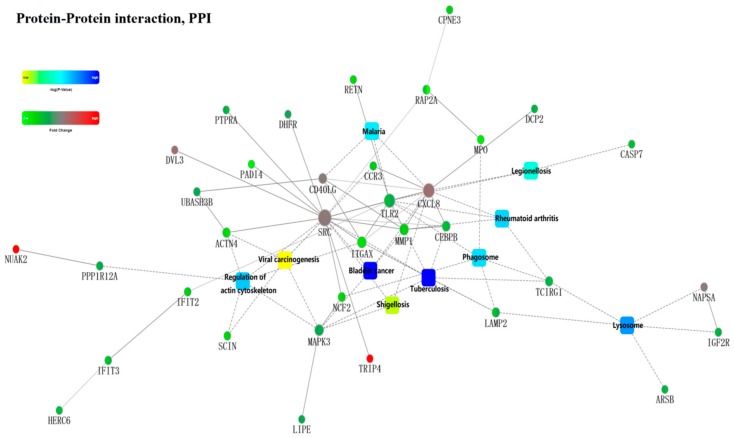
Protein–protein interaction graph.

By analysis of the KEGG pathway of 119 overlapping differential proteins, it was found that they were mainly involved in the regulation of intracellular metabolism pathways; intracellular focal adhesion; and the regulation of actin cytoskeleton, lysosome, and other cellular processes. They also affected the Toll-like receptor signaling pathway, the chemokine signaling pathway, and so on. In terms of diseases, MT was seemingly involved in the regulation of tumorigenesis, tuberculosis, viral carcinogenesis, bladder cancer, rheumatoid arthritis, and so forth (Figure 7A), and this was further confirmed by the analysis of the *p*-values for the KEGG pathways (Figure 7B). The differential proteins were mainly enriched with MPO, LAMP2, TLR2 (Toll like receptor 2), MAPK3 (Mitogen-activated protein kinase 3), caspase-7, MMP1 (Matrix metallopeptidase 1), NCF2, CD99 (CD99 Molecule), and so on. (Figure 7C). A protein–protein interaction network map is shown in Figure 8.

## 3. Discussion

MT is a pleiotropic molecule with numerous physiological and pharmacological effects. Its effects on the regulation of immunity and inflammation have been well documented [28,30,41,42,43]. Not only are MT receptors widely distributed in the immune system [44,45,46,47,48], but the system itself can also synthesize MT [15]. All these indicate a close relationship between MT and the immune system. In general, MT plays a check-and-balance role for immunoresponses in the majority of organisms. Under normal conditions, MT is an immunostimulator, preparing the body to enter a pre-activated state and to eliminate pathogens more effectively [27]. This immunostimulating effect is particularly important under immunosuppressive conditions, such as in Alzheimer′s disease [49,50]. In contrast, MT also exerts negative regulatory effects in situations of exacerbated immune response, such as in sepsis and other autoimmune disorders [24,51,52].

In the past, many studies have investigated the effects of MT on the exacerbated inflammatory response in different animal models [53,54,55]. In the majority of the studies, the MT was exogenously administered [56,57,58]. Thus, there has been no sufficient evidence to show the roles that endogenous MT plays in this respect. In the current study, transgenic goats with endogenous high levels of MT were used to test responses to the LPS-induced inflammatory response and the potential molecular mechanisms. It was found that the transgenic goats exhibited significantly higher serum levels of MT than the WT goats, and they also had different responses to LPS stimulation compared to the WT goats. After LPS induction, the WT goats exhibited excessive inflammatory responses with a fever over 40 °C and significantly increased granulocyte counts. These symptoms were not observed in the transgenic goats. The rescue study by MT injection into the WT goats with LPS stimulation confirmed that the protective effects of transgenic goats on LPS were attributed to the high levels of endogenously produced MT. This is the first study to show that a high level of endogenous MT per se suppresses the toxic effects of LPS in large animals [59,60].

To identify the potential molecular mechanisms, a proteogenomic analysis was conducted for the PBMCs isolated from the transgenic animals, as these cells are critical for inflammatory responses. It was found that 185 proteins were significantly differentially expressed (51 upregulated and 134 downregulated) in the PBMCs of transgenic goats with LPS treatment, and these numbers increased to 459 in MT-treated WT goats with LPS treatment. However, 119 overlapping differential proteins were identified in these two high-levels-of-MT groups. GO analysis showed that MT affected the body’s immune response by regulating the function of PBMCs. In BPs, MT changes the transport function of PBMCs and leads to modification of their phagocytosis of pathogens and antigen presentation [61,62]. In CCs, high levels of MT modify the intracellular organelles, intracellular membrane structure, and thus their lysosome functions, which directly affects their phagocytosis and autophagy [63]. These observations are supported by previous reports of MT effects on phagocytosis, autophagy, and mitophagy [64,65,66]. MF analysis indicated that MT mainly affected protein and enzyme bindings and their functions. The intracellular protease system is an important safeguard for PBMCs’ maintenance of the immune function [67]. The KEGG pathway analysis showed that MT negatively regulated several pathways associated with diseases including tuberculosis (dowregulation of TLR2, ITGAX (Integrin subunit alpha X), TCIRG1 (T cell immune regulator 1), MAPK3, and CEBPB (transcription factor CCAAT/enhancer-binding protein, beta); Figure S2), bladder cancer (downregulation of MMP1 and MAPK3; Figure S3), and rheumatoid arthritis (Figures S4–S6).

**Figure 9:**
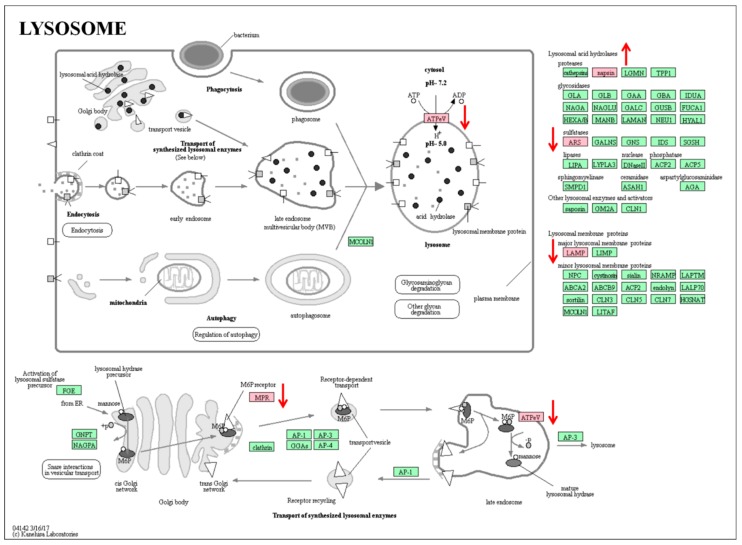
Overlapping differential proteins in KEGG pathway of lysosome.

With regard to the mechanisms of MT on the inhibition of an excessive inflammatory response, high levels of MT reduced the lysosome formation by downregulation of LAMP2, IGF2R (Insulin like growth factor 2 receptor), and ARSB (Arylsulfatase B) and lowered its function by reducing its ATP synthase-associated proteins in PBMCs (Figure 9). In addition, MT increased the antioxidant capacity of PBMCs, which further provided additional protection against excessive inflammation. For example, NCF2 and NADPH (DECR1, 2,4-dienoyl-CoA reductase 1) oxidase, as the sources of ROS production in the skeletal muscle of obese individuals [68], produced a burst of superoxide that was delivered to the lumen of the neutrophil phagosome and resulted in cell damage. High levels of MT significantly reduce the expression level of NCF2 protein in PBMCs and thus alleviate the damage of cells caused by excessive ROS. OCT-2 (POU class 2 homeobox 2) directly regulates the expression of anti-apoptotic gene *Bcl*-*2* to reduce apoptosis, and MT significantly increased the expression of OCT-2 protein and downregulated the expression of caspase-7 protein in PBMCs. The proteomic analysis also indicated that MT downregulated the expression of the transcription factor CEBPB, which regulates the cell differentiation, cell proliferation, survival, and metabolism of many cell types through the activation or repression of target genes [69,70,71,72]. Within the hematopoietic system, CEBPB is involved in the regulation of the development and function of macrophages [69,73]. CEBPB is important in the regulation of genes involved in immune and inflammatory responses as well as in regulatory regions of several acute-phase and cytokine genes [74,75,76]. In addition, CEBPB can bind the promoter and upstream element and stimulate the expression of the targeted gene [77,78,79].

In this study, the effects of endogenous MT on an excessive inflammatory response were first investigated in a transgenic MT-enriched animal model (goats). The results confirmed that endogenously produced high levels of MT exhibited significant anti-inflammatory effects in the animals. The proteogenomic analysis revealed the potential mechanisms. These involved but were not limited to MT suppressing the lysosome formation and functions by downregulation of the expressions of their associated genes in the PBMCs and enhancing the antioxidant capacity of these cells to reduce the cell apoptosis induced by the LPS. In addition, the proteogenemic analysis also uncovered previously unknown information that showed that high levels of MT may have protective effects on some human diseases, including tuberculosis, bladder cancer, and rheumatoid arthritis, as MT downregulated these diseases’ associated gene expressions. These observations warrant further investigations on this subject.

## 4. Materials and Methods

### 4.1. Animals

This study was conducted using adult Laoshan dairy goats (*Capra hircas*) fed with a live-weight maintenance ration at the experimental facilities of Qingdao Sanuels Industrial & Commercial Co., Ltd., Qingdao, China. All animal treatments and experiments were supervised and approved in accordance with the requirements of the Animal Welfare Committee (Permit No. XK662, 22 September 2015 to 22 September 2020) of China Agricultural University.

### 4.2. Melatonin-Enriched Goat Model

Transgenic goats with a higher MT level were produced by our team to overexpress *Aanat* using somatic cell nuclear transfer (SCNT) technology. Briefly, a pIRES2-EGFP-*Aanat* expression vector was constructed and was transferred into the female fetal fibroblast cells (FFCs) via electrotransfection, after which the nuclei of the transgenic FFCs were transferred to the eggs of the donor goats; the details of the generation of the transgenic goats are described in a previous article [80].

### 4.3. In Vivo Study

We divided the goats into five groups: WT, WT plus LPS, WT plus LPS and MT, transgenic, and transgenic plus LPS groups (*n* = 3 in each group). The LPS and MT treatment are described previously [80]. We collected PBMCs and serum in order to test MT levels and the blood physiological index.

### 4.4. Melatonin Assay

Blood was collected from the jugular vein of the transgenic and WT goats. Serum was obtained by centrifugation at 3000 rpm for 10 min. All procedures were performed under ice and the samples were kept in the dark. We detected MT by radioimmunoassay (BAR-3900, LDN, Nordhorn, Germany).

### 4.5. Detection of the Blood Physiological Index

We collected blood from the jugular vein of the goats (1–2 mL of blood from each goat) and stored this in an EDTANa2/K anticoagulant tube; the blood was measured using a blood cell analyzer (PE-6800, PROKRN, Shenzhen, China) within 2 h of collection.

### 4.6. PBMCs Isolation and Proteome Analysis

We isolated PBMCs from the blood of the goats using PBMC separation medium (Solarbio, P5290, Beijing, China). We performed our experiments at CapitalBio Technology (Beijing, China); the method of proteome analysis was carried out according to the method of Tao et al. described previously [80].

### 4.7. Statistics Analysis

The data were analyzed using univariate analysis of variance (ANOVA) followed by Duncan’s test using SPSS 18.0 statistical software (SPSS Inc., Chicago, IL, USA). The data were expressed as the mean values ± standard error of the mean (SEM); *p* < 0.05 was considered significant, and *p* < 0.01 was considered highly significant.

## Supplementary Materials

Supplementary materials can be found at http://www.mdpi.com/1422-0067/19/8/2406/s1.
