# Supplementary material for: Responses of Transgenic Melatonin-Enriched Goats on LPS Stimulation and the Proteogenomic Profiles of Their PBMCs
Source: Int J Mol Sci. 2018 Aug 15;19(8):2406. doi: 10.3390/ijms19082406 (PMC6121286; doi:10.3390/ijms19082406)
Supplement: Supplementary file 1 [file ijms-19-02406-s001.pdf]

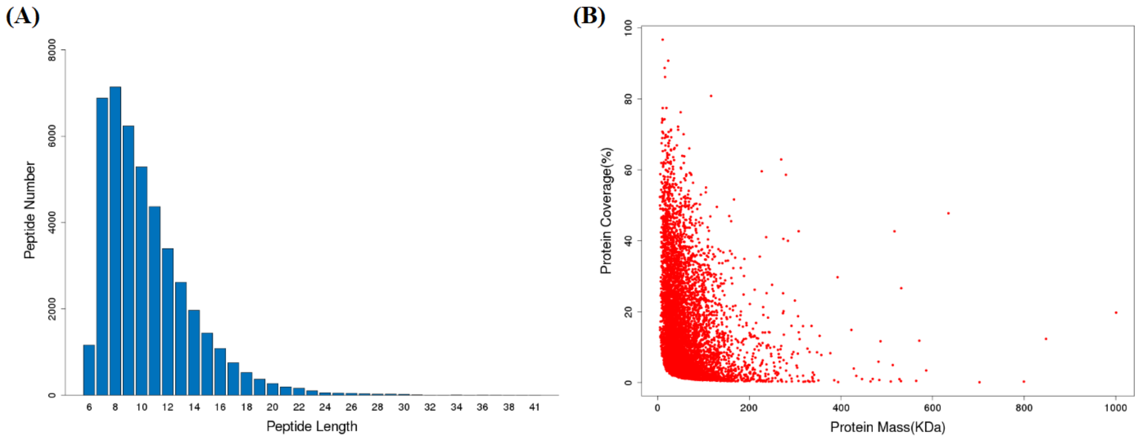

Figure S1. Statistics of peptide length distribution and protein molecular weight distribution. (**A**) Peptide length distribution profile, abscissa: peptide length; ordinate: peptide number. (**B**) Molecular weight and coverage profile, abscissa: protein mass (KDa), ordinate: protein coverage (%).

Table S1. 119 overlapping differential expression proteins identified by Group A (Trans vs WT) and Group B (WT + MT vs. WT).

ID	FoldChange	Log2FoldChange
RNF14	11.0144569242,9.65185976746	3.46132645855,3.27080695469
LOC106503742	1.36711707452,1.35251520209	0.451136794954,0.435644809843
AGL	0.741325859732,0.764207144826	-0.43182025697,-0.387964348895
SYTL1	0.570309185394,0.575287660282	-0.810183825961,-0.797644569635
LOC102191717	0.673446495311,0.680342219495	-0.570364765407,-0.555667474738
PDCD11	0.696408705186,0.761861987582	-0.521993857938,-0.392398419826
NAPSA	1.47121798042,1.30155924167	0.557011016798,0.380240978833
LOC102181771	0.535216627869,0.515842194263	-0.901805157212,-0.954998309034
RETN	0.477973765388,0.491581715782	-1.06499665991,-1.02449683874
MRPL47	1.30963253668,1.4302417578	0.389162069872,0.51625903043
TTYH3	0.709756752251,0.741155087849	-0.494603425834,-0.432152634597
FMO2	1.56952082785,1.46097454459	0.650324173894,0.546931041445
ATPAF1	0.551123609251,0.453705932296	-0.859552163653,-1.14017057155
LIPE	0.731676570213,0.709749876258	-0.450722033352,-0.494617402466
RFLNB	0.61005866975,0.658224630367	-0.712980100594,-0.60334808264
LOC102179937	2.92170640536,5.03664021198	1.54681121277,2.33246167701
ABLIM1	6.99091944746,7.63922482476	2.80548221169,2.93342625106
ABHD2	0.532411250139,0.578424431799	-0.909387038385,-0.78979960399
HTATIP2	0.416566041711,0.421329039851	-1.26338285861,-1.24698073867
OSBPL10	0.660476587578,0.689398163308	-0.598420672831,-0.5365906398
XAF1	0.661925546559,0.684518595996	-0.595259143188,-0.546838359852
CXCL8	2.35047820646,1.95206376759	1.23295430398,0.965000181837
TLR2	0.613864003867,0.717322245446	-0.704009020201,-0.479306722869
FABP5	0.741054544827,0.720366046099	-0.432348359839,-0.473197912365
ADGRG3	0.572529317381,0.591509222827	-0.804578524023,-0.757527431609
ATP8B4	0.536700817627,0.558106764952	-0.897810008927,-0.841386961125
TRIP4	252.242077731,242.833450991	7.97866514851,7.92382336036

MMAB	1.38657450602,1.38887545166	0.471525140054,0.473917230472
LOC102180291	0.600350561646,0.668484792258	-0.736122917737,-0.581033354888
LOC102181993	1.75003138454,1.83008625295	0.807380795151,0.871911645105
LOC102176495	0.569182895444,0.607132026081	-0.813035786904,-0.719917817845
NSDHL	1.49208048294,1.49049253305	0.577325356777,0.575789147849
ARSB	0.694904707932,0.624877339294	-0.525112939813,-0.678355072088
NUAK2	337.839707915,310.863123163	8.40019509472,8.28013567366
MYO18A	0.618736462211,0.5650789858	-0.692603040246,-0.823475555753
IFIT3	0.563017176253,0.627154520227	-0.828749158875,-0.673107152497
IFIT2	0.502259863654,0.562816758144	-0.993494103216,-0.829262808724
LOC102181148	15.5759461621,16.1731637825	3.96124789742,4.01553002026
LOC108636851	0.62467287998,0.63692015515	-0.678827197876,-0.650815568543
KPNA3	13.557529254,12.4300551867	3.76102237824,3.63576079654
B4GALT4	0.736430475991,0.732654473471	-0.44137876335,-0.448795124274
PRSS57	122.989498403,118.189775984	6.94239132446,6.88496143024
RCN1	1.48679647101,1.43341157618	0.572207169003,0.519452910828
NUDT2	12.330016712,14.4586140914	3.62410285005,3.85385736645
NHSL2	0.563396152038,0.524092245907	-0.827778384662,-0.932107330835
HERC6	0.63771049156,0.60139248515	-0.649026478396,-0.733621254419
LOC102191038	153.468098435,142.131398922	7.26179498209,7.15108149217
ITGAX	0.451394626547,0.461913790118	-1.14753885026,-1.11430447739
CD99	1.6050992784,1.65386820649	0.682662533501,0.725844273555
MPO	0.41667224507,0.433037881143	-1.26301509092,-1.20743486082
DOPEY2	0.697700532644,0.709645013551	-0.519320159851,-0.494830570639
LOC102190927	0.455725657124,0.501618826587	-1.13376249904,-0.995336598992
TCIRG1	0.683849321688,0.636987083125	-0.548249615976,-0.650663977195
VAT1L	0.505671037007,0.568230515419	-0.983728946367,-0.815451784899
NCF2	0.471160789312,0.521702954027	-1.08570861391,-0.938699492559
BAG1	0.767983538406,0.698671021338	-0.380852707528,-0.517314791799
CD40LG	1.34213119479,1.3380106947	0.424525703447,0.420089647466
AMPD3	0.584313373092,0.633396029426	-0.775185786483,-0.658820271325
NDUFAF7	1.38661399499,1.35569179208	0.471566226725,0.439029228195
CCR3	0.559760102557,0.534361467174	-0.837119433797,-0.90411211612
SMPD3	0.555837379709,0.586232560539	-0.847265236766,-0.770454994442
IGF2R	0.631779713189,0.709815660131	-0.662506482829,-0.494483691038
LOC102174471	0.554564264126,0.555160287968	-0.85057344214,-0.849023722938
SORL1	0.645887601242,0.657048232718	-0.630644969038,-0.605928814828
MAPK3	0.734051240055,0.698386730551	-0.446047321747,-0.517901947074
PPP1R12A	0.669198332044,0.734836604951	-0.579494245379,-0.444504600629
TMEM30A	0.741101252649,0.752865410889	-0.432257431283,-0.409536116309
CST7	0.522895490213,0.49822559511	-0.935405467379,-1.00512895651
SRC	1.54160277267,1.37408552323	0.624431071527,0.458471800444
RAP2A	0.567404163942,0.35961619801	-0.8175513569,-1.47547008999
ELL	0.685801080256,0.650065562713	-0.544137918116,-0.621342865622
ARL6IP6	0.698593255769,0.719124446104	-0.517475379889,-0.475686640994
LAMP2	0.576518833237,0.688644578107	-0.794560357481,-0.538168520816
SYNE1	0.607050093252,0.623621486422	-0.720112523539,-0.681257458993
NUDT18	0.708095120486,0.713356518672	-0.497984920122,-0.487304813212
TM6SF1	10.1741906244,10.8263798726	3.3468421249,3.43647900977
LRRFIP1	0.683724780598,0.617793181418	-0.548512380258,-0.694804146856
AGPAT5	0.669812098594,0.719429213018	-0.578171659685,-0.4750753524

TMSB15B	0.671485933086,0.726126885666	-0.57457091784,-0.461706423628
XYLB	0.73714333329,0.686654197363	-0.439982924521,-0.54234436171
STEAP4	0.475058888366,0.451005201499	-1.07382173368,-1.14878402253
GLCCI1	0.671881210603,0.640644600239	-0.573721909536,-0.642403856088
POLA2	0.607145712774,0.680115088643	-0.719885295262,-0.556149195928
SPCS1	19.7849562717,23.1431277436	4.30633197159,4.53251194962
LOC102175938	0.615900642519,0.624329315843	-0.699230461673,-0.679620884694
HDHD2	0.768426129638,0.704273528972	-0.380021517505,-0.505792236693
DHFR	0.748780667563,0.749836046554	-0.417384907791,-0.41535291352
TMEM254	0.763772302061,0.754152393937	-0.388785492816,-0.407072012027
ADGRE1	0.385177411048,0.487865881676	-1.37640499691,-1.03544350127
CD244	0.455832766086,0.472099752069	-1.13342346302,-1.0828363696
SPNS3	0.447324783094,0.479203770877	-1.16060540483,-1.06128883408
RNASE2	0.254940563955,0.243935224263	-1.97176715401,-2.03542999645
BIN3	0.6134189462,0.628795370057	-0.70505536818,-0.669337500097
GRAMD2B	0.700085307099,0.731607212229	-0.514397366215,-0.450858797554
CEBPB	0.591047586986,0.641031028288	-0.758653804172,-0.641533904543
UBASH3B	0.708422599646,0.702739415486	-0.497317858126,-0.508938275689
SUN2	0.657036297652,0.684183183625	-0.605955021149,-0.547545450002
DVL3	2.24591993679,2.33716354357	1.16730649904,1.22475869058
KDM4A	0.731385061583,0.725951458768	-0.451296934711,-0.462055010214
PHOSPHO1	0.653365247133,0.748105505035	-0.614038375836,-0.418686347693
LOC108636909	0.286784177357,0.348670280517	-1.80196266596,-1.52006469601
MEA1	0.702799037033,0.641107722194	-0.508815880304,-0.641361308686
DCP2	0.717712702748,0.634121660115	-0.478521639791,-0.657168438109
LOC102190765	0.544396646272,0.569066787392	-0.877269915668,-0.813330113384
MMP1	0.527535778294,0.533228426853	-0.922659151936,-0.907174401132
FXN	2.46628257883,2.72737021159	1.30233810863,1.4475105441
PADI4	0.400181291461,0.415086773795	-1.32127437229,-1.26851513183
LOC102169702	0.436989235635,0.51127956997	-1.19433035266,-0.967815715825
LOC102181699	0.52987146839,0.490161056976	-0.916285649183,-1.02867222745
CASP7	0.597006728158,0.610746415893	-0.744180904393,-0.711354602688
AOAH	0.715512915754,0.635699279361	-0.482950285551,-0.653583641918
NRM	0.646710512331,0.725904224495	-0.628808033188,-0.462148882701
ACTN4	0.498232935913,0.450659334789	-1.00510770015,-1.14989082044
CPNE3	0.518869734221,0.540708356955	-0.946555709257,-0.887077440623
PTPRA	0.723091056081,0.597969730517	-0.467750763223,-0.741855638454
LOC102171106	0.214028212172,0.385322803844	-2.22412711659,-1.37586052602
SCIN	0.485615216152,0.525367851482	-1.04211446752,-0.928600173682
POU2F2	9.83955549741,9.9662468258	3.29859314319,3.3170503045
FES	0.709120792192,0.683707635433	-0.495896696685,-0.548548557906

Table S2. All Enriched Biological Process.

GO_Name	GO_ID	Pvalue	Pvalue_adjusted	PAS_Value	PAS_Zscore
myeloid leukocyte activation	GO:0002274	8.11E-14	2.27E-10	-4.46,-4.23	-1.33,-1.26
cell activation	GO:0001775	1.26E-12	1.38E-09	-5.59,-5.34	-1.75,-1.68
immune effector process	GO:0002252	1.48E-12	1.38E-09	-5.32,-5.0	-1.65,-1.55
regulated exocytosis	GO:0045055	2.39E-12	1.52E-09	-4.4,-4.23	-1.31,-1.26

leukocyte activation involved in immune response	GO:0002366	3.94E-12	1.52E-09	-4.01,-3.82	-1.16,-1.1
cell activation involved in immune response	GO:0002263	4.28E-12	1.52E-09	-4.01,-3.82	-1.16,-1.1
immune system process	GO:0002376	4.40E-12	1.52E-09	-8.65,-8.08	-2.9,-2.71
secretion by cell	GO:0032940	4.68E-12	1.52E-09	-6.95,-6.82	-2.26,-2.23
neutrophil activation	GO:0042119	4.89E-12	1.52E-09	-3.76,-3.49	-1.07,-0.98
granulocyte activation	GO:0036230	5.58E-12	1.56E-09	-3.76,-3.49	-1.07,-0.98
leukocyte degranulation	GO:0043299	1.57E-11	3.99E-09	-3.54,-3.36	-0.98,-0.93
myeloid cell activation involved in immune response	GO:0002275	1.85E-11	4.32E-09	-3.54,-3.36	-0.98,-0.93
secretion	GO:0046903	2.32E-11	4.69E-09	-6.95,-6.82	-2.26,-2.23
myeloid leukocyte mediated immunity	GO:0002444	2.34E-11	4.69E-09	-3.54,-3.36	-0.98,-0.93
leukocyte activation	GO:0045321	2.73E-11	5.11E-09	-5.01,-4.69	-1.53,-1.43
neutrophil degranulation	GO:0043312	5.67E-11	9.34E-09	-3.39,-3.2	-0.93,-0.87
neutrophil activation involved in immune response	GO:0002283	5.67E-11	9.34E-09	-3.39,-3.2	-0.93,-0.87
immune response	GO:0006955	6.11E-11	9.52E-09	-7.27,-6.75	-2.38,-2.21
neutrophil mediated immunity	GO:0002446	8.82E-11	1.30E-08	-3.39,-3.2	-0.93,-0.87
exocytosis	GO:0006887	1.97E-10	2.76E-08	-4.66,-4.46	-1.4,-1.35
locomotion	GO:0040011	3.12E-10	4.16E-08	-7.7,-7.81	-2.54,-2.61
leukocyte mediated immunity	GO:0002443	4.02E-09	5.12E-07	-3.67,-3.49	-1.03,-0.98
regulation of localization	GO:0032879	5.95E-09	7.25E-07	-7.55,-7.64	-2.48,-2.54
developmental process	GO:0032502	2.04E-08	2.38E-06	-12.56,-12.55	-4.36,-4.39
single-multicellular organism process	GO:0044707	3.70E-08	4.15E-06	-10.61,-10.44	-3.63,-3.6
single-organism developmental process	GO:0044767	4.28E-08	4.44E-06	-12.43,-12.41	-4.31,-4.34
vesicle-mediated transport	GO:0016192	4.46E-08	4.44E-06	-6.23,-5.91	-1.99,-1.89
regulation of cell motility	GO:2000145	4.53E-08	4.44E-06	-3.0,-3.02	-0.78,-0.8
cell migration	GO:0016477	4.60E-08	4.44E-06	-4.39,-4.41	-1.3,-1.32
multicellular organismal process	GO:0032501	6.16E-08	5.75E-06	-10.88,-10.68	-3.73,-3.69
single-organism localization	GO:1902578	6.48E-08	5.86E-06	-11.93,-11.7	-4.12,-4.07
macromolecule localization	GO:0033036	7.98E-08	6.99E-06	-9.0,-8.99	-3.03,-3.05
regulation of cellular localization	GO:0060341	1.05E-07	8.94E-06	-5.28,-5.23	-1.64,-1.64
positive regulation of cellular process	GO:0048522	1.11E-07	9.11E-06	-11.52,-11.52	-3.97,-4.0
regulation of locomotion	GO:0040012	1.15E-07	9.17E-06	-3.0,-3.02	-0.78,-0.8
regulation of cellular component movement	GO:0051270	1.39E-07	1.06E-05	-3.0,-3.02	-0.78,-0.8
cell motility	GO:0048870	1.44E-07	1.06E-05	-4.39,-4.41	-1.3,-1.32
localization of cell	GO:0051674	1.44E-07	1.06E-05	-4.39,-4.41	-1.3,-1.32

response to stress	GO:0006950	1.71E-07	1.21E-05	-11.64,-11.68	-4.01,-4.06
regulation of cell migration	GO:0030334	1.73E-07	1.21E-05	-2.85,-2.86	-0.73,-0.74
anatomical structure development	GO:0048856	2.01E-07	1.37E-05	-11.28,-11.28	-3.88,-3.91
cell death	GO:0008219	2.38E-07	1.59E-05	-8.41,-8.2	-2.8,-2.75
localization	GO:0051179	2.95E-07	1.92E-05	-16.14,-15.87	-5.7,-5.64
response to chemical	GO:0042221	4.14E-07	2.64E-05	-12.36,-12.31	-4.28,-4.3
symbiosis, encompassing mutualism through parasitism	GO:0044403	4.35E-07	2.71E-05	-4.12,-3.91	-1.2,-1.14
regulation of biological quality	GO:0065008	5.45E-07	3.32E-05	-8.32,-8.11	-2.77,-2.72
single-organism transport	GO:0044765	7.55E-07	4.50E-05	-11.29,-10.91	-3.88,-3.77
small molecule metabolic process	GO:0044281	7.85E-07	4.58E-05	-5.28,-5.09	-1.64,-1.58
apoptotic process	GO:0006915	8.44E-07	4.82E-05	-8.08,-7.88	-2.68,-2.63
interspecies interaction between organisms	GO:0044419	8.61E-07	4.83E-05	-4.12,-3.91	-1.2,-1.14
regulation of cell death	GO:0010941	1.10E-06	6.06E-05	-6.79,-6.57	-2.2,-2.14
response to wounding	GO:0009611	1.25E-06	6.74E-05	-3.06,-2.87	-0.8,-0.74
regulation of developmental process	GO:0050793	1.35E-06	7.12E-05	-7.5,-7.47	-2.47,-2.48
regulation of multicellular organismal process	GO:0051239	1.46E-06	7.55E-05	-5.17,-5.17	-1.59,-1.61
multicellular organism development	GO:0007275	1.66E-06	8.37E-05	-7.78,-7.79	-2.57,-2.6
single-organism process	GO:0044699	1.67E-06	8.37E-05	-26.66,-26.28	-9.63,-9.56
programmed cell death	GO:0012501	1.76E-06	8.49E-05	-8.08,-7.88	-2.68,-2.63
regulation of secretion	GO:0051046	2.14E-06	1.02E-04	-3.79,-3.73	-1.08,-1.07
movement of cell or subcellular component	GO:0006928	2.29E-06	1.07E-04	-5.37,-5.51	-1.67,-1.74
regulation of protein oligomerization	GO:0032459	2.64E-06	1.21E-04	-0.89,-0.76	0.0,0.05
positive regulation of transport	GO:0051050	3.03E-06	1.37E-04	-3.4,-3.31	-0.93,-0.91
cellular response to chemical stimulus	GO:0070887	3.31E-06	1.46E-04	-9.62,-9.5	-3.26,-3.24
cellular localization	GO:0051641	3.33E-06	1.46E-04	-8.82,-8.8	-2.96,-2.98
cell differentiation	GO:0030154	3.89E-06	1.68E-04	-8.96,-9.12	-3.01,-3.1
positive regulation of secretion	GO:0051047	4.75E-06	2.01E-04	-1.97,-1.83	-0.4,-0.35
response to progesterone	GO:0032570	5.20E-06	2.17E-04	-1.0,-0.8	-0.03,0.03
establishment of localization	GO:0051234	5.26E-06	2.17E-04	-14.49,-14.25	-5.08,-5.03
positive regulation of cell migration	GO:0030335	5.89E-06	2.39E-04	-1.99,-1.79	-0.4,-0.34
single-organism metabolic process	GO:0044710	6.35E-06	2.54E-04	-9.51,-9.43	-3.22,-3.21
positive regulation of biological process	GO:0048518	6.48E-06	2.56E-04	-11.64,-11.64	-4.01,-4.05
regulation of transport	GO:0051049	6.89E-06	2.68E-04	-5.55,-5.49	-1.73,-1.73

defense response	GO:0006952	7.12E-06	2.73E-04	-4.73,-4.51	-1.43,-1.36
response to lipid	GO:0033993	7.33E-06	2.77E-04	-5.0,-4.66	-1.53,-1.42
negative regulation of cell death	GO:0060548	7.46E-06	2.77E-04	-5.12,-5.05	-1.58,-1.57
regulation of secretion by cell	GO:1903530	7.50E-06	2.77E-04	-3.48,-3.45	-0.96,-0.96
positive regulation of cell motility	GO:2000147	7.87E-06	2.83E-04	-1.99,-1.79	-0.4,-0.34
negative regulation of protein oligomerization	GO:0032460	7.89E-06	2.83E-04	-0.19,-0.14	0.27,0.28
protein localization	GO:0008104	8.29E-06	2.94E-04	-7.61,-7.63	-2.5,-2.54
multi-organism process	GO:0051704	8.42E-06	2.95E-04	-6.12,-5.75	-1.95,-1.83
response to external stimulus	GO:0009605	8.61E-06	2.97E-04	-8.03,-7.75	-2.66,-2.58
blood coagulation	GO:0007596	8.69E-06	2.97E-04	-2.11,-2.02	-0.45,-0.42
hemostasis	GO:0007599	9.26E-06	3.02E-04	-2.11,-2.02	-0.45,-0.42
positive regulation of cellular component movement	GO:0051272	9.33E-06	3.02E-04	-1.99,-1.79	-0.4,-0.34
response to stimulus	GO:0050896	9.41E-06	3.02E-04	-19.94,-19.76	-7.12,-7.1
transport	GO:0006810	9.44E-06	3.02E-04	-14.25,-13.8	-4.99,-4.86
regulation of apoptotic process	GO:0042981	9.44E-06	3.02E-04	-6.46,-6.25	-2.08,-2.02
regulation of cytoplasmic transport	GO:1903649	9.47E-06	3.02E-04	-1.99,-1.86	-0.4,-0.36
single-organism biosynthetic process	GO:0044711	9.74E-06	3.03E-04	-4.11,-3.99	-1.2,-1.17
coagulation	GO:0050817	9.75E-06	3.03E-04	-2.11,-2.02	-0.45,-0.42
regulation of programmed cell death	GO:0043067	1.01E-05	3.12E-04	-6.46,-6.25	-2.08,-2.02
cellular response to oxidative stress	GO:0034599	1.04E-05	3.17E-04	-1.67,-1.73	-0.29,-0.32
positive regulation of locomotion	GO:0040017	1.08E-05	3.27E-04	-1.99,-1.79	-0.4,-0.34
single-organism cellular process	GO:0044763	1.11E-05	3.32E-04	-25.26,-24.9	-9.11,-9.04
regulation of multicellular organismal development	GO:2000026	1.22E-05	3.61E-04	-3.82,-3.78	-1.09,-1.09
response to lipopolysaccharide	GO:0032496	1.32E-05	3.86E-04	-1.84,-1.57	-0.35,-0.25
system development	GO:0048731	1.33E-05	3.86E-04	-6.89,-6.88	-2.24,-2.26
peptide transport	GO:0015833	1.35E-05	3.87E-04	-6.35,-6.22	-2.03,-2.01
phosphate-containing compound metabolic process	GO:0006796	1.48E-05	4.16E-04	-8.09,-8.18	-2.69,-2.74
wound healing	GO:0042060	1.48E-05	4.16E-04	-2.32,-2.22	-0.53,-0.5
positive regulation of protein transport	GO:0051222	1.63E-05	4.48E-04	-2.21,-2.14	-0.49,-0.47
cellular component organization or biogenesis	GO:0071840	1.63E-05	4.48E-04	-15.11,-15.2	-5.31,-5.39
organophosphate metabolic process	GO:0019637	1.65E-05	4.49E-04	-3.27,-3.1	-0.89,-0.83

response to organic substance	GO:0010033	1.67E-05	4.49E-04	-8.33,-7.94	-2.77,-2.65
regulation of intracellular transport	GO:0032386	1.68E-05	4.49E-04	-2.14,-2.02	-0.46,-0.43
cytosolic transport	GO:0016482	1.73E-05	4.58E-04	-5.17,-5.01	-1.59,-1.55
amide transport	GO:0042886	1.91E-05	5.00E-04	-6.35,-6.22	-2.03,-2.01
response to molecule of bacterial origin	GO:0002237	1.94E-05	5.04E-04	-1.84,-1.57	-0.35,-0.25
phosphorus metabolic process	GO:0006793	1.96E-05	5.04E-04	-8.09,-8.18	-2.69,-2.74
interaction with host	GO:0051701	2.12E-05	5.39E-04	-0.99,-0.9	-0.03,-0.0
positive regulation of multicellular organismal process	GO:0051240	2.34E-05	5.91E-04	-3.1,-2.95	-0.82,-0.78
regulation of protein transport	GO:0051223	2.64E-05	6.59E-04	-3.64,-3.63	-1.02,-1.03
positive regulation of secretion by cell	GO:1903532	2.66E-05	6.59E-04	-1.66,-1.55	-0.28,-0.25
negative regulation of biological process	GO:0048519	2.70E-05	6.65E-04	-9.53,-9.46	-3.22,-3.23
positive regulation of establishment of protein localization	GO:1904951	2.82E-05	6.86E-04	-2.21,-2.14	-0.49,-0.47
regulation of vesicle-mediated transport	GO:0060627	3.13E-05	7.57E-04	-1.88,-1.85	-0.37,-0.36
organic substance transport	GO:0071702	3.17E-05	7.58E-04	-7.73,-7.53	-2.55,-2.5
negative regulation of cellular process	GO:0048523	3.30E-05	7.83E-04	-9.03,-9.02	-3.04,-3.06
cellular developmental process	GO:0048869	3.49E-05	8.20E-04	-8.96,-9.12	-3.01,-3.1
muscle cell migration	GO:0014812	3.51E-05	8.20E-04	-0.91,-0.83	-0.0,0.02
hematopoietic or lymphoid organ development	GO:0048534	3.62E-05	8.37E-04	-1.93,-1.73	-0.38,-0.32
platelet activation	GO:0030168	3.86E-05	8.86E-04	-1.14,-1.08	-0.09,-0.07
cellular response to reactive oxygen species	GO:0034614	4.17E-05	9.50E-04	-0.58,-0.57	0.12,0.12
negative regulation of protein complex assembly	GO:0031333	4.65E-05	1.04E-03	-1.1,-0.9	-0.07,-0.0
regulation of protein localization	GO:0032880	4.67E-05	1.04E-03	-3.99,-4.0	-1.15,-1.17
cellular catabolic process	GO:0044248	4.69E-05	1.04E-03	-4.34,-4.27	-1.28,-1.27
cellular component organization	GO:0016043	4.72E-05	1.04E-03	-14.7,-14.85	-5.16,-5.26
catabolic process	GO:0009056	4.83E-05	1.06E-03	-4.81,-4.73	-1.46,-1.44
cellular response to lipopolysaccharide	GO:0071222	4.90E-05	1.06E-03	-0.56,-0.43	0.13,0.17
positive regulation of cytokine production	GO:0001819	5.02E-05	1.08E-03	-1.4,-1.29	-0.18,-0.15
regulation of cell adhesion	GO:0030155	5.07E-05	1.08E-03	-2.03,-1.87	-0.42,-0.37

regulation of vacuolar transport	GO:1903335	5.15E-05	1.09E-03	-0.19,-0.14	0.27,0.28
regulation of establishment of protein localization	GO:0070201	5.27E-05	1.11E-03	-3.64,-3.63	-1.02,-1.03
response to oxygen-containing compound	GO:1901700	5.81E-05	1.21E-03	-3.46,-3.24	-0.95,-0.89
cellular response to molecule of bacterial origin	GO:0071219	6.16E-05	1.27E-03	-0.56,-0.43	0.13,0.17
anatomical structure homeostasis	GO:0060249	6.19E-05	1.27E-03	-1.24,-1.09	-0.12,-0.07
regulation of body fluid levels	GO:0050878	6.21E-05	1.27E-03	-2.11,-2.02	-0.45,-0.42
positive regulation of immune effector process	GO:0002699	6.80E-05	1.38E-03	-1.05,-1.04	-0.05,-0.06
peptide secretion	GO:0002790	7.11E-05	1.43E-03	-2.76,-2.73	-0.69,-0.69
regulation of protein binding	GO:0043393	7.14E-05	1.43E-03	-0.96,-0.88	-0.02,0.0
immune system development	GO:0002520	7.35E-05	1.46E-03	-1.93,-1.73	-0.38,-0.32
regulation of cellular component organization	GO:0051128	7.43E-05	1.47E-03	-4.62,-4.8	-1.39,-1.47
vesicle-mediated transport between endosomal compartments	GO:0098927	7.90E-05	1.55E-03	-0.19,-0.14	0.27,0.28
response to organic cyclic compound	GO:0014070	8.02E-05	1.56E-03	-4.4,-4.2	-1.31,-1.25
telomere maintenance via telomere lengthening	GO:0010833	9.05E-05	1.74E-03	-0.68,-0.66	0.08,0.09
negative regulation of cellular component organization	GO:0051129	9.08E-05	1.74E-03	-2.15,-2.02	-0.47,-0.43
lipid metabolic process	GO:0006629	9.84E-05	1.87E-03	-2.74,-2.64	-0.69,-0.66
negative regulation of apoptotic process	GO:0043066	9.94E-05	1.87E-03	-4.8,-4.73	-1.45,-1.45
single-organism intracellular transport	GO:1902582	9.96E-05	1.87E-03	-5.68,-5.7	-1.78,-1.81
cellular response to biotic stimulus	GO:0071216	1.01E-04	1.89E-03	-0.56,-0.43	0.13,0.17
negative regulation of programmed cell death	GO:0043069	1.07E-04	1.99E-03	-4.8,-4.73	-1.45,-1.45
organelle organization	GO:0006996	1.12E-04	2.07E-03	-10.04,-10.13	-3.41,-3.48
response to bacterium	GO:0009617	1.14E-04	2.08E-03	-2.43,-2.18	-0.57,-0.49
animal organ development	GO:0048513	1.15E-04	2.08E-03	-5.26,-4.92	-1.63,-1.52
response to endogenous stimulus	GO:0009719	1.17E-04	2.12E-03	-5.4,-5.22	-1.68,-1.63
establishment of protein localization to organelle	GO:0072594	1.21E-04	2.18E-03	-4.36,-4.22	-1.29,-1.25
regulation of protein secretion	GO:0050708	1.29E-04	2.30E-03	-1.61,-1.63	-0.26,-0.28
intracellular transport	GO:0046907	1.30E-04	2.31E-03	-6.47,-6.37	-2.08,-2.06
leukocyte migration	GO:0050900	1.34E-04	2.36E-03	-1.87,-1.66	-0.36,-0.29

hemopoiesis	GO:0030097	1.36E-04	2.38E-03	-1.8,-1.57	-0.33,-0.26
positive regulation of cytokine secretion	GO:0050715	1.55E-04	2.70E-03	-0.88,-0.76	0.01,0.05
multi-organism cellular process	GO:0044764	1.64E-04	2.84E-03	-3.04,-2.93	-0.8,-0.77
single-organism cellular localization	GO:1902580	1.66E-04	2.85E-03	-5.27,-5.5	-1.63,-1.74
establishment of protein localization	GO:0045184	1.72E-04	2.94E-03	-6.34,-6.43	-2.03,-2.09
vacuolar transport	GO:0007034	1.77E-04	3.01E-03	-0.91,-0.84	-0.0,0.02
response to hormone	GO:0009725	1.79E-04	3.01E-03	-4.45,-4.39	-1.33,-1.32
regulation of production of molecular mediator of immune response	GO:0002700	1.81E-04	3.03E-03	-0.82,-0.75	0.03,0.05
regulation of cellular component biogenesis	GO:0044087	1.89E-04	3.10E-03	-2.21,-1.92	-0.49,-0.39
myeloid cell differentiation	GO:0030099	1.89E-04	3.10E-03	-1.24,-1.07	-0.12,-0.07
alcohol metabolic process	GO:0006066	1.89E-04	3.10E-03	-1.41,-1.33	-0.19,-0.17
establishment of localization in cell	GO:0051649	1.90E-04	3.10E-03	-7.17,-7.01	-2.34,-2.3
neutral lipid catabolic process	GO:0046461	1.91E-04	3.10E-03	-0.54,-0.53	0.14,0.14
acylglycerol catabolic process	GO:0046464	1.91E-04	3.10E-03	-0.54,-0.53	0.14,0.14
response to other organism	GO:0051707	1.94E-04	3.13E-03	-2.98,-2.63	-0.78,-0.66
response to external biotic stimulus	GO:0043207	1.98E-04	3.18E-03	-2.98,-2.63	-0.78,-0.66
response to insulin	GO:0032868	2.00E-04	3.18E-03	-1.16,-1.17	-0.09,-0.1
caveolin-mediated endocytosis	GO:0072584	2.07E-04	3.26E-03	-0.05,0.02	0.32,0.34
protein catabolic process in the vacuole	GO:0007039	2.07E-04	3.26E-03	-0.4,-0.36	0.19,0.2
cell surface receptor signaling pathway	GO:0007166	2.21E-04	3.47E-03	-6.02,-5.68	-1.91,-1.8
protein targeting	GO:0006605	2.26E-04	3.51E-03	-4.36,-4.22	-1.29,-1.25
protein localization to organelle	GO:0033365	2.28E-04	3.53E-03	-4.55,-4.36	-1.36,-1.31
viral process	GO:0016032	2.35E-04	3.61E-03	-3.04,-2.93	-0.8,-0.77
regulation of cell proliferation	GO:0042127	2.38E-04	3.65E-03	-3.31,-3.08	-0.9,-0.82
response to interleukin-1	GO:0070555	2.48E-04	3.76E-03	-0.2,-0.08	0.27,0.31
positive regulation of protein secretion	GO:0050714	2.48E-04	3.76E-03	-1.08,-1.01	-0.07,-0.05
response to oxidative stress	GO:0006979	2.56E-04	3.86E-03	-1.67,-1.73	-0.29,-0.32
protein transport	GO:0015031	2.68E-04	3.99E-03	-6.09,-5.99	-1.94,-1.92
nitrogen compound transport	GO:0071705	2.68E-04	3.99E-03	-6.35,-6.22	-2.03,-2.01
response to reactive oxygen species	GO:0000302	2.91E-04	4.31E-03	-0.58,-0.57	0.12,0.12
response to biotic stimulus	GO:0009607	2.94E-04	4.32E-03	-2.98,-2.63	-0.78,-0.66

regulation of telomere maintenance via telomerase	GO:0032210	2.95E-04	4.32E-03	-0.19,-0.14	0.27,0.28
cellular response to interferon-alpha	GO:0035457	3.03E-04	4.40E-03	-0.55,-0.45	0.13,0.17
sequestering of actin monomers	GO:0042989	3.03E-04	4.40E-03	-0.49,-0.42	0.16,0.18
cellular defense response	GO:0006968	3.25E-04	4.70E-03	-0.74,-0.75	0.06,0.05
cellular protein localization	GO:0034613	3.31E-04	4.76E-03	-6.09,-6.03	-1.94,-1.94
regulation of smooth muscle cell migration	GO:0014910	3.41E-04	4.88E-03	-0.19,-0.14	0.27,0.28
cellular macromolecule localization	GO:0070727	3.56E-04	5.04E-03	-6.09,-6.03	-1.94,-1.94
negative regulation of protein homooligomerization	GO:0032463	3.58E-04	5.04E-03	0.05,0.02	0.36,0.34
regulation of metallopeptidase activity	GO:1905048	3.58E-04	5.04E-03	-0.32,-0.34	0.22,0.21
biological regulation	GO:0065007	3.65E-04	5.11E-03	-24.84,-24.43	-8.95,-8.87
protein complex subunit organization	GO:0071822	3.71E-04	5.17E-03	-4.74,-4.71	-1.43,-1.44
regulation of cell differentiation	GO:0045595	3.75E-04	5.20E-03	-5.06,-5.15	-1.55,-1.6
maintenance of protein location in cell	GO:0032507	4.03E-04	5.56E-03	-0.89,-0.81	0.01,0.03
regulation of telomere maintenance via telomere lengthening	GO:1904356	4.10E-04	5.64E-03	-0.19,-0.14	0.27,0.28
cellular lipid metabolic process	GO:0044255	4.16E-04	5.69E-03	-2.23,-2.09	-0.5,-0.45
cellular response to oxygen-containing compound	GO:1901701	4.19E-04	5.70E-03	-2.34,-2.27	-0.53,-0.52
cell development	GO:0048468	4.29E-04	5.80E-03	-3.89,-4.11	-1.11,-1.21
regulation of protein complex assembly	GO:0043254	4.44E-04	5.99E-03	-1.53,-1.34	-0.23,-0.17
glycerolipid catabolic process	GO:0046503	4.48E-04	6.01E-03	-0.54,-0.53	0.14,0.14
maintenance of protein location	GO:0045185	4.96E-04	6.62E-03	-0.89,-0.81	0.01,0.03
smooth muscle cell migration	GO:0014909	5.08E-04	6.75E-03	-0.19,-0.14	0.27,0.28
acylglycerol metabolic process	GO:0006639	5.54E-04	7.33E-03	-0.71,-0.67	0.07,0.08
regulation of cell activation	GO:0050865	5.60E-04	7.37E-03	-1.39,-1.35	-0.18,-0.17
neutral lipid metabolic process	GO:0006638	5.67E-04	7.42E-03	-0.71,-0.67	0.07,0.08
telomere maintenance via telomerase	GO:0007004	5.74E-04	7.47E-03	-0.19,-0.14	0.27,0.28
nuclear import	GO:0051170	5.76E-04	7.47E-03	-2.08,-1.94	-0.44,-0.39
organic substance catabolic process	GO:1901575	5.85E-04	7.55E-03	-3.69,-3.66	-1.04,-1.04

response to steroid hormone	GO:0048545	6.18E-04	7.94E-03	-3.69,-3.51	-1.04,-0.99
regulation of early endosome to late endosome transport	GO:2000641	6.21E-04	7.94E-03	-0.05,0.02	0.32,0.34
maintenance of location in cell	GO:0051651	6.30E-04	8.03E-03	-0.89,-0.81	0.01,0.03
regulation of cytokine production	GO:0001817	6.63E-04	8.41E-03	-1.4,-1.29	-0.18,-0.15
superoxide metabolic process	GO:0006801	6.69E-04	8.45E-03	-0.83,-0.77	0.03,0.05
negative regulation of growth of symbiont in host	GO:0044130	6.97E-04	8.76E-03	-0.59,-0.51	0.12,0.14
cellular response to organic substance	GO:0071310	7.18E-04	8.98E-03	-7.01,-6.67	-2.28,-2.18
small molecule biosynthetic process	GO:0044283	7.23E-04	9.01E-03	-1.88,-1.78	-0.36,-0.34
protein secretion	GO:0009306	7.29E-04	9.04E-03	-1.61,-1.63	-0.26,-0.28
negative regulation of growth of symbiont involved in interaction with host	GO:0044146	7.78E-04	9.48E-03	-0.59,-0.51	0.12,0.14
cytokine secretion involved in immune response	GO:0002374	7.78E-04	9.48E-03	-0.35,-0.3	0.21,0.22
regulation of growth of symbiont in host	GO:0044126	7.78E-04	9.48E-03	-0.59,-0.51	0.12,0.14
entry of bacterium into host cell	GO:0035635	7.78E-04	9.48E-03	0.56,0.43	0.55,0.5
regulation of cytokine secretion	GO:0050707	8.04E-04	9.75E-03	-0.88,-0.76	0.01,0.05
response to nutrient levels	GO:0031667	8.23E-04	9.94E-03	-3.89,-3.77	-1.12,-1.09
regulation of telomere maintenance	GO:0032204	8.31E-04	9.99E-03	-0.19,-0.14	0.27,0.28
telomere maintenance	GO:0000723	8.36E-04	1.00E-02	-0.68,-0.66	0.08,0.09
single-organism catabolic process	GO:0044712	8.48E-04	1.00E-02	-2.65,-2.61	-0.65,-0.65
cellular response to lipid	GO:0071396	8.49E-04	1.00E-02	-2.96,-2.81	-0.77,-0.72
organonitrogen compound metabolic process	GO:1901564	8.60E-04	1.00E-02	-14.27,-14.2	-5.0,-5.01
protein targeting to lysosome	GO:0006622	8.63E-04	1.00E-02	-0.43,-0.34	0.18,0.21
purine nucleoside catabolic process	GO:0006152	8.63E-04	1.00E-02	-0.38,-0.35	0.2,0.21
purine ribonucleoside catabolic process	GO:0046130	8.63E-04	1.00E-02	-0.38,-0.35	0.2,0.21
modulation of growth of symbiont involved in interaction with host	GO:0044144	8.63E-04	1.00E-02	-0.59,-0.51	0.12,0.14
telomere organization	GO:0032200	8.86E-04	1.03E-02	-0.68,-0.66	0.08,0.09

positive regulation of production of molecular mediator of immune response	GO:0002702	8.89E-04	1.03E-02	-0.69,-0.63	0.08,0.1
RNA-dependent DNA biosynthetic process	GO:0006278	9.20E-04	1.06E-02	-0.19,-0.14	0.27,0.28
negative regulation of protein binding	GO:0032091	9.51E-04	1.08E-02	-0.62,-0.59	0.11,0.11
negative regulation of telomere maintenance via telomerase	GO:0032211	9.53E-04	1.08E-02	-0.04,0.06	0.32,0.36
alcohol biosynthetic process	GO:0046165	9.55E-04	1.08E-02	-0.83,-0.77	0.03,0.05
regulation of binding	GO:0051098	9.70E-04	1.10E-02	-0.96,-0.88	-0.02,0.0
homeostatic process	GO:0042592	9.76E-04	1.10E-02	-2.93,-2.82	-0.76,-0.73
lysosomal transport	GO:0007041	9.82E-04	1.10E-02	-0.59,-0.55	0.12,0.13
response to drug	GO:0042493	9.90E-04	1.11E-02	-1.5,-1.51	-0.22,-0.23
cellular component biogenesis	GO:0044085	1.00E-03	1.11E-02	-6.38,-6.45	-2.05,-2.09
response to extracellular stimulus	GO:0009991	1.03E-03	1.14E-02	-3.89,-3.77	-1.12,-1.09
defense response to other organism	GO:0098542	1.03E-03	1.14E-02	-2.1,-1.92	-0.44,-0.39
regulation of myeloid cell differentiation	GO:0045637	1.05E-03	1.14E-02	-0.84,-0.79	0.02,0.04
positive regulation of interleukin-4 production	GO:0032753	1.05E-03	1.14E-02	0.1,0.07	0.38,0.36
response to interferon-alpha	GO:0035455	1.05E-03	1.14E-02	-0.55,-0.45	0.13,0.17
cellular response to organonitrogen compound	GO:0071417	1.09E-03	1.18E-02	-1.29,-1.26	-0.14,-0.14
actin cytoskeleton organization	GO:0030036	1.11E-03	1.20E-02	-4.32,-4.45	-1.28,-1.34
positive regulation of developmental process	GO:0051094	1.11E-03	1.20E-02	-2.33,-2.13	-0.53,-0.47
response to cytokine	GO:0034097	1.12E-03	1.20E-02	-1.82,-1.65	-0.34,-0.29
cell adhesion	GO:0007155	1.13E-03	1.21E-02	-3.25,-3.01	-0.88,-0.8
growth of symbiont in host	GO:0044117	1.14E-03	1.22E-02	-0.59,-0.51	0.12,0.14
organophosphate biosynthetic process	GO:0090407	1.15E-03	1.22E-02	-1.77,-1.69	-0.32,-0.3
ethanolamine-containing compound metabolic process	GO:0042439	1.15E-03	1.22E-02	-0.57,-0.5	0.13,0.15
biological adhesion	GO:0022610	1.16E-03	1.22E-02	-3.25,-3.01	-0.88,-0.8
cytokine production	GO:0001816	1.19E-03	1.25E-02	-1.4,-1.29	-0.18,-0.15
response to ketone	GO:1901654	1.21E-03	1.26E-02	-1.0,-0.8	-0.03,0.03
positive regulation of apoptotic process	GO:0043065	1.23E-03	1.27E-02	-1.68,-1.44	-0.29,-0.21
taxis	GO:0042330	1.23E-03	1.27E-02	-2.67,-2.74	-0.66,-0.7
chemotaxis	GO:0006935	1.23E-03	1.27E-02	-2.67,-2.74	-0.66,-0.7

positive regulation of protein autophosphorylation	GO:0031954	1.25E-03	1.27E-02	0.06,0.31	0.36,0.45
regulation of protein homooligomerization	GO:0032462	1.25E-03	1.27E-02	0.05,0.02	0.36,0.34
growth of symbiont involved in interaction with host	GO:0044116	1.25E-03	1.27E-02	-0.59,-0.51	0.12,0.14
growth involved in symbiotic interaction	GO:0044110	1.25E-03	1.27E-02	-0.59,-0.51	0.12,0.14
organic hydroxy compound metabolic process	GO:1901615	1.25E-03	1.27E-02	-1.41,-1.33	-0.19,-0.17
glycerolipid metabolic process	GO:0046486	1.28E-03	1.29E-02	-1.36,-1.23	-0.17,-0.13
positive regulation of programmed cell death	GO:0043068	1.28E-03	1.29E-02	-1.68,-1.44	-0.29,-0.21
cytokine secretion	GO:0050663	1.31E-03	1.32E-02	-0.88,-0.76	0.01,0.05
lamellipodium organization	GO:0097581	1.34E-03	1.33E-02	0.21,0.2	0.42,0.41
response to organonitrogen compound	GO:0010243	1.34E-03	1.33E-02	-1.74,-1.7	-0.31,-0.3
lipid transport	GO:0006869	1.39E-03	1.38E-02	-1.44,-1.35	-0.2,-0.17
regulation of homotypic cell-cell adhesion	GO:0034110	1.42E-03	1.40E-02	-0.32,-0.26	0.22,0.24
phospholipid metabolic process	GO:0006644	1.47E-03	1.45E-02	-1.4,-1.21	-0.18,-0.12
myeloid leukocyte differentiation	GO:0002573	1.48E-03	1.45E-02	-0.78,-0.63	0.05,0.1
response to acid chemical	GO:0001101	1.50E-03	1.47E-02	-0.95,-0.75	-0.02,0.05
regulation of immune effector process	GO:0002697	1.58E-03	1.54E-02	-1.17,-1.17	-0.1,-0.1
osteoclast differentiation	GO:0030316	1.62E-03	1.58E-02	-0.19,-0.14	0.27,0.28
response to inorganic substance	GO:0010035	1.65E-03	1.60E-02	-1.51,-1.49	-0.22,-0.22
protein complex assembly	GO:0006461	1.67E-03	1.62E-02	-3.76,-3.64	-1.07,-1.04
protein complex biogenesis	GO:0070271	1.68E-03	1.62E-02	-3.76,-3.64	-1.07,-1.04
nucleocytoplasmic transport	GO:0006913	1.69E-03	1.62E-02	-2.25,-2.07	-0.5,-0.44
cell communication	GO:0007154	1.70E-03	1.62E-02	-16.03,-15.7	-5.66,-5.58
negative regulation of telomere maintenance via telomere lengthening	GO:1904357	1.70E-03	1.62E-02	-0.04,0.06	0.32,0.36
insulin receptor signaling pathway	GO:0008286	1.76E-03	1.67E-02	-0.63,-0.71	0.1,0.07
positive regulation of cell death	GO:0010942	1.77E-03	1.68E-02	-1.68,-1.44	-0.29,-0.21
cellular component assembly	GO:0022607	1.81E-03	1.70E-02	-5.97,-6.1	-1.89,-1.96
regeneration	GO:0031099	1.84E-03	1.73E-02	-0.77,-0.67	0.05,0.08

positive regulation of cellular biosynthetic process	GO:0031328	1.85E-03	1.73E-02	-5.59,-5.48	-1.75,-1.73
cellular response to interleukin-1	GO:0071347	1.85E-03	1.73E-02	-0.37,-0.29	0.2,0.23
endocytosis	GO:0006897	1.86E-03	1.73E-02	-2.13,-1.94	-0.46,-0.39
regulation of immune system process	GO:0002682	1.87E-03	1.73E-02	-2.63,-2.44	-0.64,-0.58
positive regulation of cellular protein localization	GO:1903829	1.88E-03	1.73E-02	-1.53,-1.41	-0.23,-0.2
single organism signaling	GO:0044700	1.88E-03	1.73E-02	-15.79,-15.54	-5.57,-5.52
actin filament-based process	GO:0030029	1.94E-03	1.73E-02	-4.32,-4.45	-1.28,-1.34
protein localization to lysosome	GO:0061462	1.95E-03	1.73E-02	-0.43,-0.34	0.18,0.21
positive regulation of interleukin-10 production	GO:0032733	1.95E-03	1.73E-02	0.08,0.02	0.37,0.34
establishment of epithelial cell polarity	GO:0090162	1.95E-03	1.73E-02	-0.45,-0.69	0.17,0.07
signaling	GO:0023052	1.99E-03	1.73E-02	-15.79,-15.54	-5.57,-5.52
regulation of DNA biosynthetic process	GO:2000278	2.00E-03	1.73E-02	-0.19,-0.14	0.27,0.28
response to peptide hormone	GO:0043434	2.05E-03	1.73E-02	-1.16,-1.17	-0.09,-0.1
protein import	GO:0017038	2.06E-03	1.73E-02	-1.93,-1.72	-0.38,-0.31
transmembrane receptor protein tyrosine kinase signaling pathway	GO:0007169	2.07E-03	1.73E-02	-1.74,-1.73	-0.31,-0.32
ribonucleoside catabolic process	GO:0042454	2.08E-03	1.73E-02	-0.38,-0.35	0.2,0.21
positive regulation of biosynthetic process	GO:0009891	2.09E-03	1.73E-02	-5.59,-5.48	-1.75,-1.73
cellular response to endogenous stimulus	GO:0071495	2.12E-03	1.73E-02	-4.67,-4.46	-1.41,-1.34
generation of neurons	GO:0048699	2.12E-03	1.73E-02	-2.75,-3.0	-0.69,-0.79
cell proliferation	GO:0008283	2.15E-03	1.73E-02	-3.31,-3.08	-0.9,-0.82
lipid localization	GO:0010876	2.16E-03	1.73E-02	-1.44,-1.35	-0.2,-0.17
xylulose catabolic process	GO:0005998	2.17E-03	1.73E-02	0.0,0.0	0.34,0.34
negative regulation of apolipoprotein binding	GO:2000657	2.17E-03	1.73E-02	0.0,0.0	0.34,0.34
colon epithelial cell migration	GO:0061580	2.17E-03	1.73E-02	0.0,0.0	0.34,0.34
induction by symbiont of defense-related host nitric oxide production	GO:0052063	2.17E-03	1.73E-02	0.0,0.0	0.34,0.34
response to gold nanoparticle	GO:1990268	2.17E-03	1.73E-02	0.0,0.0	0.34,0.34
induction by organism of defense-related nitric oxide production in other organism involved in symbiotic interaction	GO:0052263	2.17E-03	1.73E-02	0.0,0.0	0.34,0.34

regulation of apolipoprotein binding	GO:2000656	2.17E-03	1.73E-02	0.0,0.0	0.34,0.34
positive regulation of choline O-acetyltransferase activity	GO:1902771	2.17E-03	1.73E-02	0.0,0.0	0.34,0.34
regulation of choline O-acetyltransferase activity	GO:1902769	2.17E-03	1.73E-02	0.0,0.0	0.34,0.34
deoxyribonucleoside diphosphate catabolic process	GO:0009192	2.17E-03	1.73E-02	0.0,0.0	0.34,0.34
positive regulation of myosin-light-chain-phosphatase activity	GO:0035508	2.17E-03	1.73E-02	0.0,0.0	0.34,0.34
positive regulation by organism of immune response of other organism involved in symbiotic interaction	GO:0052555	2.17E-03	1.73E-02	0.0,0.0	0.34,0.34
negative regulation of tau-protein kinase activity	GO:1902948	2.17E-03	1.73E-02	0.0,0.0	0.34,0.34
positive regulation of progesterone secretion	GO:2000872	2.17E-03	1.73E-02	0.0,0.0	0.34,0.34
dGDP catabolic process	GO:0046067	2.17E-03	1.73E-02	0.0,0.0	0.34,0.34
modulation by symbiont of defense-related host nitric oxide production	GO:0052163	2.17E-03	1.73E-02	0.0,0.0	0.34,0.34
induction by symbiont of host immune response	GO:0052559	2.17E-03	1.73E-02	0.0,0.0	0.34,0.34
induction by organism of immune response of other organism involved in symbiotic interaction	GO:0052558	2.17E-03	1.73E-02	0.0,0.0	0.34,0.34
regulation of ferrochelatase activity	GO:0010722	2.17E-03	1.73E-02	0.0,0.0	0.34,0.34
negative regulation of neurofibrillary tangle assembly	GO:1902997	2.17E-03	1.73E-02	0.0,0.0	0.34,0.34
positive regulation by symbiont of defense-related host nitric oxide production	GO:0052347	2.17E-03	1.73E-02	0.0,0.0	0.34,0.34
negative regulation of aspartic-type endopeptidase activity involved in amyloid precursor protein catabolic process	GO:1902960	2.17E-03	1.73E-02	0.0,0.0	0.34,0.34
negative regulation of aspartic-type peptidase activity	GO:1905246	2.17E-03	1.73E-02	0.0,0.0	0.34,0.34
positive regulation of early endosome to recycling endosome transport	GO:1902955	2.17E-03	1.73E-02	0.0,0.0	0.34,0.34

modulation by organism of defense-related nitric oxide production in other organism involved in symbiotic interaction	GO:0052302	2.17E-03	1.73E-02	0.0,0.0	0.34,0.34
GDP catabolic process	GO:0046712	2.17E-03	1.73E-02	0.0,0.0	0.34,0.34
positive regulation of opsonization	GO:1903028	2.17E-03	1.73E-02	0.0,0.0	0.34,0.34
purine deoxyribonucleoside diphosphate catabolic process	GO:0009184	2.17E-03	1.73E-02	0.0,0.0	0.34,0.34
dADP catabolic process	GO:0046057	2.17E-03	1.73E-02	0.0,0.0	0.34,0.34
positive regulation by organism of defense-related nitric oxide production in other organism involved in symbiotic interaction	GO:0052345	2.17E-03	1.73E-02	0.0,0.0	0.34,0.34
arginine deiminase pathway	GO:0019546	2.17E-03	1.73E-02	0.0,0.0	0.34,0.34
positive regulation by symbiont of host immune response	GO:0052556	2.17E-03	1.73E-02	0.0,0.0	0.34,0.34
chloramphenicol transport	GO:0042892	2.17E-03	1.73E-02	0.0,0.0	0.34,0.34
regulation of interleukin-4 production	GO:0032673	2.22E-03	1.76E-02	0.1,0.07	0.38,0.36
cellular lipid catabolic process	GO:0044242	2.23E-03	1.76E-02	-0.79,-0.76	0.04,0.05
cellular response to cytokine stimulus	GO:0071345	2.23E-03	1.76E-02	-2.79,-2.58	-0.71,-0.64
maintenance of location	GO:0051235	2.24E-03	1.76E-02	-1.04,-0.96	-0.05,-0.03
regulation of leukocyte activation	GO:0002694	2.25E-03	1.76E-02	-1.24,-1.2	-0.13,-0.12
nuclear transport	GO:0051169	2.40E-03	1.88E-02	-2.25,-2.07	-0.5,-0.44
response to transition metal nanoparticle	GO:1990267	2.43E-03	1.89E-02	-0.39,-0.44	0.19,0.17
cellular response to nitrogen compound	GO:1901699	2.43E-03	1.89E-02	-1.29,-1.26	-0.14,-0.14
positive regulation of cellular component organization	GO:0051130	2.46E-03	1.91E-02	-2.67,-2.76	-0.66,-0.7
autophagy	GO:0006914	2.49E-03	1.92E-02	-1.19,-1.11	-0.11,-0.08
innate immune response	GO:0045087	2.49E-03	1.92E-02	-2.46,-2.26	-0.58,-0.51
regulation of symbiosis, encompassing mutualism through parasitism	GO:0043903	2.50E-03	1.92E-02	-1.13,-0.99	-0.08,-0.04
negative regulation of cell adhesion	GO:0007162	2.57E-03	1.97E-02	-0.87,-0.83	0.01,0.02
protein localization to nucleus	GO:0034504	2.59E-03	1.98E-02	-1.32,-1.23	-0.15,-0.13
regulation of cell development	GO:0060284	2.61E-03	1.99E-02	-1.57,-1.76	-0.25,-0.33

regulation of MAP kinase activity	GO:0043405	2.61E-03	1.99E-02	0.32,0.34	0.46,0.46
positive regulation of cytoplasmic transport	GO:1903651	2.64E-03	2.00E-02	-1.34,-1.27	-0.16,-0.14
cytokine-mediated signaling pathway	GO:0019221	2.64E-03	2.00E-02	-1.63,-1.51	-0.27,-0.23
cellular process	GO:0009987	2.67E-03	2.02E-02	-29.27,-29.04	-10.61,-10.6
cellular metabolic process	GO:0044237	2.69E-03	2.03E-02	-22.4,-22.47	-8.03,-8.13
regulation of cellular protein localization	GO:1903827	2.74E-03	2.05E-02	-1.88,-1.78	-0.36,-0.34
positive regulation of nucleic acid-templated transcription	GO:1903508	2.74E-03	2.05E-02	-3.91,-3.9	-1.12,-1.13
positive regulation of transcription, DNA-templated	GO:0045893	2.74E-03	2.05E-02	-3.91,-3.9	-1.12,-1.13
cytoskeleton organization	GO:0007010	2.78E-03	2.06E-02	-5.69,-5.83	-1.79,-1.86
response to nitrogen compound	GO:1901698	2.78E-03	2.06E-02	-1.74,-1.7	-0.31,-0.3
positive regulation of interleukin-12 production	GO:0032735	2.80E-03	2.06E-02	0.08,0.02	0.37,0.34
purine ribonucleotide catabolic process	GO:0009154	2.80E-03	2.06E-02	-0.38,-0.35	0.2,0.21
respiratory burst	GO:0045730	2.80E-03	2.06E-02	-0.71,-0.65	0.07,0.09
negative regulation of telomere maintenance	GO:0032205	2.80E-03	2.06E-02	-0.04,0.06	0.32,0.36
positive regulation of leukocyte migration	GO:0002687	2.90E-03	2.12E-02	-0.37,-0.29	0.2,0.23
regulation of endocytosis	GO:0030100	2.90E-03	2.12E-02	-0.91,-0.88	-0.0,0.01
regulation of stress-activated MAPK cascade	GO:0032872	2.94E-03	2.14E-02	-0.1,0.1	0.3,0.38
interleukin-4 production	GO:0032633	2.95E-03	2.14E-02	0.1,0.07	0.38,0.36
ribonucleotide catabolic process	GO:0009261	2.95E-03	2.14E-02	-0.38,-0.35	0.2,0.21
regulation of stress-activated protein kinase signaling cascade	GO:0070302	2.98E-03	2.16E-02	-0.1,0.1	0.3,0.38
macromolecular complex subunit organization	GO:0043933	3.05E-03	2.20E-02	-5.26,-5.22	-1.63,-1.63
positive regulation of RNA biosynthetic process	GO:1902680	3.07E-03	2.21E-02	-3.91,-3.9	-1.12,-1.13
cellular protein metabolic process	GO:0044267	3.07E-03	2.21E-02	-11.33,-11.47	-3.9,-3.99
positive regulation of intracellular protein transport	GO:0090316	3.10E-03	2.21E-02	-1.34,-1.27	-0.16,-0.14
response to mechanical stimulus	GO:0009612	3.10E-03	2.21E-02	-1.02,-0.97	-0.04,-0.03
phosphatidylcholine biosynthetic process	GO:0006656	3.11E-03	2.21E-02	-0.31,-0.27	0.22,0.23
removal of superoxide radicals	GO:0019430	3.11E-03	2.21E-02	-0.51,-0.49	0.15,0.15
regulation of bone resorption	GO:0045124	3.11E-03	2.21E-02	-0.04,0.02	0.32,0.34

ERBB2 signaling pathway	GO:0038128	3.28E-03	2.31E-02	0.1,0.13	0.38,0.38
positive regulation of smooth muscle cell migration	GO:0014911	3.28E-03	2.31E-02	0.13,0.17	0.39,0.4
early endosome to late endosome transport	GO:0045022	3.28E-03	2.31E-02	-0.05,0.02	0.32,0.34
phagocytosis	GO:0006909	3.32E-03	2.33E-02	-1.07,-0.97	-0.06,-0.03
regulation of cellular process	GO:0050794	3.36E-03	2.34E-02	-23.02,-22.81	-8.27,-8.25
phospholipid transport	GO:0015914	3.36E-03	2.34E-02	-0.58,-0.54	0.12,0.13
Golgi organization	GO:0007030	3.36E-03	2.34E-02	-0.61,-0.66	0.11,0.09
regulation of cell-cell adhesion	GO:0022407	3.41E-03	2.37E-02	-0.32,-0.26	0.22,0.24
neurogenesis	GO:0022008	3.42E-03	2.37E-02	-2.75,-3.0	-0.69,-0.79
negative regulation of DNA biosynthetic process	GO:2000279	3.45E-03	2.37E-02	-0.04,0.06	0.32,0.36
regulation of protein autophosphorylation	GO:0031952	3.45E-03	2.37E-02	0.06,0.31	0.36,0.45
response to fluid shear stress	GO:0034405	3.45E-03	2.37E-02	0.14,0.14	0.39,0.39
production of molecular mediator of immune response	GO:0002440	3.52E-03	2.42E-02	-0.82,-0.75	0.03,0.05
response to peptide	GO:1901652	3.53E-03	2.42E-02	-1.16,-1.17	-0.09,-0.1
positive regulation of immune system process	GO:0002684	3.58E-03	2.44E-02	-2.05,-1.87	-0.43,-0.37
cellular response to superoxide	GO:0071451	3.62E-03	2.45E-02	-0.51,-0.49	0.15,0.15
cellular response to oxygen radical	GO:0071450	3.62E-03	2.45E-02	-0.51,-0.49	0.15,0.15
positive regulation of cytokine production involved in immune response	GO:0002720	3.62E-03	2.45E-02	-0.35,-0.3	0.21,0.22
single-organism nuclear import	GO:1902593	3.70E-03	2.49E-02	-1.7,-1.56	-0.29,-0.25
entry into host	GO:0044409	3.72E-03	2.49E-02	1.69,1.52	0.97,0.91
entry into host cell	GO:0030260	3.72E-03	2.49E-02	1.69,1.52	0.97,0.91
entry into cell of other organism involved in symbiotic interaction	GO:0051806	3.72E-03	2.49E-02	1.69,1.52	0.97,0.91
entry into other organism involved in symbiotic interaction	GO:0051828	3.72E-03	2.49E-02	1.69,1.52	0.97,0.91
positive regulation of RNA metabolic process	GO:0051254	3.78E-03	2.52E-02	-3.91,-3.9	-1.12,-1.13
reactive oxygen species metabolic process	GO:0072593	3.79E-03	2.52E-02	-1.04,-0.92	-0.05,-0.01
toxin transport	GO:1901998	3.79E-03	2.52E-02	-2.19,-2.24	-0.48,-0.51
positive regulation of gene expression	GO:0010628	3.86E-03	2.56E-02	-5.13,-5.06	-1.58,-1.57

organic hydroxy compound biosynthetic process	GO:1901617	3.93E-03	2.60E-02	-0.83,-0.77	0.03,0.05
nucleoside catabolic process	GO:0009164	3.97E-03	2.62E-02	-0.38,-0.35	0.2,0.21
vascular endothelial growth factor receptor signaling pathway	GO:0048010	4.10E-03	2.65E-02	-0.19,-0.14	0.27,0.28
response to superoxide	GO:0000303	4.16E-03	2.65E-02	-0.51,-0.49	0.15,0.15
response to pH	GO:0009268	4.16E-03	2.65E-02	-0.03,0.07	0.33,0.36
negative regulation of binding	GO:0051100	4.26E-03	2.65E-02	-0.62,-0.59	0.11,0.11
dihydrofolate metabolic process	GO:0046452	4.34E-03	2.65E-02	0.0,0.0	0.34,0.34
positive regulation of aconitate hydratase activity	GO:1904234	4.34E-03	2.65E-02	0.0,0.0	0.34,0.34
viral penetration into host nucleus	GO:0075732	4.34E-03	2.65E-02	0.0,0.0	0.34,0.34
histone H3-R26 citrullination	GO:0036413	4.34E-03	2.65E-02	0.0,0.0	0.34,0.34
AMP catabolic process	GO:0006196	4.34E-03	2.65E-02	0.0,0.0	0.34,0.34
regulation of aspartic-type peptidase activity	GO:1905245	4.34E-03	2.65E-02	0.0,0.0	0.34,0.34
positive regulation of female gonad development	GO:2000196	4.34E-03	2.65E-02	0.0,0.0	0.34,0.34
regulation of early endosome to recycling endosome transport	GO:1902954	4.34E-03	2.65E-02	0.0,0.0	0.34,0.34
hypochlorous acid metabolic process	GO:0002148	4.34E-03	2.65E-02	0.0,0.0	0.34,0.34
regulation of metalloendopeptidase activity involved in amyloid precursor protein catabolic process	GO:1902962	4.34E-03	2.65E-02	0.0,0.0	0.34,0.34
regulation of succinate dehydrogenase activity	GO:1904229	4.34E-03	2.65E-02	0.0,0.0	0.34,0.34
positive regulation of interleukin-18 production	GO:0032741	4.34E-03	2.65E-02	0.0,0.0	0.34,0.34
metal incorporation into metallo-sulfur cluster	GO:0018282	4.34E-03	2.65E-02	0.0,0.0	0.34,0.34
regulation of progesterone secretion	GO:2000870	4.34E-03	2.65E-02	0.0,0.0	0.34,0.34
hypochlorous acid biosynthetic process	GO:0002149	4.34E-03	2.65E-02	0.0,0.0	0.34,0.34
regulation of aspartic-type endopeptidase activity involved in amyloid precursor protein catabolic process	GO:1902959	4.34E-03	2.65E-02	0.0,0.0	0.34,0.34
negative regulation of metalloendopeptidase activity involved in	GO:1902963	4.34E-03	2.65E-02	0.0,0.0	0.34,0.34

amyloid precursor protein catabolic process					
response to defense-related nitric oxide production by other organism involved in symbiotic interaction	GO:0052551	4.34E-03	2.65E-02	0.0,0.0	0.34,0.34
positive regulation of succinate dehydrogenase activity	GO:1904231	4.34E-03	2.65E-02	0.0,0.0	0.34,0.34
asymmetric Golgi ribbon formation	GO:0090164	4.34E-03	2.65E-02	0.0,0.0	0.34,0.34
angiotensin-activated signaling pathway involved in heart process	GO:0086098	4.34E-03	2.65E-02	0.0,0.0	0.34,0.34
granuloma formation	GO:0002432	4.34E-03	2.65E-02	0.0,0.0	0.34,0.34
response to defense-related host nitric oxide production	GO:0052565	4.34E-03	2.65E-02	0.0,0.0	0.34,0.34
myoblast migration involved in skeletal muscle regeneration	GO:0014839	4.34E-03	2.65E-02	0.0,0.0	0.34,0.34
regulation of aconitate hydratase activity	GO:1904232	4.34E-03	2.65E-02	0.0,0.0	0.34,0.34
intestinal epithelial cell migration	GO:0061582	4.34E-03	2.65E-02	0.0,0.0	0.34,0.34
multi-organism nuclear import	GO:1902594	4.34E-03	2.65E-02	0.0,0.0	0.34,0.34
iron incorporation into metallo-sulfur cluster	GO:0018283	4.34E-03	2.65E-02	0.0,0.0	0.34,0.34
triglyceride catabolic process	GO:0019433	4.34E-03	2.65E-02	-0.27,-0.29	0.24,0.23
positive regulation of interleukin-8 production	GO:0032757	4.34E-03	2.65E-02	-0.55,-0.47	0.13,0.16
positive regulation of oxidoreductase activity	GO:0051353	4.34E-03	2.65E-02	-0.27,-0.31	0.24,0.22
regulation of biological process	GO:0050789	4.37E-03	2.66E-02	-23.41,-23.15	-8.41,-8.38
embryo development	GO:0009790	4.42E-03	2.69E-02	1.99,1.96	1.08,1.07
iron ion homeostasis	GO:0055072	4.50E-03	2.73E-02	-0.39,-0.44	0.19,0.17
reproductive structure development	GO:0048608	4.56E-03	2.76E-02	-0.36,-0.31	0.2,0.22
organic substance metabolic process	GO:0071704	4.65E-03	2.81E-02	-24.68,-24.78	-8.89,-9.0
response to oxygen radical	GO:0000305	4.73E-03	2.83E-02	-0.51,-0.49	0.15,0.15
regulation of bone remodeling	GO:0046850	4.73E-03	2.83E-02	-0.04,0.02	0.32,0.34
DNA biosynthetic process	GO:0071897	4.74E-03	2.83E-02	-0.68,-0.66	0.08,0.09
negative regulation of organelle organization	GO:0010639	4.76E-03	2.83E-02	-0.58,-0.57	0.12,0.12
embryonic organ development	GO:0048568	4.76E-03	2.83E-02	0.36,0.35	0.47,0.47

reproductive system development	GO:0061458	4.76E-03	2.83E-02	-0.36,-0.31	0.2,0.22
triglyceride metabolic process	GO:0006641	4.84E-03	2.87E-02	-0.44,-0.43	0.17,0.17
hematopoietic progenitor cell differentiation	GO:0002244	4.84E-03	2.87E-02	-0.74,-0.65	0.06,0.09
regulation of interleukin-10 production	GO:0032653	4.93E-03	2.92E-02	0.08,0.02	0.37,0.34
response to abiotic stimulus	GO:0009628	5.02E-03	2.97E-02	-2.14,-2.1	-0.46,-0.45
cellular response to organic cyclic compound	GO:0071407	5.12E-03	3.02E-02	-3.45,-3.32	-0.95,-0.92
modification by symbiont of host morphology or physiology	GO:0044003	5.34E-03	3.13E-02	-0.92,-0.95	-0.0,-0.02
interleukin-10 production	GO:0032613	5.34E-03	3.13E-02	0.08,0.02	0.37,0.34
regulation of telomerase activity	GO:0051972	5.34E-03	3.13E-02	-0.05,0.02	0.32,0.34
anatomical structure morphogenesis	GO:0009653	5.35E-03	3.13E-02	-4.54,-4.66	-1.36,-1.42
secondary alcohol metabolic process	GO:1902652	5.37E-03	3.13E-02	-0.17,-0.17	0.27,0.27
embryo development ending in birth or egg hatching	GO:0009792	5.61E-03	3.27E-02	1.48,1.51	0.89,0.91
positive regulation of molecular function	GO:0044093	5.62E-03	3.27E-02	-3.05,-3.09	-0.8,-0.83
cholesterol metabolic process	GO:0008203	5.65E-03	3.27E-02	-0.17,-0.17	0.27,0.27
cellular response to insulin stimulus	GO:0032869	5.65E-03	3.27E-02	-0.63,-0.71	0.1,0.07
glycosyl compound catabolic process	GO:1901658	5.76E-03	3.33E-02	-0.38,-0.35	0.2,0.21
signal transduction	GO:0007165	5.80E-03	3.34E-02	-13.68,-13.39	-4.77,-4.71
single organism cell adhesion	GO:0098602	5.82E-03	3.35E-02	-1.68,-1.62	-0.29,-0.27
cellular biogenic amine metabolic process	GO:0006576	5.84E-03	3.35E-02	-0.57,-0.5	0.13,0.15
trivalent inorganic cation transport	GO:0072512	5.98E-03	3.38E-02	-0.49,-0.54	0.16,0.13
ferric iron transport	GO:0015682	5.98E-03	3.38E-02	-0.49,-0.54	0.16,0.13
peptidyl-tyrosine autophosphorylation	GO:0038083	6.03E-03	3.38E-02	-0.19,-0.14	0.27,0.28
positive regulation of NF-kappaB transcription factor activity	GO:0051092	6.03E-03	3.38E-02	-0.13,-0.13	0.29,0.29
single-organism organelle organization	GO:1902589	6.10E-03	3.38E-02	-7.04,-7.08	-2.29,-2.33
lipopolysaccharide-mediated signaling pathway	GO:0031663	6.20E-03	3.38E-02	-0.35,-0.3	0.21,0.22
negative regulation of cell-substrate adhesion	GO:0010812	6.20E-03	3.38E-02	0.11,0.21	0.38,0.41

intracellular estrogen receptor signaling pathway	GO:0030520	6.20E-03	3.38E-02	2.59,2.52	1.31,1.29
regulation of granulocyte chemotaxis	GO:0071622	6.20E-03	3.38E-02	-0.24,-0.13	0.25,0.28
regulation of anatomical structure morphogenesis	GO:0022603	6.21E-03	3.38E-02	-2.24,-2.4	-0.5,-0.57
cellular response to stress	GO:0033554	6.22E-03	3.38E-02	-5.05,-5.01	-1.55,-1.55
regulation of organelle organization	GO:0033043	6.26E-03	3.38E-02	-2.57,-2.6	-0.62,-0.64
extracellular matrix organization	GO:0030198	6.27E-03	3.38E-02	-1.96,-1.88	-0.39,-0.37
defense response to bacterium	GO:0042742	6.28E-03	3.38E-02	-1.41,-1.31	-0.19,-0.16
protein import into nucleus	GO:0006606	6.28E-03	3.38E-02	-1.7,-1.56	-0.29,-0.25
protein targeting to nucleus	GO:0044744	6.28E-03	3.38E-02	-1.7,-1.56	-0.29,-0.25
extracellular structure organization	GO:0043062	6.30E-03	3.38E-02	-1.96,-1.88	-0.39,-0.37
regulation of T cell proliferation	GO:0042129	6.33E-03	3.38E-02	-0.13,-0.13	0.29,0.29
ERBB signaling pathway	GO:0038127	6.34E-03	3.38E-02	-0.76,-0.73	0.06,0.06
positive regulation of nitrogen compound metabolic process	GO:0051173	6.36E-03	3.38E-02	-5.25,-5.16	-1.62,-1.61
axon guidance	GO:0007411	6.39E-03	3.38E-02	-1.03,-1.02	-0.05,-0.05
regulation of nervous system development	GO:0051960	6.43E-03	3.38E-02	-1.44,-1.65	-0.2,-0.29
neuron projection guidance	GO:0097485	6.49E-03	3.38E-02	-1.03,-1.02	-0.05,-0.05
central nervous system myelin formation	GO:0032289	6.50E-03	3.38E-02	0.0,0.0	0.34,0.34
T-helper 1 cell activation	GO:0035711	6.50E-03	3.38E-02	0.0,0.0	0.34,0.34
D-xylose metabolic process	GO:0042732	6.50E-03	3.38E-02	0.0,0.0	0.34,0.34
dADP metabolic process	GO:0046056	6.50E-03	3.38E-02	0.0,0.0	0.34,0.34
granzyme B production	GO:0071613	6.50E-03	3.38E-02	0.0,0.0	0.34,0.34
cobalamin biosynthetic process	GO:0009236	6.50E-03	3.38E-02	0.0,0.0	0.34,0.34
regulation of opsonization	GO:1903027	6.50E-03	3.38E-02	0.0,0.0	0.34,0.34
regulation of granzyme B production	GO:0071661	6.50E-03	3.38E-02	0.0,0.0	0.34,0.34
positive regulation by symbiont of host defense response	GO:0052509	6.50E-03	3.38E-02	0.0,0.0	0.34,0.34
MAPK import into nucleus	GO:0000189	6.50E-03	3.38E-02	0.0,0.0	0.34,0.34
modulation by organism of immune response of other organism involved in symbiotic interaction	GO:0052552	6.50E-03	3.38E-02	0.0,0.0	0.34,0.34

positive regulation by organism of defense response of other organism involved in symbiotic interaction	GO:0052510	6.50E-03	3.38E-02	0.0,0.0	0.34,0.34
cellular response to triacyl bacterial lipopeptide	GO:0071727	6.50E-03	3.38E-02	0.0,0.0	0.34,0.34
cellular response to progesterone stimulus	GO:0071393	6.50E-03	3.38E-02	0.0,0.0	0.34,0.34
positive regulation of granzyme B production	GO:0071663	6.50E-03	3.38E-02	0.0,0.0	0.34,0.34
modulation by symbiont of host immune response	GO:0052553	6.50E-03	3.38E-02	0.0,0.0	0.34,0.34
cell surface pattern recognition receptor signaling pathway	GO:0002752	6.50E-03	3.38E-02	0.0,0.0	0.34,0.34
antibiotic transport	GO:0042891	6.50E-03	3.38E-02	0.0,0.0	0.34,0.34
diacylglycerol catabolic process	GO:0046340	6.50E-03	3.38E-02	0.0,0.0	0.34,0.34
protein targeting to lysosome involved in chaperone-mediated autophagy	GO:0061740	6.50E-03	3.38E-02	0.0,0.0	0.34,0.34
modulation by symbiont of host defense response	GO:0052031	6.50E-03	3.38E-02	0.0,0.0	0.34,0.34
cell septum assembly	GO:0090529	6.50E-03	3.38E-02	0.0,0.0	0.34,0.34
guanosine-containing compound catabolic process	GO:1901069	6.50E-03	3.38E-02	0.0,0.0	0.34,0.34
regulation of neurofibrillary tangle assembly	GO:1902996	6.50E-03	3.38E-02	0.0,0.0	0.34,0.34
dGDP metabolic process	GO:0046066	6.50E-03	3.38E-02	0.0,0.0	0.34,0.34
modulation by organism of defense response of other organism involved in symbiotic interaction	GO:0052255	6.50E-03	3.38E-02	0.0,0.0	0.34,0.34
barrier septum assembly	GO:0000917	6.50E-03	3.38E-02	0.0,0.0	0.34,0.34
induction by organism of defense response of other organism involved in symbiotic interaction	GO:0052251	6.50E-03	3.38E-02	0.0,0.0	0.34,0.34
small molecule catabolic process	GO:0044282	6.59E-03	3.42E-02	-1.07,-1.07	-0.06,-0.07
tetrapyrrole biosynthetic process	GO:0033014	6.65E-03	3.44E-02	0.53,0.58	0.54,0.55
cellular response to hormone stimulus	GO:0032870	6.67E-03	3.45E-02	-3.63,-3.62	-1.02,-1.03
developmental process involved in reproduction	GO:0003006	6.77E-03	3.49E-02	-1.34,-1.24	-0.16,-0.13
purine nucleotide catabolic process	GO:0006195	6.88E-03	3.54E-02	-0.38,-0.35	0.2,0.21
regulation of interleukin-12 production	GO:0032655	6.88E-03	3.54E-02	0.08,0.02	0.37,0.34

regulation of multi-organism process	GO:0043900	7.11E-03	3.65E-02	-1.26,-1.14	-0.13,-0.09
regulation of molecular function	GO:0065009	7.15E-03	3.67E-02	-4.06,-4.13	-1.18,-1.22
positive regulation of MAP kinase activity	GO:0043406	7.23E-03	3.70E-02	0.8,0.79	0.64,0.63
modification of morphology or physiology of other organism	GO:0035821	7.28E-03	3.72E-02	0.21,0.14	0.42,0.39
interleukin-12 production	GO:0032615	7.36E-03	3.75E-02	0.08,0.02	0.37,0.34
positive regulation of Wnt signaling pathway	GO:0030177	7.39E-03	3.76E-02	0.21,0.14	0.42,0.39
regulation of T cell activation	GO:0050863	7.44E-03	3.78E-02	0.25,0.25	0.43,0.43
stress-activated MAPK cascade	GO:0051403	7.59E-03	3.85E-02	-0.1,0.1	0.3,0.38
bone resorption	GO:0045453	7.85E-03	3.96E-02	-0.04,0.02	0.32,0.34
regulation of lamellipodium organization	GO:1902743	7.85E-03	3.96E-02	0.02,0.06	0.35,0.36
positive regulation of metabolic process	GO:0009893	7.88E-03	3.97E-02	-7.22,-7.33	-2.36,-2.43
positive regulation of macromolecule biosynthetic process	GO:0010557	7.97E-03	4.01E-02	-3.91,-3.9	-1.12,-1.13
positive regulation of macromolecule metabolic process	GO:0010604	8.01E-03	4.02E-02	-6.11,-6.21	-1.94,-2.0
phagosome maturation	GO:0090382	8.10E-03	4.06E-02	-0.49,-0.48	0.16,0.16
macromolecular complex assembly	GO:0065003	8.12E-03	4.06E-02	-3.76,-3.64	-1.07,-1.04
regulation of leukocyte migration	GO:0002685	8.20E-03	4.09E-02	-0.37,-0.29	0.2,0.23
negative regulation of developmental process	GO:0051093	8.29E-03	4.13E-02	-1.78,-1.78	-0.33,-0.34
neuron projection development	GO:0031175	8.44E-03	4.13E-02	-1.99,-2.34	-0.4,-0.55
leukocyte differentiation	GO:0002521	8.51E-03	4.13E-02	-0.32,-0.26	0.22,0.24
sterol metabolic process	GO:0016125	8.56E-03	4.13E-02	-0.17,-0.17	0.27,0.27
response to toxic substance	GO:0009636	8.58E-03	4.13E-02	-0.85,-0.79	0.02,0.04
regulation of leukocyte cell-cell adhesion	GO:1903037	8.58E-03	4.13E-02	0.25,0.25	0.43,0.43
regulation of interleukin-8 production	GO:0032677	8.61E-03	4.13E-02	-0.55,-0.47	0.13,0.16
regulation of glial cell differentiation	GO:0045685	8.61E-03	4.13E-02	-0.35,-0.28	0.21,0.23
peptidyl-arginine methylation, to symmetrical-dimethyl arginine	GO:0019918	8.65E-03	4.13E-02	0.0,0.0	0.34,0.34
regulation of Golgi inheritance	GO:0090170	8.65E-03	4.13E-02	0.0,0.0	0.34,0.34

early endosome to recycling endosome transport	GO:0061502	8.65E-03	4.13E-02	0.0,0.0	0.34,0.34
detection of diacyl bacterial lipopeptide	GO:0042496	8.65E-03	4.13E-02	0.0,0.0	0.34,0.34
xylulose metabolic process	GO:0005997	8.65E-03	4.13E-02	0.0,0.0	0.34,0.34
proprioception	GO:0019230	8.65E-03	4.13E-02	0.0,0.0	0.34,0.34
purine deoxyribonucleoside diphosphate metabolic process	GO:0009182	8.65E-03	4.13E-02	0.0,0.0	0.34,0.34
unidimensional cell growth	GO:0009826	8.65E-03	4.13E-02	0.0,0.0	0.34,0.34
progesterone secretion	GO:0042701	8.65E-03	4.13E-02	0.0,0.0	0.34,0.34
microglia differentiation	GO:0014004	8.65E-03	4.13E-02	0.0,0.0	0.34,0.34
positive regulation of pinocytosis	GO:0048549	8.65E-03	4.13E-02	0.0,0.0	0.34,0.34
cellular response to diacyl bacterial lipopeptide	GO:0071726	8.65E-03	4.13E-02	0.0,0.0	0.34,0.34
neurofibrillary tangle assembly	GO:1902988	8.65E-03	4.13E-02	0.0,0.0	0.34,0.34
response to molecule of fungal origin	GO:0002238	8.65E-03	4.13E-02	0.0,0.0	0.34,0.34
positive regulation of sodium:proton antiporter activity	GO:0032417	8.65E-03	4.13E-02	0.0,0.0	0.34,0.34
negative regulation of metalloendopeptidase activity	GO:1904684	8.65E-03	4.13E-02	0.0,0.0	0.34,0.34
microglia development	GO:0014005	8.65E-03	4.13E-02	0.0,0.0	0.34,0.34
protein targeting to vacuole involved in autophagy	GO:0071211	8.65E-03	4.13E-02	0.0,0.0	0.34,0.34
organophosphate ester transport	GO:0015748	8.68E-03	4.13E-02	-0.58,-0.54	0.12,0.13
myeloid cell development	GO:0061515	8.87E-03	4.22E-02	0.02,0.01	0.35,0.34
axon development	GO:0061564	8.91E-03	4.23E-02	-1.58,-1.69	-0.25,-0.3
regulation of hemopoiesis	GO:1903706	9.41E-03	4.46E-02	-0.84,-0.79	0.02,0.04
multicellular organismal homeostasis	GO:0048871	9.58E-03	4.53E-02	0.03,0.1	0.35,0.37
organonitrogen compound catabolic process	GO:1901565	9.64E-03	4.56E-02	-1.96,-1.82	-0.39,-0.35
neuron differentiation	GO:0030182	9.66E-03	4.56E-02	-2.35,-2.67	-0.54,-0.67
transition metal ion homeostasis	GO:0055076	9.82E-03	4.62E-02	-0.39,-0.44	0.19,0.17
receptor-mediated endocytosis	GO:0006898	9.90E-03	4.66E-02	-0.56,-0.43	0.13,0.17
response to fungus	GO:0009620	9.95E-03	4.66E-02	-0.59,-0.51	0.12,0.14
positive regulation of DNA biosynthetic process	GO:2000573	9.95E-03	4.66E-02	-0.05,0.02	0.32,0.34
interleukin-8 production	GO:0032637	1.02E-02	4.78E-02	-0.55,-0.47	0.13,0.16

purine-containing compound catabolic process	GO:0072523	1.02E-02	4.78E-02	-0.38,-0.35	0.2,0.21
positive regulation of protein complex assembly	GO:0031334	1.03E-02	4.79E-02	-0.93,-0.86	-0.01,0.01
toll-like receptor TLR6:TLR2 signaling pathway	GO:0038124	1.05E-02	4.82E-02	-0.35,-0.3	0.21,0.22
positive regulation of cellular carbohydrate metabolic process	GO:0010676	1.05E-02	4.82E-02	0.15,0.19	0.4,0.41
T cell proliferation	GO:0042098	1.06E-02	4.82E-02	-0.13,-0.13	0.29,0.29
regulation of ossification	GO:0030278	1.06E-02	4.82E-02	-0.55,-0.48	0.13,0.16
protein autophosphorylation	GO:0046777	1.07E-02	4.82E-02	-0.72,-0.9	0.07,-0.0
toll-like receptor TLR1:TLR2 signaling pathway	GO:0038123	1.08E-02	4.82E-02	-0.35,-0.3	0.21,0.22
cellular response to ammonium ion	GO:0071242	1.08E-02	4.82E-02	-0.36,-0.37	0.21,0.2
positive regulation of endocytic recycling	GO:2001137	1.08E-02	4.82E-02	0.0,0.0	0.34,0.34
positive regulation of ER to Golgi vesicle-mediated transport	GO:1902953	1.08E-02	4.82E-02	0.0,0.0	0.34,0.34
positive regulation of platelet-derived growth factor receptor signaling pathway	GO:0010641	1.08E-02	4.82E-02	0.0,0.0	0.34,0.34
glucuronate catabolic process to xylulose 5-phosphate	GO:0019640	1.08E-02	4.82E-02	0.0,0.0	0.34,0.34
oxygen metabolic process	GO:0072592	1.08E-02	4.82E-02	0.0,0.0	0.34,0.34
histone citrullination	GO:0036414	1.08E-02	4.82E-02	0.0,0.0	0.34,0.34
regulation of caveolin-mediated endocytosis	GO:2001286	1.08E-02	4.82E-02	0.0,0.0	0.34,0.34
purine ribonucleoside diphosphate catabolic process	GO:0009181	1.08E-02	4.82E-02	0.0,0.0	0.34,0.34
purine nucleoside diphosphate catabolic process	GO:0009137	1.08E-02	4.82E-02	0.0,0.0	0.34,0.34
positive regulation of protein localization to early endosome	GO:1902966	1.08E-02	4.82E-02	0.0,0.0	0.34,0.34
glucuronate catabolic process	GO:0006064	1.08E-02	4.82E-02	0.0,0.0	0.34,0.34
NADPH oxidation	GO:0070995	1.08E-02	4.82E-02	0.0,0.0	0.34,0.34
protein localization to bicellular tight junction	GO:1902396	1.08E-02	4.82E-02	0.0,0.0	0.34,0.34
response to methotrexate	GO:0031427	1.08E-02	4.82E-02	0.0,0.0	0.34,0.34

regulation of protein localization to early endosome	GO:1902965	1.08E-02	4.82E-02	0.0,0.0	0.34,0.34
detection of triacyl bacterial lipopeptide	GO:0042495	1.08E-02	4.82E-02	0.0,0.0	0.34,0.34
progesterone receptor signaling pathway	GO:0050847	1.08E-02	4.82E-02	0.0,0.0	0.34,0.34
positive regulation of CD8-positive, alpha-beta T cell proliferation	GO:2000566	1.08E-02	4.82E-02	0.0,0.0	0.34,0.34
xylulose 5-phosphate biosynthetic process	GO:1901159	1.08E-02	4.82E-02	0.0,0.0	0.34,0.34
xylulose 5-phosphate metabolic process	GO:0051167	1.08E-02	4.82E-02	0.0,0.0	0.34,0.34
positive regulation of intracellular transport	GO:0032388	1.09E-02	4.86E-02	-1.34,-1.27	-0.16,-0.14
cellular response to stimulus	GO:0051716	1.09E-02	4.87E-02	-15.71,-15.37	-5.53,-5.45
macrophage activation	GO:0042116	1.11E-02	4.91E-02	-0.42,-0.39	0.18,0.19
regulation of cytokine production involved in immune response	GO:0002718	1.11E-02	4.91E-02	-0.35,-0.3	0.21,0.22
peptidyl-arginine modification	GO:0018195	1.11E-02	4.91E-02	0.26,0.25	0.43,0.43
cell morphogenesis involved in differentiation	GO:0000904	1.12E-02	4.94E-02	-2.01,-2.36	-0.41,-0.55
positive regulation of nucleobase-containing compound metabolic process	GO:0045935	1.12E-02	4.94E-02	-3.91,-3.9	-1.12,-1.13
regulation of tissue remodeling	GO:0034103	1.14E-02	5.01E-02	-0.04,0.02	0.32,0.34
homotypic cell-cell adhesion	GO:0034109	1.14E-02	5.03E-02	-0.32,-0.26	0.22,0.24
intracellular protein transport	GO:0006886	1.15E-02	5.03E-02	-4.6,-4.46	-1.38,-1.34
in utero embryonic development	GO:0001701	1.15E-02	5.03E-02	0.05,0.05	0.36,0.35
primary metabolic process	GO:0044238	1.15E-02	5.03E-02	-23.84,-23.97	-8.58,-8.69
glycerophospholipid metabolic process	GO:0006650	1.17E-02	5.09E-02	-0.95,-0.84	-0.02,0.02
astrocyte differentiation	GO:0048708	1.17E-02	5.09E-02	-0.27,-0.29	0.24,0.22
regulation of phagocytosis	GO:0050764	1.17E-02	5.09E-02	-0.42,-0.39	0.18,0.19
tissue development	GO:0009888	1.17E-02	5.09E-02	-2.34,-2.36	-0.53,-0.55
glycerophospholipid biosynthetic process	GO:0046474	1.22E-02	5.28E-02	-0.77,-0.68	0.05,0.08
response to oxygen levels	GO:0070482	1.22E-02	5.28E-02	-1.03,-0.91	-0.05,-0.01
cellular response to drug	GO:0035690	1.23E-02	5.31E-02	-0.42,-0.58	0.18,0.12
protein metabolic process	GO:0019538	1.23E-02	5.32E-02	-13.64,-13.74	-4.76,-4.84
ammonium ion metabolic process	GO:0097164	1.23E-02	5.33E-02	-0.57,-0.5	0.13,0.15

regulation of intracellular protein transport	GO:0033157	1.24E-02	5.33E-02	-1.34,-1.27	-0.16,-0.14
regulation of osteoclast differentiation	GO:0045670	1.26E-02	5.36E-02	-0.38,-0.35	0.2,0.21
lamellipodium assembly	GO:0030032	1.26E-02	5.36E-02	-0.63,-0.68	0.1,0.08
regulation of cellular response to heat	GO:1900034	1.26E-02	5.36E-02	-0.25,-0.31	0.25,0.22
organic acid metabolic process	GO:0006082	1.28E-02	5.36E-02	-2.0,-1.99	-0.41,-0.41
lipid catabolic process	GO:0016042	1.29E-02	5.36E-02	-0.79,-0.76	0.04,0.05
response to staurosporine	GO:0072733	1.30E-02	5.36E-02	0.0,0.0	0.34,0.34
regulation of CD8-positive, alpha-beta T cell proliferation	GO:2000564	1.30E-02	5.36E-02	0.0,0.0	0.34,0.34
non-canonical Wnt signaling pathway via JNK cascade	GO:0038031	1.30E-02	5.36E-02	0.0,0.0	0.34,0.34
cellular response to bacterial lipoprotein	GO:0071220	1.30E-02	5.36E-02	0.0,0.0	0.34,0.34
positive regulation of metalloenzyme activity	GO:0048554	1.30E-02	5.36E-02	0.0,0.0	0.34,0.34
positive regulation of CD8-positive, alpha-beta T cell activation	GO:2001187	1.30E-02	5.36E-02	0.0,0.0	0.34,0.34
ferric iron import across plasma membrane	GO:0098706	1.30E-02	5.36E-02	0.0,0.0	0.34,0.34
response to diacyl bacterial lipopeptide	GO:0071724	1.30E-02	5.36E-02	0.0,0.0	0.34,0.34
positive regulation of integrin activation	GO:0033625	1.30E-02	5.36E-02	0.0,0.0	0.34,0.34
regulation of sodium:proton antiporter activity	GO:0032415	1.30E-02	5.36E-02	0.0,0.0	0.34,0.34
regulation of xenophagy	GO:1904415	1.30E-02	5.36E-02	0.0,0.0	0.34,0.34
negative regulation of metallopeptidase activity	GO:1905049	1.30E-02	5.36E-02	0.0,0.0	0.34,0.34
regulation of metalloendopeptidase activity	GO:1904683	1.30E-02	5.36E-02	0.0,0.0	0.34,0.34
cellular response to bacterial lipopeptide	GO:0071221	1.30E-02	5.36E-02	0.0,0.0	0.34,0.34
regulation of interleukin-18 production	GO:0032661	1.30E-02	5.36E-02	0.0,0.0	0.34,0.34
protein citrullination	GO:0018101	1.30E-02	5.36E-02	0.0,0.0	0.34,0.34
regulation of dendritic cell cytokine production	GO:0002730	1.30E-02	5.36E-02	0.0,0.0	0.34,0.34
snRNA transcription from RNA polymerase III promoter	GO:0042796	1.30E-02	5.36E-02	0.0,0.0	0.34,0.34
cellular response to staurosporine	GO:0072734	1.30E-02	5.36E-02	0.0,0.0	0.34,0.34
response to triacyl bacterial lipopeptide	GO:0071725	1.30E-02	5.36E-02	0.0,0.0	0.34,0.34

positive regulation of xenophagy	GO:1904417	1.30E-02	5.36E-02	0.0,0.0	0.34,0.34
lysosomal protein catabolic process	GO:1905146	1.30E-02	5.36E-02	0.0,0.0	0.34,0.34
toll-like receptor 2 signaling pathway	GO:0034134	1.32E-02	5.44E-02	-0.35,-0.3	0.21,0.22
regulation of neurogenesis	GO:0050767	1.32E-02	5.44E-02	-1.09,-1.28	-0.07,-0.15
cell morphogenesis involved in neuron differentiation	GO:0048667	1.32E-02	5.44E-02	-1.7,-2.01	-0.3,-0.42
dephosphorylation	GO:0016311	1.32E-02	5.44E-02	-0.93,-0.98	-0.01,-0.03
glial cell differentiation	GO:0010001	1.34E-02	5.51E-02	-0.48,-0.44	0.16,0.17
enzyme linked receptor protein signaling pathway	GO:0007167	1.34E-02	5.51E-02	-1.74,-1.73	-0.31,-0.32
mammary gland epithelium development	GO:0061180	1.35E-02	5.52E-02	0.04,0.06	0.35,0.36
glycosphingolipid metabolic process	GO:0006687	1.35E-02	5.52E-02	-0.41,-0.44	0.18,0.17
ATP metabolic process	GO:0046034	1.35E-02	5.52E-02	-0.14,-0.17	0.29,0.27
regulation of peptidase activity	GO:0052547	1.35E-02	5.52E-02	-1.02,-0.99	-0.04,-0.04
animal organ morphogenesis	GO:0009887	1.35E-02	5.52E-02	-1.38,-1.39	-0.18,-0.19
fibril organization	GO:0097435	1.36E-02	5.52E-02	-1.34,-1.27	-0.16,-0.14
negative regulation of homeostatic process	GO:0032845	1.36E-02	5.52E-02	-0.19,-0.14	0.27,0.28
organophosphate catabolic process	GO:0046434	1.37E-02	5.56E-02	-0.64,-0.58	0.1,0.12
regulation of JNK cascade	GO:0046328	1.37E-02	5.56E-02	0.25,0.44	0.43,0.5
phosphatidylcholine metabolic process	GO:0046470	1.38E-02	5.59E-02	-0.31,-0.27	0.22,0.23
positive regulation of carbohydrate metabolic process	GO:0045913	1.42E-02	5.71E-02	0.15,0.19	0.4,0.41
protein maturation	GO:0051604	1.45E-02	5.83E-02	0.41,0.4	0.49,0.48
regulation of lymphocyte proliferation	GO:0050670	1.46E-02	5.86E-02	-0.13,-0.13	0.29,0.29
endosomal transport	GO:0016197	1.46E-02	5.87E-02	-0.67,-0.63	0.09,0.1
regulation of mononuclear cell proliferation	GO:0032944	1.47E-02	5.87E-02	-0.13,-0.13	0.29,0.29
regulation of cellular response to stress	GO:0080135	1.48E-02	5.87E-02	-1.1,-1.38	-0.07,-0.18
positive regulation of organelle organization	GO:0010638	1.48E-02	5.87E-02	-1.72,-1.69	-0.31,-0.3
regulation of DNA metabolic process	GO:0051052	1.50E-02	5.87E-02	-0.28,-0.07	0.23,0.31
induction of bacterial agglutination	GO:0043152	1.51E-02	5.87E-02	0.0,0.0	0.34,0.34
response to immune response of other	GO:0052564	1.51E-02	5.87E-02	0.0,0.0	0.34,0.34

organism involved in symbiotic interaction					
chaperone cofactor-dependent protein refolding	GO:0070389	1.51E-02	5.87E-02	0.0,0.0	0.34,0.34
protein localization to early endosome	GO:1902946	1.51E-02	5.87E-02	0.0,0.0	0.34,0.34
regulation of entry of bacterium into host cell	GO:2000535	1.51E-02	5.87E-02	0.0,0.0	0.34,0.34
non-canonical Wnt signaling pathway via MAPK cascade	GO:0038030	1.51E-02	5.87E-02	0.0,0.0	0.34,0.34
response to host immune response	GO:0052572	1.51E-02	5.87E-02	0.0,0.0	0.34,0.34
positive regulation of natural killer cell proliferation	GO:0032819	1.51E-02	5.87E-02	0.0,0.0	0.34,0.34
CD8-positive, alpha-beta T cell proliferation	GO:0035740	1.51E-02	5.87E-02	0.0,0.0	0.34,0.34
positive regulation of lamellipodium morphogenesis	GO:2000394	1.51E-02	5.87E-02	0.0,0.0	0.34,0.34
positive regulation of metallopeptidase activity	GO:1905050	1.51E-02	5.87E-02	0.0,0.0	0.34,0.34
regulation of myosin-light-chain-phosphatase activity	GO:0035507	1.51E-02	5.87E-02	0.0,0.0	0.34,0.34
cellular response to peptidoglycan	GO:0071224	1.51E-02	5.87E-02	0.0,0.0	0.34,0.34
tetrahydrofolate biosynthetic process	GO:0046654	1.51E-02	5.87E-02	0.0,0.0	0.34,0.34
trachea formation	GO:0060440	1.51E-02	5.87E-02	0.0,0.0	0.34,0.34
negative regulation of histone H3-K9 trimethylation	GO:1900113	1.51E-02	5.87E-02	0.0,0.0	0.34,0.34
ribonucleoside diphosphate catabolic process	GO:0009191	1.51E-02	5.87E-02	0.0,0.0	0.34,0.34
negative regulation of mitochondrial depolarization	GO:0051902	1.51E-02	5.87E-02	0.0,0.0	0.34,0.34
long-chain fatty acid catabolic process	GO:0042758	1.51E-02	5.87E-02	0.0,0.0	0.34,0.34
vesicle transport along actin filament	GO:0030050	1.51E-02	5.87E-02	0.0,0.0	0.34,0.34
positive regulation of protein targeting to mitochondrion	GO:1903955	1.51E-02	5.87E-02	-0.94,-0.95	-0.01,-0.02
regulation of chromosome organization	GO:0033044	1.52E-02	5.88E-02	-0.6,-0.63	0.11,0.1
tissue homeostasis	GO:0001894	1.52E-02	5.89E-02	0.15,0.15	0.4,0.39
regulation of sequence-specific DNA binding	GO:0051090	1.53E-02	5.91E-02	-0.64,-0.64	0.1,0.1

transcription factor activity					
MAPK cascade	GO:0000165	1.54E-02	5.93E-02	-0.67,-0.63	0.09,0.1
inflammatory response	GO:0006954	1.54E-02	5.94E-02	-1.34,-1.22	-0.16,-0.13
fat cell differentiation	GO:0045444	1.57E-02	6.06E-02	-0.87,-0.85	0.01,0.02
bone remodeling	GO:0046849	1.58E-02	6.07E-02	-0.04,0.02	0.32,0.34
cytokine production involved in immune response	GO:0002367	1.58E-02	6.07E-02	-0.35,-0.3	0.21,0.22
single organismal cell-cell adhesion	GO:0016337	1.59E-02	6.08E-02	-1.38,-1.27	-0.18,-0.14
guanosine-containing compound metabolic process	GO:1901068	1.61E-02	6.17E-02	-0.38,-0.35	0.2,0.21
positive regulation of JUN kinase activity	GO:0043507	1.61E-02	6.17E-02	0.48,0.5	0.52,0.52
regulation of leukocyte proliferation	GO:0070663	1.63E-02	6.21E-02	-0.13,-0.13	0.29,0.29
regulation of cell projection organization	GO:0031344	1.64E-02	6.27E-02	-0.95,-1.15	-0.02,-0.1
regulation of MAPK cascade	GO:0043408	1.68E-02	6.39E-02	-0.1,0.15	0.3,0.39
regulation of protein serine/threonine kinase activity	GO:0071900	1.68E-02	6.39E-02	0.32,0.34	0.46,0.46
positive regulation of type I interferon production	GO:0032481	1.68E-02	6.39E-02	-0.38,-0.35	0.2,0.2
gonad development	GO:0008406	1.71E-02	6.39E-02	-0.7,-0.69	0.08,0.07
response to defenses of other organism involved in symbiotic interaction	GO:0052173	1.72E-02	6.39E-02	0.0,0.0	0.34,0.34
regulation of pinocytosis	GO:0048548	1.72E-02	6.39E-02	0.0,0.0	0.34,0.34
regulation of tau-protein kinase activity	GO:1902947	1.72E-02	6.39E-02	0.0,0.0	0.34,0.34
detection of bacterial lipopeptide	GO:0070340	1.72E-02	6.39E-02	0.0,0.0	0.34,0.34
positive regulation of macrophage cytokine production	GO:0060907	1.72E-02	6.39E-02	0.0,0.0	0.34,0.34
mitotic cytokinetic process	GO:1902410	1.72E-02	6.39E-02	0.0,0.0	0.34,0.34
response to host	GO:0075136	1.72E-02	6.39E-02	0.0,0.0	0.34,0.34
DNA damage induced protein phosphorylation	GO:0006975	1.72E-02	6.39E-02	0.0,0.0	0.34,0.34
interleukin-18 production	GO:0032621	1.72E-02	6.39E-02	0.0,0.0	0.34,0.34
negative regulation of beta-amyloid formation	GO:1902430	1.72E-02	6.39E-02	0.0,0.0	0.34,0.34
glycine biosynthetic process	GO:0006545	1.72E-02	6.39E-02	0.0,0.0	0.34,0.34
negative regulation of membrane depolarization	GO:1904180	1.72E-02	6.39E-02	0.0,0.0	0.34,0.34
response to host defenses	GO:0052200	1.72E-02	6.39E-02	0.0,0.0	0.34,0.34

positive regulation of megakaryocyte differentiation	GO:0045654	1.72E-02	6.39E-02	0.0,0.0	0.34,0.34
nucleoside diphosphate catabolic process	GO:0009134	1.72E-02	6.39E-02	0.0,0.0	0.34,0.34
negative regulation of synapse assembly	GO:0051964	1.72E-02	6.39E-02	0.0,0.0	0.34,0.34
dendritic cell cytokine production	GO:0002371	1.72E-02	6.39E-02	0.0,0.0	0.34,0.34
positive regulation of T cell activation	GO:0050870	1.73E-02	6.41E-02	0.34,0.33	0.47,0.46
negative regulation of growth	GO:0045926	1.75E-02	6.47E-02	-0.39,-0.44	0.19,0.17
substrate adhesion-dependent cell spreading	GO:0034446	1.75E-02	6.48E-02	0.11,0.21	0.38,0.41
protein targeting to vacuole	GO:0006623	1.79E-02	6.59E-02	-0.43,-0.34	0.18,0.21
positive regulation of myeloid cell differentiation	GO:0045639	1.79E-02	6.59E-02	-0.46,-0.44	0.17,0.17
development of primary sexual characteristics	GO:0045137	1.81E-02	6.64E-02	-0.7,-0.69	0.08,0.07
phospholipid biosynthetic process	GO:0008654	1.81E-02	6.65E-02	-0.77,-0.68	0.05,0.08
glycerolipid biosynthetic process	GO:0045017	1.82E-02	6.69E-02	-0.77,-0.68	0.05,0.08
nitric oxide metabolic process	GO:0046209	1.83E-02	6.69E-02	-0.34,-0.27	0.21,0.23
positive regulation of homotypic cell-cell adhesion	GO:0034112	1.84E-02	6.75E-02	0.34,0.33	0.47,0.46
neuron development	GO:0048666	1.86E-02	6.79E-02	-1.99,-2.34	-0.4,-0.55
positive regulation of leukocyte chemotaxis	GO:0002690	1.86E-02	6.79E-02	-0.24,-0.13	0.25,0.28
T cell costimulation	GO:0031295	1.86E-02	6.79E-02	0.32,0.26	0.46,0.44
cellular homeostasis	GO:0019725	1.88E-02	6.83E-02	-1.74,-1.67	-0.31,-0.29
negative regulation of molecular function	GO:0044092	1.88E-02	6.83E-02	-1.58,-1.5	-0.25,-0.23
cellular amine metabolic process	GO:0044106	1.88E-02	6.83E-02	-0.57,-0.5	0.13,0.15
tetrapyrrole metabolic process	GO:0033013	1.90E-02	6.83E-02	0.53,0.58	0.54,0.55
regulation of protein targeting to mitochondrion	GO:1903214	1.90E-02	6.83E-02	-0.94,-0.95	-0.01,-0.02
positive regulation of protein serine/threonine kinase activity	GO:0071902	1.90E-02	6.83E-02	0.8,0.79	0.64,0.63
regulation of angiogenesis	GO:0045765	1.92E-02	6.83E-02	-0.37,-0.29	0.2,0.23
positive regulation of leukocyte cell-cell adhesion	GO:1903039	1.92E-02	6.83E-02	0.34,0.33	0.47,0.46
iron ion transport	GO:0006826	1.94E-02	6.83E-02	-0.49,-0.54	0.16,0.13

response to fatty acid	GO:0070542	1.94E-02	6.83E-02	0.02,0.01	0.35,0.34
establishment of protein localization to vacuole	GO:0072666	1.94E-02	6.83E-02	-0.43,-0.34	0.18,0.21
positive regulation of cytokine secretion involved in immune response	GO:0002741	1.94E-02	6.83E-02	0.0,0.0	0.34,0.34
positive regulation of histone phosphorylation	GO:0033129	1.94E-02	6.83E-02	0.0,0.0	0.34,0.34
activation of cysteine-type endopeptidase activity involved in apoptotic process by cytochrome c	GO:0008635	1.94E-02	6.83E-02	0.0,0.0	0.34,0.34
regulation of single stranded viral RNA replication via double stranded DNA intermediate	GO:0045091	1.94E-02	6.83E-02	0.0,0.0	0.34,0.34
G-protein coupled receptor signaling pathway involved in heart process	GO:0086103	1.94E-02	6.83E-02	0.0,0.0	0.34,0.34
outer ear morphogenesis	GO:0042473	1.94E-02	6.83E-02	0.0,0.0	0.34,0.34
ribonucleoside monophosphate catabolic process	GO:0009158	1.94E-02	6.83E-02	0.0,0.0	0.34,0.34
positive regulation of sphingolipid biosynthetic process	GO:0090154	1.94E-02	6.83E-02	0.0,0.0	0.34,0.34
actin filament-based transport	GO:0099515	1.94E-02	6.83E-02	0.0,0.0	0.34,0.34
positive regulation of ceramide biosynthetic process	GO:2000304	1.94E-02	6.83E-02	0.0,0.0	0.34,0.34
purine ribonucleoside monophosphate catabolic process	GO:0009169	1.94E-02	6.83E-02	0.0,0.0	0.34,0.34
regulation of natural killer cell proliferation	GO:0032817	1.94E-02	6.83E-02	0.0,0.0	0.34,0.34
detection of bacterial lipoprotein	GO:0042494	1.94E-02	6.83E-02	0.0,0.0	0.34,0.34
sphingomyelin catabolic process	GO:0006685	1.94E-02	6.83E-02	0.0,0.0	0.34,0.34
purine deoxyribonucleotide catabolic process	GO:0009155	1.94E-02	6.83E-02	0.0,0.0	0.34,0.34
neuron projection morphogenesis	GO:0048812	1.95E-02	6.87E-02	-1.7,-2.01	-0.3,-0.42
monosaccharide metabolic process	GO:0005996	1.96E-02	6.88E-02	-0.58,-0.56	0.12,0.12
actin filament organization	GO:0007015	1.96E-02	6.89E-02	-1.19,-1.1	-0.11,-0.08
positive regulation of cell proliferation	GO:0008284	1.97E-02	6.90E-02	-1.6,-1.66	-0.26,-0.29

reactive nitrogen species metabolic process	GO:2001057	1.97E-02	6.90E-02	-0.34,-0.27	0.21,0.23
lymphocyte costimulation	GO:0031294	1.97E-02	6.90E-02	0.32,0.26	0.46,0.44
regulation of protein stability	GO:0031647	1.98E-02	6.91E-02	0.24,0.16	0.43,0.4
organonitrogen compound biosynthetic process	GO:1901566	1.98E-02	6.92E-02	-3.93,-3.98	-1.13,-1.16
regulation of oxidoreductase activity	GO:0051341	2.01E-02	7.01E-02	-0.27,-0.31	0.24,0.22
multicellular organismal reproductive process	GO:0048609	2.01E-02	7.01E-02	-1.36,-1.22	-0.17,-0.12
carbohydrate derivative catabolic process	GO:1901136	2.02E-02	7.02E-02	-0.54,-0.55	0.14,0.13
negative regulation of actin filament polymerization	GO:0030837	2.05E-02	7.10E-02	-0.49,-0.42	0.16,0.18
ovulation cycle process	GO:0022602	2.05E-02	7.10E-02	-0.55,-0.5	0.13,0.15
JNK cascade	GO:0007254	2.06E-02	7.12E-02	0.25,0.44	0.43,0.5
oxidation-reduction process	GO:0055114	2.06E-02	7.12E-02	-2.86,-2.77	-0.73,-0.71
ion homeostasis	GO:0050801	2.08E-02	7.18E-02	-1.47,-1.54	-0.21,-0.24
purine ribonucleoside triphosphate metabolic process	GO:0009205	2.08E-02	7.18E-02	-0.14,-0.17	0.29,0.27
steroid hormone mediated signaling pathway	GO:0043401	2.12E-02	7.25E-02	0.27,0.24	0.44,0.43
cellular modified amino acid biosynthetic process	GO:0042398	2.12E-02	7.25E-02	-0.52,-0.51	0.14,0.14
cellular response to peptide hormone stimulus	GO:0071375	2.14E-02	7.25E-02	-0.63,-0.71	0.1,0.07
negative regulation of cellular component movement	GO:0051271	2.14E-02	7.25E-02	-0.82,-1.03	0.03,-0.05
regulation of response to stress	GO:0080134	2.14E-02	7.25E-02	-1.65,-1.81	-0.28,-0.35
cardiac neural crest cell differentiation involved in heart development	GO:0061307	2.15E-02	7.25E-02	0.0,0.0	0.34,0.34
xenophagy	GO:0098792	2.15E-02	7.25E-02	0.0,0.0	0.34,0.34
respiratory burst involved in defense response	GO:0002679	2.15E-02	7.25E-02	0.0,0.0	0.34,0.34
regulation of CD8-positive, alpha-beta T cell activation	GO:2001185	2.15E-02	7.25E-02	0.0,0.0	0.34,0.34
negative regulation of feeding behavior	GO:2000252	2.15E-02	7.25E-02	0.0,0.0	0.34,0.34
establishment of epithelial cell apical/basal polarity	GO:0045198	2.15E-02	7.25E-02	0.0,0.0	0.34,0.34

regulation of removal of superoxide radicals	GO:2000121	2.15E-02	7.25E-02	0.0,0.0	0.34,0.34
purine nucleoside monophosphate catabolic process	GO:0009128	2.15E-02	7.25E-02	0.0,0.0	0.34,0.34
positive regulation of protein exit from endoplasmic reticulum	GO:0070863	2.15E-02	7.25E-02	0.0,0.0	0.34,0.34
Bergmann glial cell differentiation	GO:0060020	2.15E-02	7.25E-02	0.0,0.0	0.34,0.34
negative regulation of amyloid precursor protein catabolic process	GO:1902992	2.15E-02	7.25E-02	0.0,0.0	0.34,0.34
cardiac neural crest cell development involved in heart development	GO:0061308	2.15E-02	7.25E-02	0.0,0.0	0.34,0.34
positive regulation of hematopoietic progenitor cell differentiation	GO:1901534	2.15E-02	7.25E-02	0.0,0.0	0.34,0.34
regulation of endocytic recycling	GO:2001135	2.15E-02	7.25E-02	0.0,0.0	0.34,0.34
MyD88-dependent toll-like receptor signaling pathway	GO:0002755	2.16E-02	7.27E-02	-0.35,-0.3	0.21,0.22
animal organ regeneration	GO:0031100	2.16E-02	7.27E-02	-0.43,-0.34	0.18,0.21
ribonucleoside triphosphate metabolic process	GO:0009199	2.16E-02	7.27E-02	-0.14,-0.17	0.29,0.27
chordate embryonic development	GO:0043009	2.17E-02	7.28E-02	0.39,0.38	0.49,0.48
response to virus	GO:0009615	2.18E-02	7.30E-02	-1.33,-1.2	-0.16,-0.12
signal transduction by protein phosphorylation	GO:0023014	2.18E-02	7.31E-02	-0.67,-0.63	0.09,0.1
purine nucleoside triphosphate metabolic process	GO:0009144	2.19E-02	7.33E-02	-0.14,-0.17	0.29,0.27
amine metabolic process	GO:0009308	2.20E-02	7.35E-02	-0.57,-0.5	0.13,0.15
embryonic placenta development	GO:0001892	2.20E-02	7.35E-02	0.05,0.02	0.36,0.34
regulation of gliogenesis	GO:0014013	2.24E-02	7.45E-02	-0.35,-0.28	0.21,0.23
defense response to Gram-positive bacterium	GO:0050830	2.24E-02	7.45E-02	-0.81,-0.76	0.04,0.05
chemokine-mediated signaling pathway	GO:0070098	2.24E-02	7.45E-02	-0.12,-0.02	0.29,0.33
multicellular organism reproduction	GO:0032504	2.26E-02	7.49E-02	-1.36,-1.22	-0.17,-0.12
regulation of JUN kinase activity	GO:0043506	2.28E-02	7.54E-02	0.48,0.5	0.52,0.52
modification of morphology or physiology of other organism involved in symbiotic interaction	GO:0051817	2.28E-02	7.54E-02	-0.92,-0.95	-0.0,-0.02

positive regulation of T cell proliferation	GO:0042102	2.28E-02	7.54E-02	0.21,0.2	0.42,0.41
nucleotide metabolic process	GO:0009117	2.28E-02	7.54E-02	-0.73,-0.73	0.07,0.06
nervous system development	GO:0007399	2.30E-02	7.59E-02	-3.1,-3.37	-0.82,-0.93
protein localization to vacuole	GO:0072665	2.32E-02	7.65E-02	-0.43,-0.34	0.18,0.21
purine ribonucleoside monophosphate metabolic process	GO:0009167	2.34E-02	7.65E-02	-0.14,-0.17	0.29,0.27
negative regulation of cell proliferation	GO:0008285	2.34E-02	7.65E-02	-1.37,-1.11	-0.17,-0.08
purine nucleoside monophosphate metabolic process	GO:0009126	2.36E-02	7.65E-02	-0.14,-0.17	0.29,0.27
regulation of cytokine biosynthetic process	GO:0042035	2.36E-02	7.65E-02	-0.44,-0.34	0.17,0.21
negative regulation of inclusion body assembly	GO:0090084	2.36E-02	7.65E-02	0.0,0.0	0.34,0.34
single stranded viral RNA replication via double stranded DNA intermediate	GO:0039692	2.36E-02	7.65E-02	0.0,0.0	0.34,0.34
response to bacterial lipopeptide	GO:0070339	2.36E-02	7.65E-02	0.0,0.0	0.34,0.34
tetrahydrobiopterin biosynthetic process	GO:0006729	2.36E-02	7.65E-02	0.0,0.0	0.34,0.34
regulation of lamellipodium morphogenesis	GO:2000392	2.36E-02	7.65E-02	0.0,0.0	0.34,0.34
positive regulation of interferon-gamma secretion	GO:1902715	2.36E-02	7.65E-02	0.0,0.0	0.34,0.34
citrulline biosynthetic process	GO:0019240	2.36E-02	7.65E-02	0.0,0.0	0.34,0.34
positive regulation of transcription from RNA polymerase III promoter	GO:0045945	2.36E-02	7.65E-02	0.0,0.0	0.34,0.34
regulation of female gonad development	GO:2000194	2.36E-02	7.65E-02	0.0,0.0	0.34,0.34
regulation of integrin activation	GO:0033623	2.36E-02	7.65E-02	0.0,0.0	0.34,0.34
Golgi ribbon formation	GO:0090161	2.36E-02	7.65E-02	0.0,0.0	0.34,0.34
biosynthetic process	GO:0009058	2.38E-02	7.69E-02	-13.39,-13.32	-4.67,-4.68
nucleoside phosphate metabolic process	GO:0006753	2.40E-02	7.76E-02	-0.73,-0.73	0.07,0.06
positive regulation of sequence-specific DNA binding transcription factor activity	GO:0051091	2.41E-02	7.79E-02	-0.13,-0.13	0.29,0.29
regulation of vasculature development	GO:1901342	2.46E-02	7.93E-02	-0.37,-0.29	0.2,0.23

stress-activated protein kinase signaling cascade	GO:0031098	2.46E-02	7.94E-02	-0.1,0.1	0.3,0.38
metal ion homeostasis	GO:0055065	2.48E-02	7.97E-02	-1.28,-1.4	-0.14,-0.19
reactive oxygen species biosynthetic process	GO:1903409	2.48E-02	7.97E-02	-0.59,-0.51	0.12,0.14
negative regulation of protein polymerization	GO:0032272	2.48E-02	7.97E-02	-0.49,-0.42	0.16,0.18
glucose metabolic process	GO:0006006	2.50E-02	8.02E-02	-0.19,-0.14	0.27,0.28
positive regulation of neurogenesis	GO:0050769	2.53E-02	8.03E-02	-0.66,-0.65	0.09,0.09
gland development	GO:0048732	2.56E-02	8.03E-02	-0.75,-0.64	0.06,0.1
nitrogen compound metabolic process	GO:0006807	2.57E-02	8.03E-02	-22.3,-22.38	-8.0,-8.09
histone H3-K36 demethylation	GO:0070544	2.57E-02	8.03E-02	0.0,0.0	0.34,0.34
negative regulation of platelet aggregation	GO:0090331	2.57E-02	8.03E-02	0.0,0.0	0.34,0.34
positive regulation of transcription from RNA polymerase II promoter in response to endoplasmic reticulum stress	GO:1990440	2.57E-02	8.03E-02	0.0,0.0	0.34,0.34
negative regulation of telomerase activity	GO:0051974	2.57E-02	8.03E-02	0.0,0.0	0.34,0.34
luteinization	GO:0001553	2.57E-02	8.03E-02	0.0,0.0	0.34,0.34
chaperone-mediated autophagy	GO:0061684	2.57E-02	8.03E-02	0.0,0.0	0.34,0.34
surfactant homeostasis	GO:0043129	2.57E-02	8.03E-02	0.0,0.0	0.34,0.34
protein retention in Golgi apparatus	GO:0045053	2.57E-02	8.03E-02	0.0,0.0	0.34,0.34
response to bacterial lipoprotein	GO:0032493	2.57E-02	8.03E-02	0.0,0.0	0.34,0.34
myoblast migration	GO:0051451	2.57E-02	8.03E-02	0.0,0.0	0.34,0.34
response to methylmercury	GO:0051597	2.57E-02	8.03E-02	0.0,0.0	0.34,0.34
positive regulation of podosome assembly	GO:0071803	2.57E-02	8.03E-02	0.0,0.0	0.34,0.34
cellular response to lipoteichoic acid	GO:0071223	2.57E-02	8.03E-02	0.0,0.0	0.34,0.34
response to lipoteichoic acid	GO:0070391	2.57E-02	8.03E-02	0.0,0.0	0.34,0.34
regulation of histone H3-K9 trimethylation	GO:1900112	2.57E-02	8.03E-02	0.0,0.0	0.34,0.34
tetrahydrobiopterin metabolic process	GO:0046146	2.57E-02	8.03E-02	0.0,0.0	0.34,0.34
regulation of Golgi organization	GO:1903358	2.57E-02	8.03E-02	0.0,0.0	0.34,0.34
positive regulation of tumor necrosis factor biosynthetic process	GO:0042535	2.57E-02	8.03E-02	0.0,0.0	0.34,0.34
opsonization	GO:0008228	2.57E-02	8.03E-02	0.0,0.0	0.34,0.34
negative regulation of interleukin-17 production	GO:0032700	2.57E-02	8.03E-02	0.0,0.0	0.34,0.34

positive regulation of myeloid leukocyte cytokine production involved in immune response	GO:0061081	2.57E-02	8.03E-02	0.0,0.0	0.34,0.34
cellular carbohydrate metabolic process	GO:0044262	2.59E-02	8.07E-02	-0.79,-0.74	0.04,0.06
cellular response to fibroblast growth factor stimulus	GO:0044344	2.62E-02	8.14E-02	0.13,0.16	0.39,0.39
lipid biosynthetic process	GO:0008610	2.64E-02	8.22E-02	-1.2,-1.08	-0.11,-0.07
positive regulation of cellular metabolic process	GO:0031325	2.66E-02	8.25E-02	-6.06,-6.14	-1.93,-1.98
cellular response to peptide	GO:1901653	2.69E-02	8.31E-02	-0.63,-0.71	0.1,0.07
regulation of phosphate metabolic process	GO:0019220	2.69E-02	8.31E-02	-2.07,-2.22	-0.43,-0.5
axonogenesis	GO:0007409	2.69E-02	8.31E-02	-1.03,-1.02	-0.05,-0.05
granulocyte chemotaxis	GO:0071621	2.69E-02	8.31E-02	-0.24,-0.13	0.25,0.28
female gonad development	GO:0008585	2.69E-02	8.31E-02	-0.55,-0.5	0.13,0.15
nucleoside triphosphate metabolic process	GO:0009141	2.70E-02	8.34E-02	-0.14,-0.17	0.29,0.27
positive regulation of cell-cell adhesion	GO:0022409	2.71E-02	8.35E-02	0.34,0.33	0.47,0.46
ribonucleoside monophosphate metabolic process	GO:0009161	2.72E-02	8.38E-02	-0.14,-0.17	0.29,0.27
positive regulation of establishment of protein localization to mitochondrion	GO:1903749	2.73E-02	8.41E-02	-0.94,-0.95	-0.01,-0.02
water-soluble vitamin metabolic process	GO:0006767	2.78E-02	8.44E-02	-0.02,-0.02	0.33,0.33
response to fibroblast growth factor	GO:0071774	2.78E-02	8.44E-02	0.13,0.16	0.39,0.39
protein localization to cell junction	GO:1902414	2.79E-02	8.44E-02	0.0,0.0	0.34,0.34
regulation of establishment of endothelial barrier	GO:1903140	2.79E-02	8.44E-02	0.0,0.0	0.34,0.34
regulation of endothelial cell development	GO:1901550	2.79E-02	8.44E-02	0.0,0.0	0.34,0.34
low-density lipoprotein particle remodeling	GO:0034374	2.79E-02	8.44E-02	0.0,0.0	0.34,0.34
regulation of macrophage cytokine production	GO:0010935	2.79E-02	8.44E-02	0.0,0.0	0.34,0.34
protein autoprocessing	GO:0016540	2.79E-02	8.44E-02	0.0,0.0	0.34,0.34
Rap protein signal transduction	GO:0032486	2.79E-02	8.44E-02	0.0,0.0	0.34,0.34
positive regulation of insulin receptor signaling pathway	GO:0046628	2.79E-02	8.44E-02	0.0,0.0	0.34,0.34

nucleoside monophosphate catabolic process	GO:0009125	2.79E-02	8.44E-02	0.0,0.0	0.34,0.34
negative regulation of histone H3-K9 methylation	GO:0051573	2.79E-02	8.44E-02	0.0,0.0	0.34,0.34
regulation of beta-amyloid formation	GO:1902003	2.79E-02	8.44E-02	0.0,0.0	0.34,0.34
positive regulation of inositol phosphate biosynthetic process	GO:0060732	2.79E-02	8.44E-02	0.0,0.0	0.34,0.34
regulation of phosphorus metabolic process	GO:0051174	2.79E-02	8.44E-02	-2.07,-2.22	-0.43,-0.5
mitochondrion organization	GO:0007005	2.80E-02	8.45E-02	0.59,0.47	0.56,0.51
gliogenesis	GO:0042063	2.80E-02	8.46E-02	-0.48,-0.44	0.16,0.17
regulation of homeostatic process	GO:0032844	2.80E-02	8.46E-02	-0.62,-0.64	0.11,0.09
positive regulation of cell differentiation	GO:0045597	2.82E-02	8.49E-02	-1.2,-1.13	-0.11,-0.09
regulation of interleukin-6 production	GO:0032675	2.82E-02	8.49E-02	-0.44,-0.34	0.17,0.21
regulation of leukocyte chemotaxis	GO:0002688	2.86E-02	8.58E-02	-0.24,-0.13	0.25,0.28
cellular response to heat	GO:0034605	2.86E-02	8.58E-02	-0.25,-0.31	0.25,0.22
cytokine biosynthetic process	GO:0042089	2.86E-02	8.58E-02	-0.44,-0.34	0.17,0.21
cellular iron ion homeostasis	GO:0006879	2.86E-02	8.58E-02	-0.23,-0.24	0.25,0.25
negative regulation of homotypic cell-cell adhesion	GO:0034111	2.91E-02	8.68E-02	-0.38,-0.35	0.2,0.21
development of primary female sexual characteristics	GO:0046545	2.91E-02	8.68E-02	-0.55,-0.5	0.13,0.15
toll-like receptor 4 signaling pathway	GO:0034142	2.91E-02	8.68E-02	-0.35,-0.3	0.21,0.22
regulation of lymphocyte activation	GO:0051249	2.92E-02	8.71E-02	0.25,0.25	0.43,0.43
negative regulation of nervous system development	GO:0051961	2.95E-02	8.71E-02	-0.54,-0.47	0.14,0.16
cytokine metabolic process	GO:0042107	2.95E-02	8.71E-02	-0.44,-0.34	0.17,0.21
positive regulation of catalytic activity	GO:0043085	2.97E-02	8.71E-02	-2.38,-2.39	-0.55,-0.56
organic substance biosynthetic process	GO:1901576	2.99E-02	8.71E-02	-13.01,-12.96	-4.53,-4.54
regulation of interferon-gamma secretion	GO:1902713	3.00E-02	8.71E-02	0.0,0.0	0.34,0.34
negative regulation of substrate adhesion-dependent cell spreading	GO:1900025	3.00E-02	8.71E-02	0.0,0.0	0.34,0.34

protein localization to endosome	GO:0036010	3.00E-02	8.71E-02	0.0,0.0	0.34,0.34
regulation of histone phosphorylation	GO:0033127	3.00E-02	8.71E-02	0.0,0.0	0.34,0.34
positive regulation of steroid hormone secretion	GO:2000833	3.00E-02	8.71E-02	0.0,0.0	0.34,0.34
negative regulation of astrocyte differentiation	GO:0048712	3.00E-02	8.71E-02	0.0,0.0	0.34,0.34
positive regulation of interleukin-8 secretion	GO:2000484	3.00E-02	8.71E-02	0.0,0.0	0.34,0.34
response to dopamine	GO:1903350	3.00E-02	8.71E-02	0.0,0.0	0.34,0.34
Golgi inheritance	GO:0048313	3.00E-02	8.71E-02	0.0,0.0	0.34,0.34
establishment of apical/basal cell polarity	GO:0035089	3.00E-02	8.71E-02	0.0,0.0	0.34,0.34
chondroitin sulfate catabolic process	GO:0030207	3.00E-02	8.71E-02	0.0,0.0	0.34,0.34
regulation of metalloenzyme activity	GO:0048552	3.00E-02	8.71E-02	0.0,0.0	0.34,0.34
natural killer cell proliferation	GO:0001787	3.00E-02	8.71E-02	0.0,0.0	0.34,0.34
CDP-diacylglycerol biosynthetic process	GO:0016024	3.00E-02	8.71E-02	0.0,0.0	0.34,0.34
response to peptidoglycan	GO:0032494	3.00E-02	8.71E-02	0.0,0.0	0.34,0.34
regulation of ceramide biosynthetic process	GO:2000303	3.00E-02	8.71E-02	0.0,0.0	0.34,0.34
cellular response to dopamine	GO:1903351	3.00E-02	8.71E-02	0.0,0.0	0.34,0.34
chemical homeostasis within a tissue	GO:0048875	3.00E-02	8.71E-02	0.0,0.0	0.34,0.34
ferric iron import into cell	GO:0097461	3.00E-02	8.71E-02	0.0,0.0	0.34,0.34
water-soluble vitamin biosynthetic process	GO:0042364	3.00E-02	8.71E-02	0.0,0.0	0.34,0.34
iron ion import across plasma membrane	GO:0098711	3.00E-02	8.71E-02	0.0,0.0	0.34,0.34
oxoacid metabolic process	GO:0043436	3.02E-02	8.77E-02	-1.81,-1.82	-0.34,-0.35
ovulation cycle	GO:0042698	3.04E-02	8.81E-02	-0.55,-0.5	0.13,0.15
cognition	GO:0050890	3.05E-02	8.81E-02	-0.6,-0.49	0.12,0.15
regulation of cysteine-type endopeptidase activity	GO:2000116	3.05E-02	8.81E-02	-0.19,-0.14	0.27,0.28
cellular protein complex assembly	GO:0043623	3.07E-02	8.88E-02	-1.13,-1.05	-0.08,-0.06
interleukin-6 production	GO:0032635	3.08E-02	8.91E-02	-0.44,-0.34	0.17,0.21
lymphocyte proliferation	GO:0046651	3.12E-02	9.00E-02	-0.13,-0.13	0.29,0.29
mononuclear cell proliferation	GO:0032943	3.17E-02	9.02E-02	-0.13,-0.13	0.29,0.29
nucleoside monophosphate metabolic process	GO:0009123	3.19E-02	9.02E-02	-0.14,-0.17	0.29,0.27
regulation of protein targeting	GO:1903533	3.20E-02	9.02E-02	-1.15,-1.09	-0.09,-0.08

negative regulation of focal adhesion assembly	GO:0051895	3.21E-02	9.02E-02	0.0,0.0	0.34,0.34
pentose catabolic process	GO:0019323	3.21E-02	9.02E-02	0.0,0.0	0.34,0.34
positive regulation of phosphoprotein phosphatase activity	GO:0032516	3.21E-02	9.02E-02	0.0,0.0	0.34,0.34
purine deoxyribonucleotide metabolic process	GO:0009151	3.21E-02	9.02E-02	0.0,0.0	0.34,0.34
arginine catabolic process	GO:0006527	3.21E-02	9.02E-02	0.0,0.0	0.34,0.34
macrophage cytokine production	GO:0010934	3.21E-02	9.02E-02	0.0,0.0	0.34,0.34
positive regulation of exosomal secretion	GO:1903543	3.21E-02	9.02E-02	0.0,0.0	0.34,0.34
phosphatidylethanolamine biosynthetic process	GO:0006646	3.21E-02	9.02E-02	0.0,0.0	0.34,0.34
negative regulation of bone resorption	GO:0045779	3.21E-02	9.02E-02	0.0,0.0	0.34,0.34
deoxyribonucleoside diphosphate metabolic process	GO:0009186	3.21E-02	9.02E-02	0.0,0.0	0.34,0.34
polarized epithelial cell differentiation	GO:0030859	3.21E-02	9.02E-02	0.0,0.0	0.34,0.34
regulation of inositol phosphate biosynthetic process	GO:0010919	3.21E-02	9.02E-02	0.0,0.0	0.34,0.34
histone mRNA catabolic process	GO:0071044	3.21E-02	9.02E-02	0.0,0.0	0.34,0.34
toxin metabolic process	GO:0009404	3.21E-02	9.02E-02	0.0,0.0	0.34,0.34
nucleobase-containing small molecule catabolic process	GO:0034656	3.21E-02	9.02E-02	0.0,0.0	0.34,0.34
negative regulation of multicellular organism growth	GO:0040015	3.21E-02	9.02E-02	0.0,0.0	0.34,0.34
response to yeast	GO:0001878	3.21E-02	9.02E-02	0.0,0.0	0.34,0.34
ferric iron import	GO:0033216	3.21E-02	9.02E-02	0.0,0.0	0.34,0.34
positive regulation of sodium ion transmembrane transporter activity	GO:2000651	3.21E-02	9.02E-02	0.0,0.0	0.34,0.34
I-kappaB phosphorylation	GO:0007252	3.21E-02	9.02E-02	0.0,0.0	0.34,0.34
regulation of ER to Golgi vesicle-mediated transport	GO:0060628	3.21E-02	9.02E-02	0.0,0.0	0.34,0.34
CDP-diacylglycerol metabolic process	GO:0046341	3.21E-02	9.02E-02	0.0,0.0	0.34,0.34
regulation of catalytic activity	GO:0050790	3.22E-02	9.05E-02	-3.08,-3.18	-0.81,-0.86
endomembrane system organization	GO:0010256	3.23E-02	9.06E-02	-1.02,-1.25	-0.04,-0.14
granulocyte migration	GO:0097530	3.27E-02	9.16E-02	-0.24,-0.13	0.25,0.28
chemical homeostasis	GO:0048878	3.28E-02	9.18E-02	-0.75,-0.69	0.06,0.08

regulation of protein metabolic process	GO:0051246	3.28E-02	9.18E-02	-2.93,-3.01	-0.76,-0.8
positive regulation of endocytosis	GO:0045807	3.36E-02	9.33E-02	-0.51,-0.59	0.15,0.11
regulation of establishment of protein localization to mitochondrion	GO:1903747	3.36E-02	9.33E-02	-0.94,-0.95	-0.01,-0.02
negative regulation of cell differentiation	GO:0045596	3.36E-02	9.33E-02	-1.18,-1.2	-0.1,-0.12
positive regulation of protein metabolic process	GO:0051247	3.36E-02	9.33E-02	-1.85,-1.91	-0.35,-0.38
sex differentiation	GO:0007548	3.38E-02	9.33E-02	-0.7,-0.69	0.08,0.07
epidermal growth factor receptor signaling pathway	GO:0007173	3.38E-02	9.33E-02	-0.19,-0.14	0.27,0.28
regulation of type I interferon production	GO:0032479	3.40E-02	9.33E-02	-0.38,-0.35	0.2,0.2
response to laminar fluid shear stress	GO:0034616	3.42E-02	9.33E-02	0.0,0.0	0.34,0.34
trachea morphogenesis	GO:0060439	3.42E-02	9.33E-02	0.0,0.0	0.34,0.34
negative regulation of phagocytosis	GO:0050765	3.42E-02	9.33E-02	0.0,0.0	0.34,0.34
regulation of cytokine secretion involved in immune response	GO:0002739	3.42E-02	9.33E-02	0.0,0.0	0.34,0.34
regulation of amyloid precursor protein catabolic process	GO:1902991	3.42E-02	9.33E-02	0.0,0.0	0.34,0.34
negative regulation of adherens junction organization	GO:1903392	3.42E-02	9.33E-02	0.0,0.0	0.34,0.34
neuromuscular process controlling posture	GO:0050884	3.42E-02	9.33E-02	0.0,0.0	0.34,0.34
positive regulation of vacuolar transport	GO:1903337	3.42E-02	9.33E-02	0.0,0.0	0.34,0.34
bone mineralization involved in bone maturation	GO:0035630	3.42E-02	9.33E-02	0.0,0.0	0.34,0.34
histone H3-K9 demethylation	GO:0033169	3.42E-02	9.33E-02	0.0,0.0	0.34,0.34
positive regulation of oligodendrocyte differentiation	GO:0048714	3.42E-02	9.33E-02	0.0,0.0	0.34,0.34
detection of molecule of bacterial origin	GO:0032490	3.42E-02	9.33E-02	0.0,0.0	0.34,0.34
regulation of podosome assembly	GO:0071801	3.42E-02	9.33E-02	0.0,0.0	0.34,0.34
induction of positive chemotaxis	GO:0050930	3.42E-02	9.33E-02	0.0,0.0	0.34,0.34
regulation of megakaryocyte differentiation	GO:0045652	3.42E-02	9.33E-02	0.0,0.0	0.34,0.34

establishment of monopolar cell polarity	GO:0061162	3.42E-02	9.33E-02	0.0,0.0	0.34,0.34
signal complex assembly	GO:0007172	3.42E-02	9.33E-02	0.0,0.0	0.34,0.34
diacylglycerol metabolic process	GO:0046339	3.42E-02	9.33E-02	0.0,0.0	0.34,0.34
purine ribonucleoside metabolic process	GO:0046128	3.43E-02	9.36E-02	-0.94,-0.98	-0.01,-0.03
positive regulation of angiogenesis	GO:0045766	3.45E-02	9.39E-02	-0.12,-0.02	0.29,0.33
regulation of myeloid leukocyte differentiation	GO:0002761	3.45E-02	9.39E-02	-0.38,-0.35	0.2,0.21
collagen metabolic process	GO:0032963	3.45E-02	9.39E-02	-0.6,-0.58	0.12,0.12
regulation of neuron death	GO:1901214	3.46E-02	9.39E-02	-0.75,-0.66	0.06,0.09
purine nucleoside metabolic process	GO:0042278	3.49E-02	9.47E-02	-0.94,-0.98	-0.01,-0.03
platelet degranulation	GO:0002576	3.50E-02	9.49E-02	-0.54,-0.51	0.14,0.14
behavior	GO:0007610	3.51E-02	9.51E-02	-0.76,-0.73	0.05,0.06
T cell activation	GO:0042110	3.54E-02	9.57E-02	0.25,0.25	0.43,0.43
type I interferon production	GO:0032606	3.55E-02	9.57E-02	-0.38,-0.35	0.2,0.2
purine ribonucleoside monophosphate biosynthetic process	GO:0009168	3.55E-02	9.57E-02	-0.4,-0.39	0.19,0.19
purine nucleoside monophosphate biosynthetic process	GO:0009127	3.55E-02	9.57E-02	-0.4,-0.39	0.19,0.19
leukocyte proliferation	GO:0070661	3.56E-02	9.59E-02	-0.13,-0.13	0.29,0.29
positive regulation of protein localization to nucleus	GO:1900182	3.59E-02	9.59E-02	0.02,0.01	0.35,0.34
female sex differentiation	GO:0046660	3.59E-02	9.59E-02	-0.55,-0.5	0.13,0.15
hormone-mediated signaling pathway	GO:0009755	3.62E-02	9.59E-02	0.27,0.24	0.44,0.43
nucleobase-containing small molecule metabolic process	GO:0055086	3.63E-02	9.59E-02	-0.73,-0.73	0.07,0.06
positive regulation of nitric-oxide synthase biosynthetic process	GO:0051770	3.63E-02	9.59E-02	0.0,0.0	0.34,0.34
positive regulation of endothelial cell apoptotic process	GO:2000353	3.63E-02	9.59E-02	0.0,0.0	0.34,0.34
amyloid-beta formation	GO:0034205	3.63E-02	9.59E-02	0.0,0.0	0.34,0.34
IMP salvage	GO:0032264	3.63E-02	9.59E-02	0.0,0.0	0.34,0.34
osteoclast development	GO:0036035	3.63E-02	9.59E-02	0.0,0.0	0.34,0.34
central nervous system myelination	GO:0022010	3.63E-02	9.59E-02	0.0,0.0	0.34,0.34
regulation of proton transport	GO:0010155	3.63E-02	9.59E-02	0.0,0.0	0.34,0.34
positive regulation of macrophage chemotaxis	GO:0010759	3.63E-02	9.59E-02	0.0,0.0	0.34,0.34
copper ion import	GO:0015677	3.63E-02	9.59E-02	0.0,0.0	0.34,0.34

positive regulation of actin nucleation	GO:0051127	3.63E-02	9.59E-02	0.0,0.0	0.34,0.34
positive regulation of telomere capping	GO:1904355	3.63E-02	9.59E-02	0.0,0.0	0.34,0.34
negative regulation of anoikis	GO:2000811	3.63E-02	9.59E-02	0.0,0.0	0.34,0.34
sphingomyelin metabolic process	GO:0006684	3.63E-02	9.59E-02	0.0,0.0	0.34,0.34
negative regulation of bone remodeling	GO:0046851	3.63E-02	9.59E-02	0.0,0.0	0.34,0.34
axon ensheathment in central nervous system	GO:0032291	3.63E-02	9.59E-02	0.0,0.0	0.34,0.34
angiotensin-activated signaling pathway	GO:0038166	3.63E-02	9.59E-02	0.0,0.0	0.34,0.34
establishment or maintenance of monopolar cell polarity	GO:0061339	3.63E-02	9.59E-02	0.0,0.0	0.34,0.34
positive regulation of lipid metabolic process	GO:0045834	3.64E-02	9.62E-02	0.07,0.09	0.36,0.37
cellular component morphogenesis	GO:0032989	3.66E-02	9.65E-02	-2.43,-2.7	-0.57,-0.68
circulatory system development	GO:0072359	3.69E-02	9.70E-02	-0.13,-0.03	0.29,0.32
nucleotide catabolic process	GO:0009166	3.69E-02	9.70E-02	-0.38,-0.35	0.2,0.21
apoptotic mitochondrial changes	GO:0008637	3.69E-02	9.70E-02	-0.09,-0.19	0.3,0.27
regulation of protein modification process	GO:0031399	3.69E-02	9.70E-02	-1.86,-2.03	-0.36,-0.43
ear morphogenesis	GO:0042471	3.74E-02	9.80E-02	-0.22,-0.21	0.26,0.26
alpha-beta T cell activation	GO:0046631	3.74E-02	9.80E-02	-0.57,-0.52	0.13,0.14
multicellular organismal macromolecule metabolic process	GO:0044259	3.74E-02	9.80E-02	-0.6,-0.58	0.12,0.12
leukocyte cell-cell adhesion	GO:0007159	3.78E-02	9.85E-02	0.25,0.25	0.43,0.43
positive regulation of intracellular signal transduction	GO:1902533	3.78E-02	9.85E-02	-0.02,0.01	0.33,0.34
response to hydrogen peroxide	GO:0042542	3.79E-02	9.85E-02	0.58,0.57	0.56,0.55
intracellular signal transduction	GO:0035556	3.79E-02	9.85E-02	-5.86,-5.89	-1.85,-1.88
positive regulation of chemotaxis	GO:0050921	3.84E-02	9.85E-02	-0.24,-0.13	0.25,0.28
deadenylation-dependent decapping of nuclear-transcribed mRNA	GO:0000290	3.84E-02	9.85E-02	0.0,0.0	0.34,0.34
negative regulation of release of cytochrome c from mitochondria	GO:0090201	3.84E-02	9.85E-02	0.0,0.0	0.34,0.34

mammary gland epithelial cell differentiation	GO:0060644	3.84E-02	9.85E-02	0.0,0.0	0.34,0.34
folic acid metabolic process	GO:0046655	3.84E-02	9.85E-02	0.0,0.0	0.34,0.34
regulation of exosomal secretion	GO:1903541	3.84E-02	9.85E-02	0.0,0.0	0.34,0.34
regulation of inclusion body assembly	GO:0090083	3.84E-02	9.85E-02	0.0,0.0	0.34,0.34
positive regulation of nitric-oxide synthase activity	GO:0051000	3.84E-02	9.85E-02	0.0,0.0	0.34,0.34
Golgi vesicle budding	GO:0048194	3.84E-02	9.85E-02	0.0,0.0	0.34,0.34
regulation of immunoglobulin secretion	GO:0051023	3.84E-02	9.85E-02	0.0,0.0	0.34,0.34
GDP metabolic process	GO:0046710	3.84E-02	9.85E-02	0.0,0.0	0.34,0.34
regulation of telomerase RNA localization to Cajal body	GO:1904872	3.84E-02	9.85E-02	0.0,0.0	0.34,0.34
lamellipodium morphogenesis	GO:0072673	3.84E-02	9.85E-02	0.0,0.0	0.34,0.34
phosphatidylethanolamine metabolic process	GO:0046337	3.84E-02	9.85E-02	0.0,0.0	0.34,0.34
regulation of sphingolipid biosynthetic process	GO:0090153	3.84E-02	9.85E-02	0.0,0.0	0.34,0.34
histone H3-K9 trimethylation	GO:0036124	3.84E-02	9.85E-02	0.0,0.0	0.34,0.34
regulation of membrane lipid metabolic process	GO:1905038	3.84E-02	9.85E-02	0.0,0.0	0.34,0.34
negative regulation of interleukin-12 production	GO:0032695	3.84E-02	9.85E-02	0.0,0.0	0.34,0.34
positive regulation of nervous system development	GO:0051962	3.84E-02	9.86E-02	-0.66,-0.65	0.09,0.09
innate immune response activating cell surface receptor signaling pathway	GO:0002220	3.88E-02	9.93E-02	0.02,0.01	0.35,0.34
negative regulation of DNA metabolic process	GO:0051053	3.88E-02	9.93E-02	-0.04,0.06	0.32,0.36
gland morphogenesis	GO:0022612	3.88E-02	9.93E-02	0.04,0.06	0.35,0.36
monocarboxylic acid catabolic process	GO:0072329	3.88E-02	9.93E-02	-0.27,-0.31	0.24,0.22
aging	GO:0007568	3.92E-02	1.00E-01	-1.03,-0.95	-0.05,-0.02
actin cytoskeleton reorganization	GO:0031532	3.93E-02	1.00E-01	-0.4,-0.61	0.19,0.11
mucopolysaccharide metabolic process	GO:1903510	3.93E-02	1.00E-01	-0.29,-0.34	0.23,0.21
positive regulation of phosphorus metabolic process	GO:0010562	3.95E-02	1.00E-01	-0.67,-0.63	0.09,0.1

positive regulation of phosphate metabolic process	GO:0045937	3.95E-02	1.00E-01	-0.67,-0.63	0.09,0.1
negative regulation of signal transduction	GO:0009968	3.96E-02	1.01E-01	4.17,4.06	1.9,1.86
cell projection assembly	GO:0030031	3.96E-02	1.01E-01	-0.57,-0.38	0.12,0.19
negative regulation of cysteine-type endopeptidase activity	GO:2000117	3.98E-02	1.01E-01	0.09,0.16	0.37,0.4
integrin activation	GO:0033622	4.05E-02	1.02E-01	0.0,0.0	0.34,0.34
RNA localization to Cajal body	GO:0090670	4.05E-02	1.02E-01	0.0,0.0	0.34,0.34
telomerase RNA localization to Cajal body	GO:0090671	4.05E-02	1.02E-01	0.0,0.0	0.34,0.34
organelle inheritance	GO:0048308	4.05E-02	1.02E-01	0.0,0.0	0.34,0.34
CD8-positive, alpha-beta T cell activation	GO:0036037	4.05E-02	1.02E-01	0.0,0.0	0.34,0.34
telomerase RNA localization	GO:0090672	4.05E-02	1.02E-01	0.0,0.0	0.34,0.34
regulation of interleukin-6 biosynthetic process	GO:0045408	4.05E-02	1.02E-01	0.0,0.0	0.34,0.34
negative regulation of cell development	GO:0010721	4.06E-02	1.02E-01	-0.63,-0.67	0.1,0.08
nucleoside phosphate catabolic process	GO:1901292	4.08E-02	1.03E-01	-0.38,-0.35	0.2,0.21
protein phosphorylation	GO:0006468	4.12E-02	1.03E-01	-4.84,-5.01	-1.47,-1.55
regulation of protein kinase activity	GO:0045859	4.12E-02	1.03E-01	-0.19,-0.14	0.27,0.28
positive regulation of cell projection organization	GO:0031346	4.15E-02	1.04E-01	-0.19,-0.14	0.27,0.28
intrinsic apoptotic signaling pathway	GO:0097193	4.15E-02	1.04E-01	-0.19,-0.14	0.27,0.28
ribonucleoside metabolic process	GO:0009119	4.17E-02	1.05E-01	-0.94,-0.98	-0.01,-0.03
regulation of protein polymerization	GO:0032271	4.18E-02	1.05E-01	-0.64,-0.58	0.1,0.12
positive regulation of cellular amide metabolic process	GO:0034250	4.18E-02	1.05E-01	-0.39,-0.39	0.19,0.19
positive regulation of canonical Wnt signaling pathway	GO:0090263	4.18E-02	1.05E-01	0.54,0.51	0.54,0.53
purine ribonucleotide metabolic process	GO:0009150	4.21E-02	1.05E-01	-0.53,-0.57	0.14,0.12
cellular process involved in reproduction in multicellular organism	GO:0022412	4.21E-02	1.05E-01	-0.19,-0.14	0.27,0.28
macromolecule catabolic process	GO:0009057	4.23E-02	1.05E-01	-1.98,-2.01	-0.4,-0.42
long-chain fatty acid metabolic process	GO:0001676	4.23E-02	1.05E-01	-0.27,-0.3	0.24,0.22
protein dephosphorylation	GO:0006470	4.24E-02	1.05E-01	-0.63,-0.7	0.1,0.07

regulation of tumor necrosis factor biosynthetic process	GO:0042534	4.25E-02	1.05E-01	0.0,0.0	0.34,0.34
epithelial cell proliferation involved in liver morphogenesis	GO:0072575	4.25E-02	1.05E-01	0.0,0.0	0.34,0.34
myelin assembly	GO:0032288	4.25E-02	1.05E-01	0.0,0.0	0.34,0.34
purine nucleotide salvage	GO:0032261	4.25E-02	1.05E-01	0.0,0.0	0.34,0.34
cellular response to platelet-derived growth factor stimulus	GO:0036120	4.25E-02	1.05E-01	0.0,0.0	0.34,0.34
hepatocyte proliferation	GO:0072574	4.25E-02	1.05E-01	0.0,0.0	0.34,0.34
negative regulation of cell junction assembly	GO:1901889	4.25E-02	1.05E-01	0.0,0.0	0.34,0.34
citrulline metabolic process	GO:0000052	4.25E-02	1.05E-01	0.0,0.0	0.34,0.34
positive regulation of transcription elongation from RNA polymerase II promoter	GO:0032968	4.25E-02	1.05E-01	0.0,0.0	0.34,0.34
tumor necrosis factor biosynthetic process	GO:0042533	4.25E-02	1.05E-01	0.0,0.0	0.34,0.34
regulation of platelet aggregation	GO:0090330	4.25E-02	1.05E-01	0.0,0.0	0.34,0.34
negative regulation of platelet activation	GO:0010544	4.25E-02	1.05E-01	0.0,0.0	0.34,0.34
protein oligomerization	GO:0051259	4.28E-02	1.05E-01	-0.89,-0.76	0.0,0.05
skeletal system development	GO:0001501	4.28E-02	1.05E-01	-0.82,-0.7	0.03,0.07
epithelium development	GO:0060429	4.29E-02	1.05E-01	-0.71,-0.68	0.07,0.08
regulation of endopeptidase activity	GO:0052548	4.30E-02	1.06E-01	-0.88,-0.84	0.01,0.02
cellular protein modification process	GO:0006464	4.31E-02	1.06E-01	-7.94,-8.12	-2.63,-2.72
protein modification process	GO:0036211	4.31E-02	1.06E-01	-7.94,-8.12	-2.63,-2.72
regulation of exocytosis	GO:0017157	4.33E-02	1.06E-01	-0.65,-0.64	0.1,0.1
positive regulation of vasculature development	GO:1904018	4.34E-02	1.06E-01	-0.12,-0.02	0.29,0.33
positive regulation of lymphocyte proliferation	GO:0050671	4.39E-02	1.07E-01	0.21,0.2	0.42,0.41
response to ammonium ion	GO:0060359	4.39E-02	1.07E-01	-0.36,-0.37	0.21,0.2
regulation of cellular amide metabolic process	GO:0034248	4.41E-02	1.07E-01	-0.7,-0.7	0.08,0.07
negative regulation of hydrolase activity	GO:0051346	4.41E-02	1.07E-01	-0.82,-0.81	0.03,0.03
cellular response to type I interferon	GO:0071357	4.44E-02	1.07E-01	-0.55,-0.45	0.13,0.17
type I interferon signaling pathway	GO:0060337	4.44E-02	1.07E-01	-0.55,-0.45	0.13,0.17
positive regulation of mononuclear cell proliferation	GO:0032946	4.44E-02	1.07E-01	0.21,0.2	0.42,0.41

cellular modified amino acid metabolic process	GO:0006575	4.45E-02	1.07E-01	-0.7,-0.67	0.08,0.08
interleukin-6 biosynthetic process	GO:0042226	4.46E-02	1.07E-01	0.0,0.0	0.34,0.34
PERK-mediated unfolded protein response	GO:0036499	4.46E-02	1.07E-01	0.0,0.0	0.34,0.34
exosomal secretion	GO:1990182	4.46E-02	1.07E-01	0.0,0.0	0.34,0.34
transcytosis	GO:0045056	4.46E-02	1.07E-01	0.0,0.0	0.34,0.34
cellular response to fluid shear stress	GO:0071498	4.46E-02	1.07E-01	0.0,0.0	0.34,0.34
phosphatidylserine acyl-chain remodeling	GO:0036150	4.46E-02	1.07E-01	0.0,0.0	0.34,0.34
planar cell polarity pathway involved in neural tube closure	GO:0090179	4.46E-02	1.07E-01	0.0,0.0	0.34,0.34
regulation of nitric-oxide synthase biosynthetic process	GO:0051769	4.46E-02	1.07E-01	0.0,0.0	0.34,0.34
peroxisome proliferator activated receptor signaling pathway	GO:0035357	4.46E-02	1.07E-01	0.0,0.0	0.34,0.34
positive regulation of sodium ion transmembrane transport	GO:1902307	4.46E-02	1.07E-01	0.0,0.0	0.34,0.34
negative regulation of histone methylation	GO:0031061	4.46E-02	1.07E-01	0.0,0.0	0.34,0.34
microglial cell activation	GO:0001774	4.46E-02	1.07E-01	0.0,0.0	0.34,0.34
response to platelet-derived growth factor	GO:0036119	4.46E-02	1.07E-01	0.0,0.0	0.34,0.34
regulation of protein exit from endoplasmic reticulum	GO:0070861	4.46E-02	1.07E-01	0.0,0.0	0.34,0.34
regulation of transcription involved in cell fate commitment	GO:0060850	4.46E-02	1.07E-01	0.0,0.0	0.34,0.34
positive regulation of toll-like receptor signaling pathway	GO:0034123	4.46E-02	1.07E-01	0.0,0.0	0.34,0.34
nitric-oxide synthase biosynthetic process	GO:0051767	4.46E-02	1.07E-01	0.0,0.0	0.34,0.34
cation homeostasis	GO:0055080	4.47E-02	1.07E-01	-1.28,-1.4	-0.14,-0.19
ribonucleotide metabolic process	GO:0009259	4.51E-02	1.08E-01	-0.53,-0.57	0.14,0.12
carboxylic acid catabolic process	GO:0046395	4.51E-02	1.08E-01	-0.67,-0.69	0.09,0.07
organic acid catabolic process	GO:0016054	4.51E-02	1.08E-01	-0.67,-0.69	0.09,0.07
neuron death	GO:0070997	4.54E-02	1.08E-01	-0.75,-0.66	0.06,0.09
icosanoid metabolic process	GO:0006690	4.59E-02	1.09E-01	-0.35,-0.3	0.21,0.22
regulation of regulated secretory pathway	GO:1903305	4.59E-02	1.09E-01	-0.39,-0.41	0.19,0.18

nucleobase-containing compound catabolic process	GO:0034655	4.63E-02	1.09E-01	-1.12,-1.16	-0.08,-0.1
response to type I interferon	GO:0034340	4.65E-02	1.09E-01	-0.55,-0.45	0.13,0.17
positive regulation of cell development	GO:0010720	4.66E-02	1.09E-01	-0.66,-0.65	0.09,0.09
innate immune response-activating signal transduction	GO:0002758	4.66E-02	1.09E-01	-0.19,-0.14	0.27,0.28
hydrogen peroxide catabolic process	GO:0042744	4.67E-02	1.09E-01	0.0,0.0	0.34,0.34
pinocytosis	GO:0006907	4.67E-02	1.09E-01	0.0,0.0	0.34,0.34
NLS-bearing protein import into nucleus	GO:0006607	4.67E-02	1.09E-01	0.0,0.0	0.34,0.34
beta-catenin destruction complex disassembly	GO:1904886	4.67E-02	1.09E-01	0.0,0.0	0.34,0.34
response to acidic pH	GO:0010447	4.67E-02	1.09E-01	0.0,0.0	0.34,0.34
regulation of establishment of planar polarity involved in neural tube closure	GO:0090178	4.67E-02	1.09E-01	0.0,0.0	0.34,0.34
sphingosine-1-phosphate signaling pathway	GO:0003376	4.67E-02	1.09E-01	0.0,0.0	0.34,0.34
liver morphogenesis	GO:0072576	4.67E-02	1.09E-01	0.0,0.0	0.34,0.34
immunoglobulin secretion	GO:0048305	4.67E-02	1.09E-01	0.0,0.0	0.34,0.34
positive regulation of protein processing	GO:0010954	4.67E-02	1.09E-01	0.0,0.0	0.34,0.34
interferon-gamma secretion	GO:0072643	4.67E-02	1.09E-01	0.0,0.0	0.34,0.34
iron ion import into cell	GO:0097459	4.67E-02	1.09E-01	0.0,0.0	0.34,0.34
cobalamin metabolic process	GO:0009235	4.67E-02	1.09E-01	0.0,0.0	0.34,0.34
cellular response to angiotensin	GO:1904385	4.67E-02	1.09E-01	0.0,0.0	0.34,0.34
muscle hypertrophy in response to stress	GO:0003299	4.67E-02	1.09E-01	0.0,0.0	0.34,0.34
cardiac muscle hypertrophy in response to stress	GO:0014898	4.67E-02	1.09E-01	0.0,0.0	0.34,0.34
protein processing	GO:0016485	4.68E-02	1.09E-01	2.1,2.15	1.12,1.15
macroautophagy	GO:0016236	4.70E-02	1.10E-01	-0.73,-0.65	0.07,0.09
positive regulation of leukocyte proliferation	GO:0070665	4.75E-02	1.11E-01	0.21,0.2	0.42,0.41
ribonucleoside monophosphate biosynthetic process	GO:0009156	4.75E-02	1.11E-01	-0.4,-0.39	0.19,0.19
response to metal ion	GO:0010038	4.76E-02	1.11E-01	-0.39,-0.44	0.19,0.17
single organism reproductive process	GO:0044702	4.79E-02	1.11E-01	-1.54,-1.39	-0.24,-0.19
intracellular steroid hormone receptor signaling pathway	GO:0030518	4.81E-02	1.12E-01	2.59,2.52	1.31,1.29

negative regulation of cellular protein metabolic process	GO:0032269	4.85E-02	1.12E-01	-1.23,-1.23	-0.12,-0.13
integrin-mediated signaling pathway	GO:0007229	4.86E-02	1.12E-01	0.16,0.2	0.4,0.41
mammary gland development	GO:0030879	4.86E-02	1.12E-01	0.04,0.06	0.35,0.36
multicellular organism metabolic process	GO:0044236	4.86E-02	1.12E-01	-0.6,-0.58	0.12,0.12
positive regulation of protein maturation	GO:1903319	4.88E-02	1.12E-01	0.0,0.0	0.34,0.34
regulation of macrophage chemotaxis	GO:0010758	4.88E-02	1.12E-01	0.0,0.0	0.34,0.34
ossification involved in bone maturation	GO:0043931	4.88E-02	1.12E-01	0.0,0.0	0.34,0.34
regulation of mitochondrial depolarization	GO:0051900	4.88E-02	1.12E-01	0.0,0.0	0.34,0.34
establishment of planar polarity involved in neural tube closure	GO:0090177	4.88E-02	1.12E-01	0.0,0.0	0.34,0.34
regulation of mononuclear cell migration	GO:0071675	4.88E-02	1.12E-01	0.0,0.0	0.34,0.34
muscle cell cellular homeostasis	GO:0046716	4.88E-02	1.12E-01	0.0,0.0	0.34,0.34
negative regulation of tissue remodeling	GO:0034104	4.88E-02	1.12E-01	0.0,0.0	0.34,0.34
regulation of telomere capping	GO:1904353	4.88E-02	1.12E-01	0.0,0.0	0.34,0.34
positive regulation of natural killer cell activation	GO:0032816	4.88E-02	1.12E-01	0.0,0.0	0.34,0.34
inositol phosphate biosynthetic process	GO:0032958	4.88E-02	1.12E-01	0.0,0.0	0.34,0.34
regulation of interleukin-8 secretion	GO:2000482	4.88E-02	1.12E-01	0.0,0.0	0.34,0.34
cardiac muscle adaptation	GO:0014887	4.88E-02	1.12E-01	0.0,0.0	0.34,0.34
labyrinthine layer blood vessel development	GO:0060716	4.88E-02	1.12E-01	0.0,0.0	0.34,0.34
response to hypoxia	GO:0001666	4.88E-02	1.12E-01	-0.19,-0.14	0.27,0.28
activation of innate immune response	GO:0002218	4.88E-02	1.12E-01	-0.19,-0.14	0.27,0.28
cellular transition metal ion homeostasis	GO:0046916	4.91E-02	1.12E-01	-0.23,-0.24	0.25,0.25
purine nucleotide metabolic process	GO:0006163	4.93E-02	1.12E-01	-0.53,-0.57	0.14,0.12
hormone secretion	GO:0046879	4.94E-02	1.13E-01	-1.3,-1.36	-0.15,-0.18
regulation of mitochondrion organization	GO:0010821	4.98E-02	1.13E-01	-0.39,-0.44	0.19,0.17
cell projection organization	GO:0030030	4.98E-02	1.13E-01	-2.07,-2.42	-0.44,-0.57

Table S3. All Enriched Cell component.

GO_Name	GO_ID	Pvalue	Pvalue_adjusted
secretory vesicle	GO:0099503	7.25E-13	1.86E-10
secretory granule	GO:0030141	1.06E-12	1.86E-10
cytoplasm	GO:0005737	1.42E-11	1.64E-09
intracellular vesicle	GO:0097708	1.87E-11	1.64E-09
cytoplasmic vesicle	GO:0031410	7.34E-11	5.17E-09
cytoplasmic part	GO:0044444	2.52E-10	1.48E-08
membrane-bounded vesicle	GO:0031988	4.36E-10	2.08E-08
endomembrane system	GO:0012505	4.73E-10	2.08E-08
lysosome	GO:0005764	8.08E-10	3.16E-08
lytic vacuole	GO:0000323	9.16E-10	3.22E-08
primary lysosome	GO:0005766	2.54E-09	7.46E-08
azurophil granule	GO:0042582	2.54E-09	7.46E-08
vesicle	GO:0031982	4.01E-09	1.09E-07
cytoplasmic, membrane-bounded vesicle	GO:0016023	5.48E-09	1.38E-07
vacuole	GO:0005773	1.65E-08	3.87E-07
cytoplasmic vesicle part	GO:0044433	2.69E-08	5.92E-07
extracellular membrane-bounded organelle	GO:0065010	2.88E-08	5.96E-07
extracellular organelle	GO:0043230	3.77E-08	7.37E-07
secretory granule lumen	GO:0034774	4.36E-08	8.09E-07
azurophil granule lumen	GO:0035578	5.46E-08	9.62E-07
cytoplasmic membrane-bounded vesicle lumen	GO:0060205	6.61E-08	1.09E-06
vesicle lumen	GO:0031983	6.78E-08	1.09E-06
vacuolar part	GO:0044437	8.82E-08	1.35E-06
vacuolar lumen	GO:0005775	1.14E-07	1.67E-06
extracellular region part	GO:0044421	1.42E-07	2.00E-06
extracellular exosome	GO:0070062	1.55E-07	2.11E-06
extracellular vesicle	GO:1903561	1.71E-07	2.22E-06
intracellular organelle part	GO:0044446	2.33E-07	2.93E-06
extracellular region	GO:0005576	5.45E-07	6.43E-06
organelle part	GO:0044422	5.48E-07	6.43E-06
membrane-bounded organelle	GO:0043227	6.38E-07	7.25E-06
organelle	GO:0043226	9.59E-07	1.06E-05
cytosol	GO:0005829	9.28E-06	9.90E-05
secretory granule membrane	GO:0030667	9.78E-06	1.01E-04
intracellular organelle	GO:0043229	2.10E-05	2.11E-04
intracellular membrane-bounded organelle	GO:0043231	2.55E-05	2.50E-04
intracellular part	GO:0044424	2.90E-05	2.76E-04
organelle lumen	GO:0043233	3.00E-05	2.78E-04
membrane-enclosed lumen	GO:0031974	3.22E-05	2.91E-04
nuclear envelope	GO:0005635	4.66E-05	4.10E-04
intracellular organelle lumen	GO:0070013	5.24E-05	4.50E-04
organelle membrane	GO:0031090	8.20E-05	6.73E-04
bounding membrane of organelle	GO:0098588	8.22E-05	6.73E-04
intracellular	GO:0005622	8.94E-05	7.15E-04
focal adhesion	GO:0005925	1.04E-04	8.11E-04
cell-substrate adherens junction	GO:0005924	1.12E-04	8.56E-04
cell-substrate junction	GO:0030055	1.21E-04	9.03E-04
nuclear envelope lumen	GO:0005641	1.35E-04	9.93E-04
endosome	GO:0005768	2.91E-04	2.09E-03

lytic vacuole membrane	GO:0098852	3.04E-04	2.14E-03
ficolin-1-rich granule membrane	GO:0101003	3.48E-04	2.40E-03
secondary lysosome	GO:0005767	4.36E-04	2.95E-03
lysosomal membrane	GO:0005765	4.48E-04	2.98E-03
late endosome	GO:0005770	4.57E-04	2.98E-03
actomyosin	GO:0042641	6.13E-04	3.92E-03
phagocytic vesicle	GO:0045335	6.44E-04	4.01E-03
trans-Golgi network	GO:0005802	6.77E-04	4.08E-03
membrane microdomain	GO:0098857	6.87E-04	4.08E-03
adherens junction	GO:0005912	6.95E-04	4.08E-03
anchoring junction	GO:0070161	8.77E-04	5.06E-03
caveola	GO:0005901	9.18E-04	5.21E-03
whole membrane	GO:0098805	1.12E-03	6.24E-03
mitochondrion	GO:0005739	1.41E-03	7.74E-03
plasma membrane raft	GO:0044853	1.47E-03	7.95E-03
cell surface	GO:0009986	1.55E-03	8.25E-03
cell part	GO:0044464	1.62E-03	8.45E-03
extracellular space	GO:0005615	1.63E-03	8.45E-03
organelle envelope	GO:0031967	1.67E-03	8.50E-03
envelope	GO:0031975	1.70E-03	8.53E-03
Golgi cisterna	GO:0031985	1.73E-03	8.60E-03
vacuolar membrane	GO:0005774	1.90E-03	9.28E-03
endocytic vesicle	GO:0030139	2.01E-03	9.67E-03
isoamylase complex	GO:0043033	2.22E-03	1.04E-02
chaperone-mediated autophagy translocation complex	GO:0061742	2.22E-03	1.04E-02
podosome	GO:0002102	2.46E-03	1.14E-02
cell	GO:0005623	2.50E-03	1.14E-02
ficolin-1-rich granule	GO:0101002	2.71E-03	1.21E-02
ficolin-1-rich granule lumen	GO:1904813	2.71E-03	1.21E-02
actin cytoskeleton	GO:0015629	3.00E-03	1.32E-02
organelle subcompartment	GO:0031984	3.28E-03	1.43E-02
Golgi subcompartment	GO:0098791	3.55E-03	1.52E-02
phagocytic vesicle membrane	GO:0030670	3.81E-03	1.62E-02
Golgi stack	GO:0005795	4.12E-03	1.73E-02
Toll-like receptor 2-Toll-like receptor 6 protein complex	GO:0035355	4.44E-03	1.77E-02
intrinsic component of autophagosome membrane	GO:0097636	4.44E-03	1.77E-02
Toll-like receptor 1-Toll-like receptor 2 protein complex	GO:0035354	4.44E-03	1.77E-02
integral component of autophagosome membrane	GO:0097637	4.44E-03	1.77E-02
cytoplasmic vesicle membrane	GO:0030659	4.60E-03	1.82E-02
vesicle membrane	GO:0012506	5.16E-03	2.02E-02
tertiary granule	GO:0070820	5.91E-03	2.29E-02
external side of plasma membrane	GO:0009897	6.18E-03	2.37E-02
mitochondrial matrix	GO:0005759	6.45E-03	2.44E-02
CHOP-C/EBP complex	GO:0036488	6.65E-03	2.49E-02
membrane raft	GO:0045121	7.15E-03	2.65E-02
contractile actin filament bundle	GO:0097517	7.44E-03	2.67E-02
microtubule organizing center attachment site	GO:0034992	7.44E-03	2.67E-02
LINC complex	GO:0034993	7.44E-03	2.67E-02
azurophil granule membrane	GO:0035577	7.69E-03	2.73E-02

plasma membrane part	GO:0044459	8.24E-03	2.90E-02
Golgi membrane	GO:0000139	8.37E-03	2.92E-02
stress fiber	GO:0001725	8.46E-03	2.92E-02
intrinsic component of vacuolar membrane	GO:0031310	8.85E-03	2.97E-02
integral component of vacuolar membrane	GO:0031166	8.85E-03	2.97E-02
histone locus body	GO:0035363	8.85E-03	2.97E-02
Golgi apparatus	GO:0005794	9.29E-03	3.09E-02
cell periphery	GO:0071944	9.44E-03	3.11E-02
side of membrane	GO:0098552	1.00E-02	3.26E-02
integral component of plasma membrane	GO:0005887	1.07E-02	3.42E-02
actin filament bundle	GO:0032432	1.07E-02	3.42E-02
Golgi apparatus part	GO:0044431	1.10E-02	3.47E-02
phagolysosome	GO:0032010	1.11E-02	3.47E-02
platelet dense granule membrane	GO:0031088	1.33E-02	4.09E-02
sperm plasma membrane	GO:0097524	1.33E-02	4.09E-02
cell junction	GO:0030054	1.35E-02	4.12E-02
intrinsic component of plasma membrane	GO:0031226	1.43E-02	4.34E-02
endosomal part	GO:0044440	1.53E-02	4.49E-02
multivesicular body lumen	GO:0097486	1.54E-02	4.49E-02
Golgi cis cisterna	GO:0000137	1.54E-02	4.49E-02
late endosome lumen	GO:0031906	1.54E-02	4.49E-02
PTW/PP1 phosphatase complex	GO:0072357	1.54E-02	4.49E-02
vacuolar proton-transporting V-type ATPase, V0 domain	GO:0000220	1.76E-02	5.01E-02
intracellular non-membrane-bounded organelle	GO:0043232	1.77E-02	5.01E-02
non-membrane-bounded organelle	GO:0043228	1.77E-02	5.01E-02
lysosomal lumen	GO:0043202	1.79E-02	5.01E-02
lipid particle	GO:0005811	1.79E-02	5.01E-02
autolysosome	GO:0044754	1.98E-02	5.45E-02
alveolar lamellar body	GO:0097208	1.98E-02	5.45E-02
chromosome, centromeric region	GO:0000775	2.08E-02	5.67E-02
organelle envelope lumen	GO:0031970	2.14E-02	5.79E-02
endocytic vesicle membrane	GO:0030666	2.16E-02	5.81E-02
alpha DNA polymerase:primase complex	GO:0005658	2.20E-02	5.86E-02
actin filament	GO:0005884	2.30E-02	6.08E-02
plasma membrane	GO:0005886	2.42E-02	6.36E-02
brush border	GO:0005903	2.59E-02	6.75E-02
nuclear part	GO:0044428	2.80E-02	7.25E-02
signal peptidase complex	GO:0005787	2.85E-02	7.32E-02
acrosomal vesicle	GO:0001669	2.94E-02	7.50E-02
NADPH oxidase complex	GO:0043020	3.06E-02	7.76E-02
mitochondrial part	GO:0044429	3.18E-02	7.99E-02
nuclear inner membrane	GO:0005637	3.45E-02	8.62E-02
low-density lipoprotein particle	GO:0034362	3.50E-02	8.66E-02
extrinsic component of plasma membrane	GO:0019897	3.63E-02	8.94E-02
lamellar body	GO:0042599	4.14E-02	1.01E-01
RNAi effector complex	GO:0031332	4.35E-02	1.05E-01
RISC complex	GO:0016442	4.35E-02	1.05E-01
chromatin	GO:0000785	4.38E-02	1.05E-01
endosome membrane	GO:0010008	4.52E-02	1.07E-01
platelet dense granule	GO:0042827	4.56E-02	1.07E-01

nucleotide-activated protein kinase complex	GO:0031588	4.56E-02	1.07E-01
chromosomal part	GO:0044427	4.69E-02	1.09E-01
pericentric heterochromatin	GO:0005721	4.77E-02	1.09E-01
transcriptionally active chromatin	GO:0035327	4.77E-02	1.09E-01
Z disc	GO:0030018	4.79E-02	1.09E-01

Table S4. All Enriched Molecular function.

GO_Name	GO_ID	Pvalue	Pvalue_adjusted
protein binding	GO:0005515	2.07E-11	9.90E-09
enzyme binding	GO:0019899	1.93E-07	4.62E-05
binding	GO:0005488	1.04E-05	1.66E-03
phosphoprotein binding	GO:0051219	4.75E-05	5.67E-03
carboxylic acid binding	GO:0031406	5.50E-04	3.14E-02
organic acid binding	GO:0043177	5.59E-04	3.14E-02
nuclear hormone receptor binding	GO:0035257	7.78E-04	3.14E-02
receptor binding	GO:0005102	1.06E-03	3.14E-02
lipid binding	GO:0008289	1.07E-03	3.14E-02
steroid hormone receptor binding	GO:0035258	1.07E-03	3.14E-02
modified amino acid binding	GO:0072341	1.25E-03	3.14E-02
histone acetyltransferase binding	GO:0035035	1.57E-03	3.14E-02
hydrolase activity, acting on ester bonds	GO:0016788	1.78E-03	3.14E-02
ubiquitin-like protein ligase binding	GO:0044389	1.97E-03	3.14E-02
lipopolysaccharide binding	GO:0001530	2.07E-03	3.14E-02
hormone receptor binding	GO:0051427	2.08E-03	3.14E-02
sterol-4-alpha-carboxylate 3-dehydrogenase (decarboxylating) activity	GO:0047012	2.16E-03	3.14E-02
phosphocholine phosphatase activity	GO:0052731	2.16E-03	3.14E-02
bis(5'-nucleosyl)-tetraphosphatase (symmetrical) activity	GO:0008803	2.16E-03	3.14E-02
C-3 sterol dehydrogenase (C-4 sterol decarboxylase) activity	GO:0000252	2.16E-03	3.14E-02
arginine deiminase activity	GO:0016990	2.16E-03	3.14E-02
triacyl lipopeptide binding	GO:0042497	2.16E-03	3.14E-02
amylo-alpha-1,6-glucosidase activity	GO:0004135	2.16E-03	3.14E-02
cob(I)yrinic acid a,c-diamide adenosyltransferase activity	GO:0008817	2.16E-03	3.14E-02
8-hydroxy-dADP phosphatase activity	GO:0044717	2.16E-03	3.14E-02
iron chaperone activity	GO:0034986	2.16E-03	3.14E-02
8-oxo-GDP phosphatase activity	GO:0044716	2.16E-03	3.14E-02
glycogen debranching enzyme activity	GO:0004133	2.16E-03	3.14E-02
4-alpha-glucanotransferase activity	GO:0004134	2.16E-03	3.14E-02
folate reductase activity	GO:0033560	2.16E-03	3.14E-02
xylulokinase activity	GO:0004856	2.16E-03	3.14E-02
phosphoethanolamine phosphatase activity	GO:0052732	2.16E-03	3.14E-02
hormone-sensitive lipase activity	GO:0033878	2.16E-03	3.14E-02
lipase activity	GO:0016298	2.50E-03	3.52E-02

catalytic activity	GO:0003824	2.79E-03	3.81E-02
protease binding	GO:0002020	3.27E-03	4.34E-02
phosphatidylserine binding	GO:0001786	3.43E-03	4.41E-02
triglyceride lipase activity	GO:0004806	3.60E-03	4.41E-02
oxidoreductase activity, oxidizing metal ions	GO:0016722	3.60E-03	4.41E-02
estrogen receptor binding	GO:0030331	4.13E-03	4.49E-02
bis(5'-nucleosyl)-tetraphosphatase activity	GO:0008796	4.32E-03	4.49E-02
8-oxo-dGDP phosphatase activity	GO:0044715	4.32E-03	4.49E-02
N-acetylgalactosamine-4-sulfatase activity	GO:0003943	4.32E-03	4.49E-02
diacyl lipopeptide binding	GO:0042498	4.32E-03	4.49E-02
acyloxyacyl hydrolase activity	GO:0050528	4.32E-03	4.49E-02
bis(5'-nucleosyl)-tetraphosphatase (asymmetrical) activity	GO:0004081	4.32E-03	4.49E-02
scaffold protein binding	GO:0097110	5.94E-03	5.84E-02
hydrolase activity	GO:0016787	6.28E-03	5.84E-02
CD40 receptor binding	GO:0005174	6.48E-03	5.84E-02
methotrexate binding	GO:0051870	6.48E-03	5.84E-02
insulin-like growth factor-activated receptor activity	GO:0005010	6.48E-03	5.84E-02
interleukin-8 receptor binding	GO:0005153	6.48E-03	5.84E-02
5'-3' exoribonuclease activity	GO:0004534	6.48E-03	5.84E-02
protein-arginine omega-N symmetric methyltransferase activity	GO:0035243	8.63E-03	7.24E-02
N-acetyllactosamine synthase activity	GO:0003945	8.63E-03	7.24E-02
ribonuclease A activity	GO:0004522	8.63E-03	7.24E-02
dihydrofolate reductase activity	GO:0004146	8.63E-03	7.24E-02
actin filament binding	GO:0051015	9.05E-03	7.46E-02
magnesium ion binding	GO:0000287	1.04E-02	8.45E-02
lipopolysaccharide receptor activity	GO:0001875	1.08E-02	8.47E-02
protein C-terminus binding	GO:0008022	1.08E-02	8.47E-02
monocarboxylic acid binding	GO:0033293	1.13E-02	8.72E-02
NADP binding	GO:0050661	1.19E-02	8.99E-02
transcription coactivator activity	GO:0003713	1.20E-02	8.99E-02
ion binding	GO:0043167	1.25E-02	9.08E-02
protein-arginine deiminase activity	GO:0004668	1.29E-02	9.08E-02
lipoteichoic acid binding	GO:0070891	1.29E-02	9.08E-02
alpha-glucosidase activity	GO:0090599	1.29E-02	9.08E-02
connexin binding	GO:0071253	1.51E-02	1.04E-01
oxidoreductase activity	GO:0016491	1.55E-02	1.06E-01
actin binding	GO:0003779	1.58E-02	1.06E-01
carboxylic ester hydrolase activity	GO:0052689	1.70E-02	1.08E-01
beta-N-acetylglucosaminylglycopeptide beta-1,4-galactosyltransferase activity	GO:0003831	1.72E-02	1.08E-01
sequence-specific mRNA binding	GO:1990825	1.72E-02	1.08E-01
arginine binding	GO:0034618	1.72E-02	1.08E-01

metallochaperone activity	GO:0016530	1.72E-02	1.08E-01
amide binding	GO:0033218	1.90E-02	1.18E-01
sphingomyelin phosphodiesterase activity	GO:0004767	1.93E-02	1.18E-01
phospholipid binding	GO:0005543	2.09E-02	1.26E-01
metal ion binding	GO:0046872	2.14E-02	1.26E-01
lipopeptide binding	GO:0071723	2.14E-02	1.26E-01
lipid transporter activity	GO:0005319	2.16E-02	1.26E-01
Rac GTPase binding	GO:0048365	2.31E-02	1.28E-01
non-membrane spanning protein tyrosine kinase activity	GO:0004715	2.35E-02	1.28E-01
lipoprotein lipase activity	GO:0004465	2.36E-02	1.28E-01
histone demethylase activity (H3-K36 specific)	GO:0051864	2.36E-02	1.28E-01
Toll-like receptor binding	GO:0035325	2.36E-02	1.28E-01
m7G(5')pppN diphosphatase activity	GO:0050072	2.36E-02	1.28E-01
glycosaminoglycan binding	GO:0005539	2.53E-02	1.31E-01
oxidoreductase activity, oxidizing metal ions, oxygen as acceptor	GO:0016724	2.57E-02	1.31E-01
ferroxidase activity	GO:0004322	2.57E-02	1.31E-01
cation binding	GO:0043169	2.65E-02	1.31E-01
sphingolipid transporter activity	GO:0046624	2.78E-02	1.31E-01
peptidoglycan binding	GO:0042834	2.78E-02	1.31E-01
adenyl-nucleotide exchange factor activity	GO:0000774	2.78E-02	1.31E-01
cupric reductase activity	GO:0008823	2.78E-02	1.31E-01
ferric-chelate reductase (NADPH) activity	GO:0052851	2.78E-02	1.31E-01
arylsulfatase activity	GO:0004065	2.78E-02	1.31E-01
3-beta-hydroxy-delta5-steroid dehydrogenase activity	GO:0003854	2.78E-02	1.31E-01
C-C chemokine binding	GO:0019957	2.78E-02	1.31E-01
small GTPase binding	GO:0031267	2.79E-02	1.31E-01
ion channel binding	GO:0044325	2.80E-02	1.31E-01
amino acid binding	GO:0016597	2.85E-02	1.32E-01
kinase binding	GO:0019900	2.87E-02	1.32E-01
molecular transducer activity	GO:0060089	2.90E-02	1.32E-01
NADPH binding	GO:0070402	2.99E-02	1.34E-01
translation repressor activity, nucleic acid binding	GO:0000900	2.99E-02	1.34E-01
acylglycerol lipase activity	GO:0047372	3.20E-02	1.34E-01
nucleoside-diphosphatase activity	GO:0017110	3.20E-02	1.34E-01
low-density lipoprotein particle binding	GO:0030169	3.20E-02	1.34E-01
histone demethylase activity (H3-K9 specific)	GO:0032454	3.20E-02	1.34E-01
adenosine-phosphate deaminase activity	GO:0047623	3.20E-02	1.34E-01
superoxide-generating NADPH oxidase activator activity	GO:0016176	3.20E-02	1.34E-01
AMP deaminase activity	GO:0003876	3.20E-02	1.34E-01

intracellular calcium activated chloride channel activity	GO:0005229	3.41E-02	1.38E-01
low-density lipoprotein receptor activity	GO:0005041	3.41E-02	1.38E-01
glucocorticoid receptor binding	GO:0035259	3.41E-02	1.38E-01
integrin binding	GO:0005178	3.43E-02	1.38E-01
anion binding	GO:0043168	3.52E-02	1.38E-01
GTPase binding	GO:0051020	3.53E-02	1.38E-01
N,N-dimethylaniline monooxygenase activity	GO:0004499	3.62E-02	1.38E-01
signaling pattern recognition receptor activity	GO:0008329	3.62E-02	1.38E-01
pattern recognition receptor activity	GO:0038187	3.62E-02	1.38E-01
transmembrane receptor protein phosphatase activity	GO:0019198	3.62E-02	1.38E-01
transmembrane receptor protein tyrosine phosphatase activity	GO:0005001	3.62E-02	1.38E-01
phospholipid transporter activity	GO:0005548	3.81E-02	1.41E-01
MHC class I protein binding	GO:0042288	3.83E-02	1.41E-01
glucosidase activity	GO:0015926	3.83E-02	1.41E-01
C-C chemokine receptor activity	GO:0016493	3.83E-02	1.41E-01
superoxide-generating NADPH oxidase activity	GO:0016175	3.83E-02	1.41E-01
1-acylglycerol-3-phosphate O-acyltransferase activity	GO:0003841	4.03E-02	1.45E-01
CXCR chemokine receptor binding	GO:0045236	4.03E-02	1.45E-01
hydrolase activity, acting on carbon-nitrogen (but not peptide) bonds, in linear amidines	GO:0016813	4.03E-02	1.45E-01
cytoskeletal protein binding	GO:0008092	4.11E-02	1.47E-01
lipoprotein particle receptor activity	GO:0030228	4.24E-02	1.50E-01
lysophosphatidic acid acyltransferase activity	GO:0042171	4.45E-02	1.55E-01
lysophospholipid acyltransferase activity	GO:0071617	4.45E-02	1.55E-01
neurexin family protein binding	GO:0042043	4.66E-02	1.57E-01
ferric iron binding	GO:0008199	4.66E-02	1.57E-01
insulin-like growth factor II binding	GO:0031995	4.66E-02	1.57E-01
nucleotide diphosphatase activity	GO:0004551	4.66E-02	1.57E-01
telomerase RNA binding	GO:0070034	4.66E-02	1.57E-01
phosphoric ester hydrolase activity	GO:0042578	4.71E-02	1.57E-01

Table S5. All Enriched KEGG pathway.

Pathway Name	Pathway ID	Pvalue	Pvalue_adjusted	Count	Pop Hit
Tuberculosis	hsa05152	1.04E-04	8.44E-03	7	179
Bladder cancer	hsa05219	1.13E-04	8.44E-03	4	41
Lysosome	hsa04142	9.35E-04	4.67E-02	5	123
Regulation of actin cytoskeleton	hsa04810	2.00E-03	6.52E-02	6	215
Rheumatoid arthritis	hsa05323	2.37E-03	6.52E-02	4	91
Phagosome	hsa04145	2.61E-03	6.52E-02	5	155
Malaria	hsa05144	3.41E-03	7.31E-02	3	49

Legionellosis	hsa05134	4.73E-03	8.88E-02	3	55
Shigellosis	hsa05131	7.55E-03	1.17E-01	3	65
Viral carcinogenesis	hsa05203	8.52E-03	1.17E-01	5	205
Epithelial cell signaling in Helicobacter pylori infection	hsa05120	8.55E-03	1.17E-01	3	68
Adherens junction	hsa04520	1.08E-02	1.20E-01	3	74
Leishmaniasis	hsa05140	1.08E-02	1.20E-01	3	74
Pertussis	hsa05133	1.12E-02	1.20E-01	3	75
Hepatitis B	hsa05161	1.26E-02	1.26E-01	4	146
Axon guidance	hsa04360	2.34E-02	2.03E-01	4	176
Amoebiasis	hsa05146	2.40E-02	2.03E-01	3	100
Chagas disease (American trypanosomiasis)	hsa05142	2.66E-02	2.03E-01	3	104
Porphyrin and chlorophyll metabolism	hsa00860	2.79E-02	2.03E-01	2	42
Toll-like receptor signaling pathway	hsa04620	2.79E-02	2.03E-01	3	106
Chemokine signaling pathway	hsa04062	2.84E-02	2.03E-01	4	187
TNF signaling pathway	hsa04668	3.07E-02	2.10E-01	3	110
Metabolic pathways	hsa01100	3.47E-02	2.11E-01	13	1239
Leukocyte transendothelial migration	hsa04670	3.67E-02	2.11E-01	3	118
Focal adhesion	hsa04510	3.68E-02	2.11E-01	4	203
Toxoplasmosis	hsa05145	3.75E-02	2.11E-01	3	119
Proteoglycans in cancer	hsa05205	3.80E-02	2.11E-01	4	205
Platelet activation	hsa04611	3.99E-02	2.14E-01	3	122
NOD-like receptor signaling pathway	hsa04621	4.88E-02	2.53E-01	2	57
VEGF signaling pathway	hsa04370	5.51E-02	2.76E-01	2	61
Phospholipase D signaling pathway	hsa04072	6.01E-02	2.91E-01	3	144
Prolactin signaling pathway	hsa04917	7.38E-02	3.30E-01	2	72
PPAR signaling pathway	hsa03320	7.38E-02	3.30E-01	2	72
Oxytocin signaling pathway	hsa04921	7.49E-02	3.30E-01	3	158
Folate biosynthesis	hsa00790	8.39E-02	3.60E-01	1	14
Glycosaminoglycan biosynthesis - keratan sulfate	hsa00533	8.96E-02	3.69E-01	1	15
Purine metabolism	hsa00230	9.62E-02	3.69E-01	3	176
Salmonella infection	hsa05132	1.00E-01	3.69E-01	2	86
Transcriptional misregulation in cancer	hsa05202	1.01E-01	3.69E-01	3	180
Gap junction	hsa04540	1.04E-01	3.69E-01	2	88
ErbB signaling pathway	hsa04012	1.04E-01	3.69E-01	2	88
GnRH signaling pathway	hsa04912	1.10E-01	3.69E-01	2	91
Glycosaminoglycan degradation	hsa00531	1.12E-01	3.69E-01	1	19
Fc gamma R-mediated phagocytosis	hsa04666	1.14E-01	3.69E-01	2	93

NF-kappa B signaling pathway	hsa04064	1.14E-01	3.69E-01	2	93
One carbon pool by folate	hsa00670	1.18E-01	3.69E-01	1	20
Steroid biosynthesis	hsa00100	1.18E-01	3.69E-01	1	20
Glycerophospholipid metabolism	hsa00564	1.18E-01	3.69E-01	2	95
Estrogen signaling pathway	hsa04915	1.26E-01	3.76E-01	2	99
cAMP signaling pathway	hsa04024	1.26E-01	3.76E-01	3	199
Melanogenesis	hsa04916	1.28E-01	3.76E-01	2	100
AGE-RAGE signaling pathway in diabetic complications	hsa04933	1.30E-01	3.76E-01	2	101
Protein export	hsa03060	1.34E-01	3.79E-01	1	23
T cell receptor signaling pathway	hsa04660	1.39E-01	3.79E-01	2	105
Pyrimidine metabolism	hsa00240	1.39E-01	3.79E-01	2	105
Glycosphingolipid biosynthesis - lacto and neolacto series	hsa00601	1.50E-01	4.03E-01	1	26
Collecting duct acid secretion	hsa04966	1.56E-01	4.10E-01	1	27
Dorso-ventral axis formation	hsa04320	1.61E-01	4.16E-01	1	28
Thyroid cancer	hsa05216	1.66E-01	4.18E-01	1	29
Thyroid hormone signaling pathway	hsa04919	1.67E-01	4.18E-01	2	118
Vascular smooth muscle contraction	hsa04270	1.72E-01	4.22E-01	2	120
Asthma	hsa05310	1.82E-01	4.28E-01	1	32
Apoptosis - multiple species	hsa04215	1.87E-01	4.28E-01	1	33
Prion diseases	hsa05020	1.97E-01	4.28E-01	1	35
Osteoclast differentiation	hsa04380	1.99E-01	4.28E-01	2	132
Hepatitis C	hsa05160	2.01E-01	4.28E-01	2	133
Pentose and glucuronate interconversions	hsa00040	2.02E-01	4.28E-01	1	36
DNA replication	hsa03030	2.02E-01	4.28E-01	1	36
Natural killer cell mediated cytotoxicity	hsa04650	2.06E-01	4.28E-01	2	135
Primary immunodeficiency	hsa05340	2.07E-01	4.28E-01	1	37
Systemic lupus erythematosus	hsa05322	2.08E-01	4.28E-01	2	136
Insulin signaling pathway	hsa04910	2.15E-01	4.28E-01	2	139
Tight junction	hsa04530	2.15E-01	4.28E-01	2	139
Apoptosis	hsa04210	2.17E-01	4.28E-01	2	140
Allograft rejection	hsa05330	2.17E-01	4.28E-01	1	39
Aldosterone-regulated sodium reabsorption	hsa04960	2.17E-01	4.28E-01	1	39
Signaling pathways regulating pluripotency of stem cells	hsa04550	2.22E-01	4.32E-01	2	142

Cytokine-cytokine receptor interaction	hsa04060	2.28E-01	4.38E-01	3	265
Cell adhesion molecules (CAMs)	hsa04514	2.31E-01	4.38E-01	2	146
Pathways in cancer	hsa05200	2.33E-01	4.38E-01	4	397
Non-alcoholic fatty liver disease (NAFLD)	hsa04932	2.42E-01	4.49E-01	2	151
Sphingolipid metabolism	hsa00600	2.55E-01	4.64E-01	1	47
Notch signaling pathway	hsa04330	2.60E-01	4.64E-01	1	48
Type II diabetes mellitus	hsa04930	2.60E-01	4.64E-01	1	48
Intestinal immune network for IgA production	hsa04672	2.69E-01	4.70E-01	1	50
Vibrio cholerae infection	hsa05110	2.74E-01	4.70E-01	1	51
Endometrial cancer	hsa05213	2.78E-01	4.70E-01	1	52
cGMP-PKG signaling pathway	hsa04022	2.80E-01	4.70E-01	2	167
Alzheimer's disease	hsa05010	2.82E-01	4.70E-01	2	168
Autoimmune thyroid disease	hsa05320	2.88E-01	4.70E-01	1	54
Basal cell carcinoma	hsa05217	2.92E-01	4.70E-01	1	55
Regulation of lipolysis in adipocytes	hsa04923	2.96E-01	4.70E-01	1	56
Non-small cell lung cancer	hsa05223	2.96E-01	4.70E-01	1	56
Influenza A	hsa05164	3.01E-01	4.70E-01	2	176
Starch and sucrose metabolism	hsa00500	3.01E-01	4.70E-01	1	57
Acute myeloid leukemia	hsa05221	3.01E-01	4.70E-01	1	57
Glycerolipid metabolism	hsa00561	3.10E-01	4.71E-01	1	59
Viral myocarditis	hsa05416	3.14E-01	4.71E-01	1	60
mTOR signaling pathway	hsa04150	3.14E-01	4.71E-01	1	60
Long-term depression	hsa04730	3.14E-01	4.71E-01	1	60
Colorectal cancer	hsa05210	3.23E-01	4.77E-01	1	62
Synaptic vesicle cycle	hsa04721	3.27E-01	4.77E-01	1	63
Glioma	hsa05214	3.35E-01	4.77E-01	1	65
Long-term potentiation	hsa04720	3.39E-01	4.77E-01	1	66
Pancreatic cancer	hsa05212	3.39E-01	4.77E-01	1	66
Inflammatory bowel disease (IBD)	hsa05321	3.39E-01	4.77E-01	1	66
Renal cell carcinoma	hsa05211	3.44E-01	4.77E-01	1	67
Central carbon metabolism in cancer	hsa05230	3.44E-01	4.77E-01	1	67
Fc epsilon RI signaling pathway	hsa04664	3.48E-01	4.79E-01	1	68
Drug metabolism - cytochrome P450	hsa00982	3.52E-01	4.80E-01	1	69
RIG-I-like receptor signaling pathway	hsa04622	3.56E-01	4.81E-01	1	70
Melanoma	hsa05218	3.60E-01	4.82E-01	1	71
Chronic myeloid leukemia	hsa05220	3.68E-01	4.84E-01	1	73
B cell receptor signaling pathway	hsa04662	3.68E-01	4.84E-01	1	73

Arrhythmogenic right ventricular cardiomyopathy (ARVC)	hsa05412	3.72E-01	4.85E-01	1	74
Rap1 signaling pathway	hsa04015	3.81E-01	4.92E-01	2	211
RNA degradation	hsa03018	3.84E-01	4.92E-01	1	77
Bacterial invasion of epithelial cells	hsa05100	3.88E-01	4.93E-01	1	78
Complement and coagulation cascades	hsa04610	3.92E-01	4.94E-01	1	79
Aldosterone synthesis and secretion	hsa04925	3.99E-01	4.99E-01	1	81
TGF-beta signaling pathway	hsa04350	4.11E-01	5.09E-01	1	84
GABAergic synapse	hsa04727	4.25E-01	5.23E-01	1	88
Prostate cancer	hsa05215	4.29E-01	5.23E-01	1	89
Circadian entrainment	hsa04713	4.50E-01	5.45E-01	1	95
Progesterone-mediated oocyte maturation	hsa04914	4.61E-01	5.48E-01	1	98
Inflammatory mediator regulation of TRP channels	hsa04750	4.61E-01	5.48E-01	1	98
Retrograde endocannabinoid signaling	hsa04723	4.71E-01	5.52E-01	1	101
Choline metabolism in cancer	hsa05231	4.71E-01	5.52E-01	1	101
HIF-1 signaling pathway	hsa04066	4.77E-01	5.55E-01	1	103
Endocytosis	hsa04144	4.86E-01	5.61E-01	2	260
Cholinergic synapse	hsa04725	5.03E-01	5.75E-01	1	111
Serotonergic synapse	hsa04726	5.06E-01	5.75E-01	1	112
Glutamatergic synapse	hsa04724	5.13E-01	5.78E-01	1	114
Neurotrophin signaling pathway	hsa04722	5.31E-01	5.93E-01	1	120
Sphingolipid signaling pathway	hsa04071	5.34E-01	5.93E-01	1	121
Oocyte meiosis	hsa04114	5.40E-01	5.95E-01	1	123
AMPK signaling pathway	hsa04152	5.46E-01	5.97E-01	1	125
Oxidative phosphorylation	hsa00190	5.68E-01	6.16E-01	1	133
FoxO signaling pathway	hsa04068	5.71E-01	6.16E-01	1	134
Measles	hsa05162	5.76E-01	6.18E-01	1	136
Wnt signaling pathway	hsa04310	5.95E-01	6.33E-01	1	143
Adrenergic signaling in cardiomyocytes	hsa04261	6.10E-01	6.44E-01	1	149
Hippo signaling pathway	hsa04390	6.22E-01	6.53E-01	1	154
PI3K-Akt signaling pathway	hsa04151	6.37E-01	6.63E-01	2	342
Protein processing in endoplasmic reticulum	hsa04141	6.50E-01	6.73E-01	1	166
Alcoholism	hsa05034	6.78E-01	6.97E-01	1	179
Herpes simplex infection	hsa05168	6.92E-01	7.07E-01	1	186
Ras signaling pathway	hsa04014	7.65E-01	7.76E-01	1	228
MAPK signaling pathway	hsa04010	8.03E-01	8.08E-01	1	255
HTLV-I infection	hsa05166	8.08E-01	8.08E-01	1	259

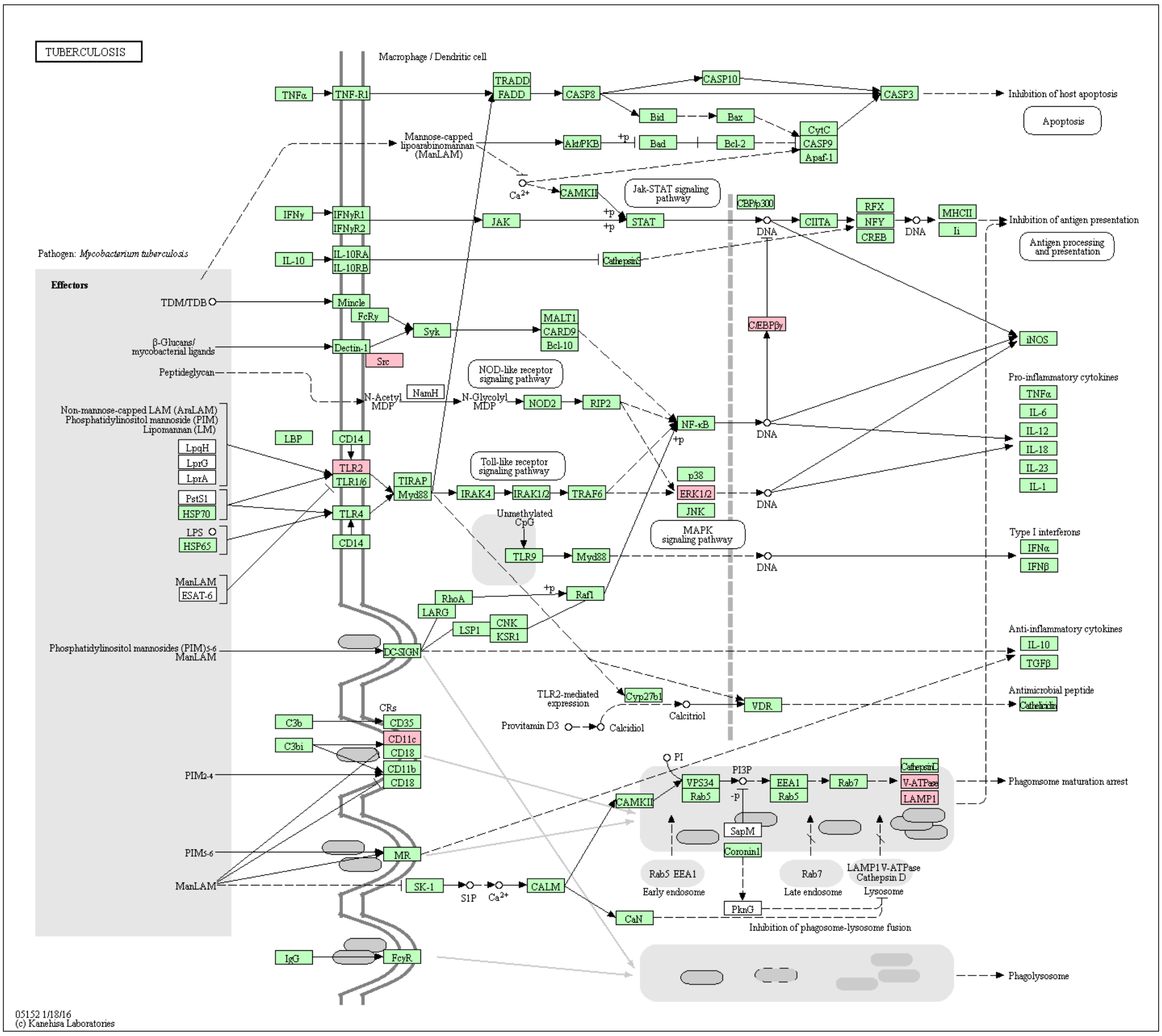

Figure S2. Overlapping differential proteins in KEGG pathway of Tuberculosis.

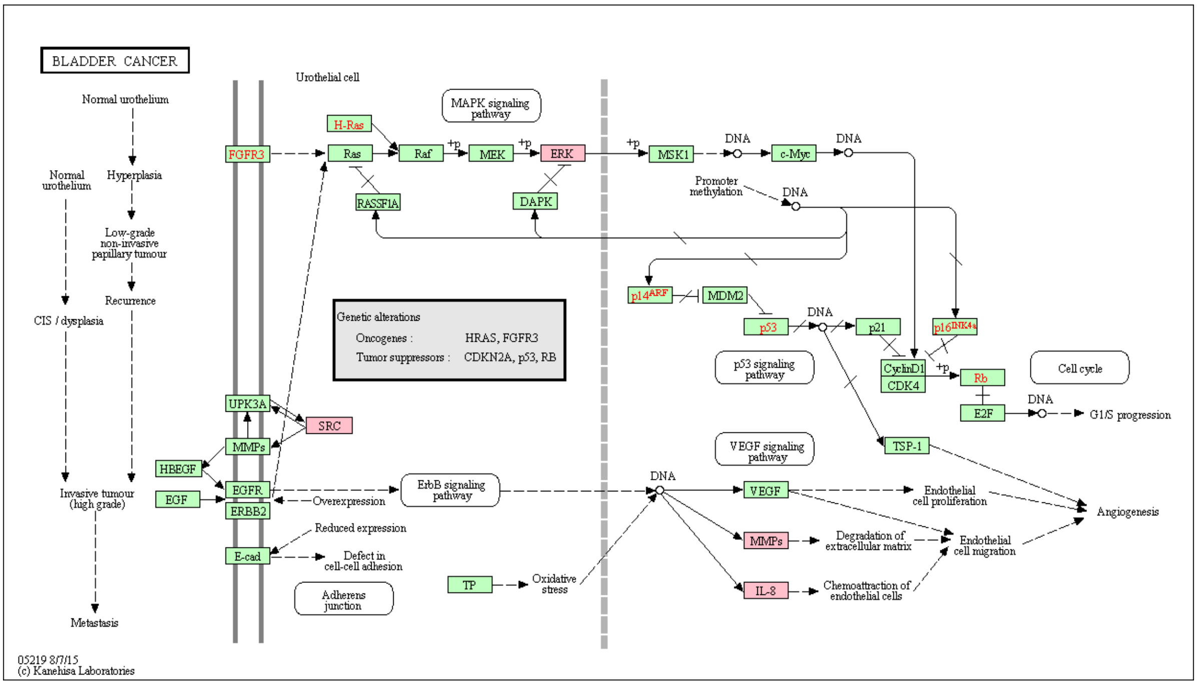

Figure S3. Overlapping differential proteins in KEGG pathway of Bladder cancer.

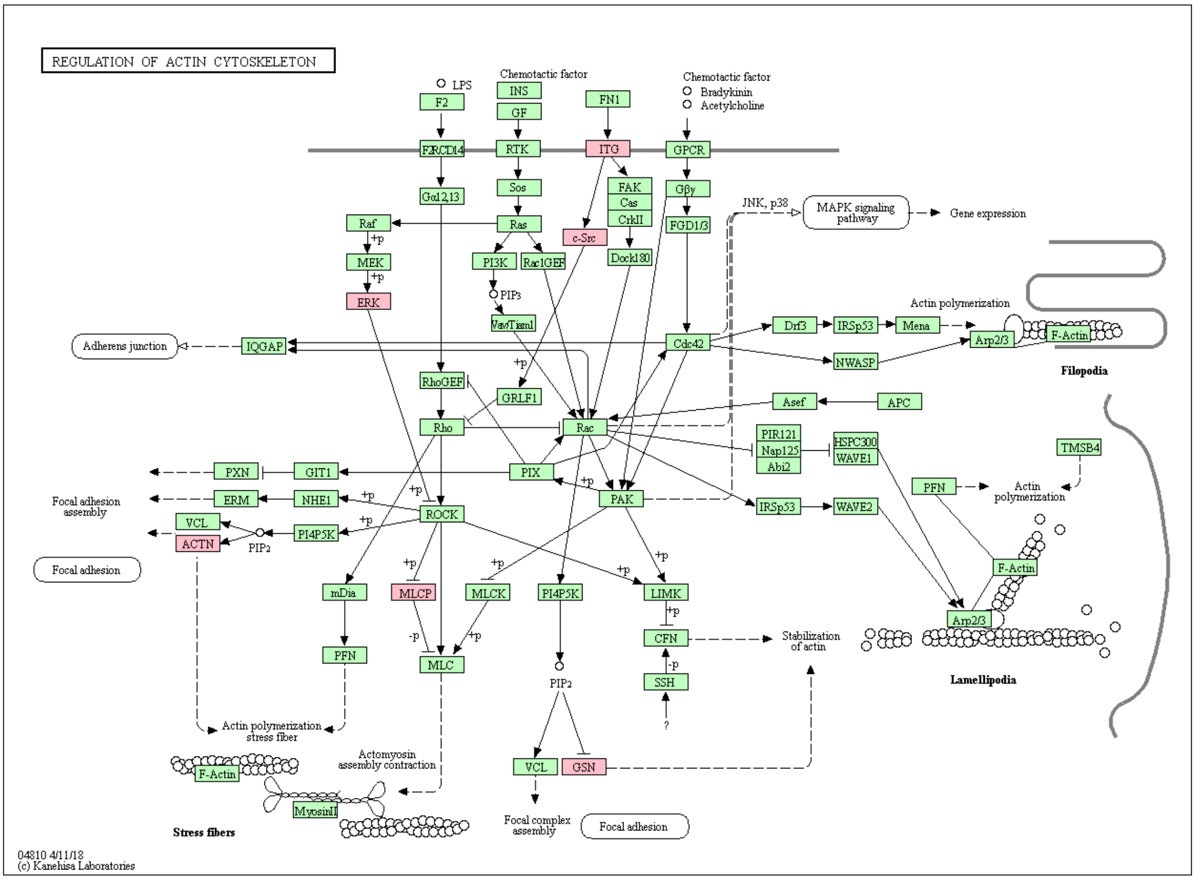

Figure S4. Overlapping differential proteins in KEGG pathway of Regulation of actin cytoskeleton.

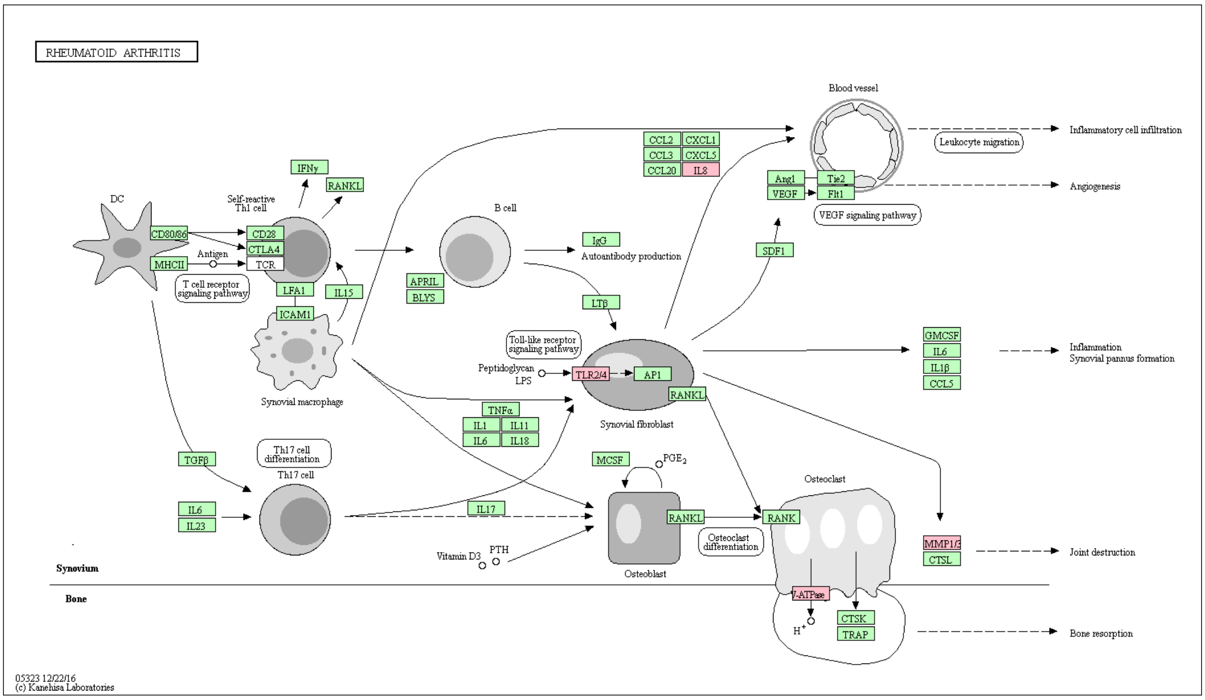

Figure S5. Overlapping differential proteins in KEGG pathway of Rheumatoid arthritis.

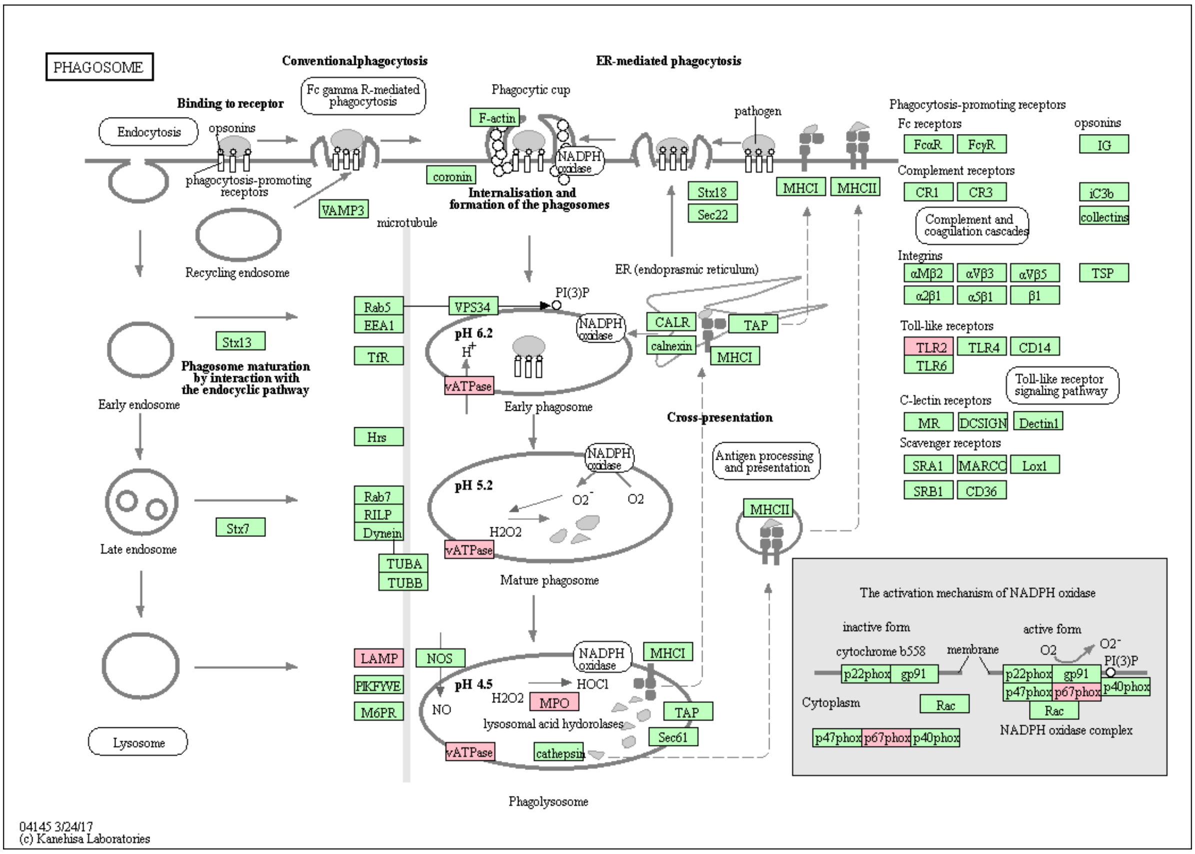

Figure S6. Overlapping differential proteins in KEGG pathway of Phagosome.
